# Short‐term starvation activates AMPK and restores mitochondrial inorganic polyphosphate, but fails to reverse associated neuronal senescence

**DOI:** 10.1111/acel.14289

**Published:** 2024-08-05

**Authors:** Luca Tagliafico, Renata T. Da Costa, Lavinia Boccia, Sheida Kavehmoghaddam, Bryan Ramirez, Malgorzata Tokarska‐Schlattner, Ernest R. Scoma, Vedangi Hambardikar, Tommaso Bonfiglio, Irene Caffa, Fiammetta Monacelli, Uwe Schlattner, J. Nicholas Betley, Alessio Nencioni, Maria E. Solesio

**Affiliations:** ^1^ Department of Biology and Center for Computational and Integrative Biology Rutgers University Camden New Jersey USA; ^2^ Department of Internal Medicine and Medical Specialties (DIMI) University of Genoa Genoa Italy; ^3^ Department of Biology University of Pennsylvania Philadelphia Pennsylvania USA; ^4^ Laboratory of Fundamental and Applied Bioenergetics Grenoble Alpes University Saint‐Martin‐d'Hères France; ^5^ IRCCS Ospedale Policlinico San Martino Genoa Italy

**Keywords:** dietary restriction, fasting, inorganic polyphosphate, metabolism, mitochondria, polyP, senescence

## Abstract

Neuronal senescence is a major risk factor for the development of many neurodegenerative disorders. The mechanisms that drive neurons to senescence remain largely elusive; however, dysregulated mitochondrial physiology seems to play a pivotal role in this process. Consequently, strategies aimed to preserve mitochondrial function may hold promise in mitigating neuronal senescence. For example, dietary restriction has shown to reduce senescence, via a mechanism that still remains far from being totally understood, but that could be at least partially mediated by mitochondria. Here, we address the role of mitochondrial inorganic polyphosphate (polyP) in the intersection between neuronal senescence and dietary restriction. PolyP is highly present in mammalian mitochondria; and its regulatory role in mammalian bioenergetics has already been described by us and others. Our data demonstrate that depletion of mitochondrial polyP exacerbates neuronal senescence, independently of whether dietary restriction is present. However, dietary restriction in polyP‐depleted cells activates AMPK, and it restores some components of mitochondrial physiology, even if this is not sufficient to revert increased senescence. The effects of dietary restriction on polyP levels and AMPK activation are conserved in differentiated SH‐SY5Y cells and brain tissue of male mice. Our results identify polyP as an important component in mitochondrial physiology at the intersection of dietary restriction and senescence, and they highlight the importance of the organelle in this intersection.

## Graphical abstract

In this manuscript, Tagliafico et al. address the relationship between the levels of inorganic polyphosphate (polyP) and the onset of neuronal senescence. Moreover, they show that short‐term starvation restores depleted levels of polyP and activates AMPK; however, this is not sufficient to reverse the increased senescence associated to decreased polyP. The study digs into the role of mitochondrial physiology in the intersection between dietary restriction and senescence, and it proposes polyP as an important component of this intersection.

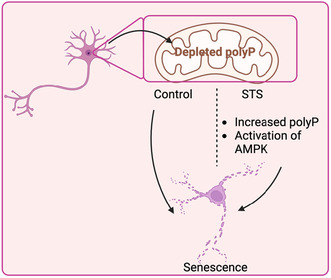

**Abbreviations**
ADP: Adenosine diphosphate
AMP: Adenosine monophosphate
AMPK: AMP‐activated protein kinase
ATP: Adenosine triphosphate
BSA: Bovine serum albumin
CCCP: Carbonyl cyanide m‐chlorophenylhydrazone
CREB: cAMP Response Element‐Binding Protein
Ctrl: Control
DAPI: 4′,6‐diamidino‐2‐phenylindole
DMEM: Dulbecco's Modified Eagle medium
DMSO: Dimethyl sulfoxide
Drp1: Dynamin‐related protein 1
dSH‐SY5Y: Differentiated SH‐SY5Y cells
EDTA: Ethylenediaminetetraacetic acid
EGTA: Ethylene glycol‐bis(2‐aminoethylether)‐N,N,N′,N′‐tetraacetic acid
ETC: Electron transfer chain
FBS: Fetal bovine serum
HBSS: Hank's Balanced salt solution
HPLC: High‐performance liquid chromatography
IACUC: Institutional animal care and use committee
IPA: Ingenuity Pathway Analysis
K2CO3: Potassium carbonate
KCl: Potassium chloride
LC3: Microtubule‐associate protein 1A/1B‐ligth chain 3
LDH: Lactate deshydrogenase
MgCl2: Magnesium chloride
MitoPPX: cells expressing the PPX enzyme in their mitochondria
NIH: The National Institutes of Health
OCR: Oxygen consumption rate
OXPHOS: Oxidative phosphorylation
pAMPK: Phospho‐AMPK (Thr172)
pAMPK: Phospho‐AMPK (Thr172)
PBS: Phosphate‐buffered saline
PCAs: Principal component analysis
pCREB: Phospho‐CREB (Ser133),
pDrp1: Phospho‐Drp1 (Ser616)
PMSF: Phenylmethylsulfonyl fluoride
PolyP: Inorganic polyphosphate
PPN: Endopolyphosphateses
PPX: Exopolyphosphatases
PVDF: Polyvinylidene fluoride
RA: Retinoic acid
ROS: Reactive oxygen species
SEM: Standard error of the mean
STS: Short‐term starvation
THB: Tissue homogenizing buffer
Tris‐HCl: Tris(hydroxymethyl)‐1,3‐propanediol hydrochloride
Wt: Wild‐type

## 1 INTRODUCTION

Senescence, which is characterized by a permanent proliferation arrest (Di Micco et al., 2021), is a major hallmark of aging (Lopez‐Otin et al., 2013), as well as a major contributor to the onset of many human diseases, including the major neurodegenerative disorders (McHugh & Gil, 2018). While the mechanisms driving mammalian cells to senescence are complex and still remain incompletely described, mitochondrial dysfunction, including bioenergetic dysregulation, seems to be crucially involved in these mechanisms (Miwa et al., 2022). In fact, dysregulated oxidative phosphorylation (OXPHOS) and increased generation of reactive oxygen species (ROS) have already been demonstrated in many aging‐related pathologies, such as Parkinson's and Alzheimer's disease (Solesio et al., 2012; Solesio et al., 2018).

The positive effects of dietary restriction on mammalian lifespan were first reported in 1935 by Crowell (1935). Since that seminal work, many other researchers have contributed to increasing our knowledge in this field. In fact, in the last few decades, caloric restriction and fasting (jointly referred to as dietary restriction) have been proposed as valid and powerful tools to prevent and/or delay senescence, aging, and aging‐related pathologies (Pletcher et al., 2002; Waziry et al., 2023; Weindruch & Walford, 1982). Interestingly, the protective effects of dietary restriction against senescence have been proposed to be mediated, at least partially, by the regulation of mitochondrial physiology, including bioenergetics (Deus et al., 2020). However, the molecular mechanisms that explain the protective effects of dietary restriction on mitochondrial bioenergetics still remain far from being completely understood. For example, in the cardiovascular system, dietary restriction improves mitochondrial function via regulation of OXPHOS, and it decreases oxidative stress (Savencu et al., 2021). Still, some studies suggest that, in rodents, this molecular mechanism could be extremely influenced by the sex, the type of tissue and ROS assayed, the length of dietary restriction, etc. (Walsh et al., 2014). Moreover, some extramitochondrial components could also be involved in the regulation of mitochondrial bioenergetics exerted by dietary restriction. This is the case of the AMP‐activated protein kinase (AMPK), a key energy gauge in the mammalian cell crucially involved in the maintenance of mitochondrial physiology (PMID: 28974774), which is activated by dietary restriction, and which activation has shown protective effects against senescence (Han et al., 2016; Weir et al., 2017).

Inorganic polyphosphate (polyP) is an ancient and ubiquitous polymer, which is well‐conserved through evolution. It is present in every tissue from every studied organism (Kornberg et al., 1999). However, in mammalian cells, we and others have already described a high colocalization between polyP and mitochondria (Abramov et al., 2007; Solesio, Demirkhanyan, et al., 2016). This polymer is formed by chains of orthophosphates linked together by high‐energy bonds, which are structurally identical to those found in ATP and ADP. In prokaryotes and simple eukaryotes, the enzymes in charge of the synthesis and the degradation of polyP are well described (polyP kinases; and endopolyphosphateses—PPN—and exopolyphosphatases—PPX—respectively (Lichko et al., 2003; Sethuraman et al., 2001; Wurst & Kornberg, 1994)). However, their homologs have not been found so far in mammals, even if some enzymes have already shown to be involved in the metabolism of polyP in these organisms. The regulation of all these enzymes is closely related to be bioenergetics status of the cells. For example, the mitochondrial ATP synthase has been proposed to be involved in the metabolism of polyP (Baev et al., 2020), and its role on this metabolism could be regulated by Prune (Scoma et al., 2023), which is a cAMP phosphodiesterase. Moreover, it has been recently discovered that Nudt3 can degrade polyP, and that the regulation of the levels of polyP by this enzyme mediates the oxidative stress response in mammalian cells (Samper‐Martin et al., 2021). However, likely, further enzymes are involved in the mammalian metabolism of polyP.

Despite the apparent complexity of the metabolism of polyP in mammals, prevailing evidence, including the molecular structure of polyP and its high mitochondrial levels (Angelova et al., 2014; Kornberg, 1995; Kornberg et al., 1999; Kumble & Kornberg, 1995; Rao et al., 2009; Solesio, Elustondo, et al., 2016), suggest a close relationship between the metabolism of polyP and the regulation of OXPHOS. Accordingly, a regulatory role for polyP in bioenergetics has already been proposed and studied in different organisms, including mammalian samples (Guitart‐Mampel et al., 2022; Hambardikar et al., 2022; McIntyre & Solesio, 2021; Muller et al., 2019; Müller et al., 2017; Solesio et al., 2020, 2021; Solesio, Demirkhanyan, et al., 2016). For example, in these organisms, we have already demonstrated the potent effects of polyP in the regulation of OXPHOS and oxidative stress (Guitart‐Mampel et al., 2022; Hambardikar et al., 2022; Solesio et al., 2021), as well as in the maintenance of calcium homeostasis (Solesio et al., 2020; Solesio, Demirkhanyan, et al., 2016). Moreover, polyP may also be involved in mammalian cell signaling (Da Costa & Solesio, 2023), a process closely related to bioenergetics. Considering all this, the study of the effects of polyP on mitochondrial function may shed new light on the mechanisms involved in the positive effects of dietary restriction in mammalian senescence.

To conduct our study, we used our recently generated and fully characterized MitoPPX cells (Guitart‐Mampel et al., 2022; Hambardikar et al., 2023), that is, cells enzymatically depleted of mitochondrial polyP by the ectopic expression of the PPX enzyme on mitochondria (similar approaches have been used by other researchers to target PPX to other organelles (Khong et al., 2020)). Specifically, we used wild‐type (Wt) and MitoPPX‐differentiated SH‐SY5Y (dSH‐SY5Y) cells. dSH‐SY5Y are known to present a neuron‐like phenotype, which is a more physiological model to conduct neuronal studies than the undifferentiated form of the cells, in which bioenergetics might be affected due to its cancer origins. Moreover, the regulatory role of polyP in neuronal bioenergetics has already been proposed (Borden et al., 2021). Samples were analyzed under control conditions (Ctrl) and after short‐term starvation (STS). We also used murine brains obtained from animals under control conditions and after intermittent fasting, to corroborate some of our results. Our results show the important role played by polyP in the mitochondrial intersection between the regulation of neuronal senescence and dietary restriction, and they suggest an effect for the polymer in the activation of AMPK. Accordingly, modulating the metabolism of mammalian polyP could be a potent pharmacological target against aging‐related diseases, where increased senescence has been broadly described.

## 2 MATERIALS AND METHODS

### 2.1 Reagents

Dulbecco's Modified Eagle medium (DMEM) without glucose, DMEM/F12, penicillin/streptomycin, heat‐inactivated fetal bovine serum (FBS), Hank's balanced salt solution (HBSS), and trypan blue solution were purchased from Gibco‐Invitrogen (Carlsbad, California, USA). Geneticin (G418), trypsin, phosphate‐buffered saline (PBS), retinoic acid (RA), dimethyl sulfoxide (DMSO), ethanol, carbonyl cyanide mchlorophenylhydrazone (CCCP), oligomycin, antimycin A, and D‐glucose monohydrate were purchased from Sigma‐Aldrich (St. Louis, Missouri, USA). RIPA lysis and extraction buffer, Pierce BCA protein assay kit, and Pierce ECL Western blotting substrate were purchased from ThermoFisher Scientific (Waltham, Massachusetts, USA). Phenylmethylsulfonyl fluoride (PMSF), Pierce Hlt protease inhibitor and phosphatase inhibitor cocktails, Triton X‐100, potassium chloride (KCl), tris(hydroxymethyl)‐1,3‐propanediol hydrochloride (TRIS–HCl), sucrose, mannitol, magnesium chloride (MgCl_2_), ethylenediaminetetraacetic acid (EDTA), ethylene glycolbis(2‐aminoethylether)‐N,N,N′,N′‐tetraacetic acid (EGTA), Dnase I and Rnase A from bovine pancreas, β‐mercaptoethanol, methanol, Tween‐20; perchloric acid, potassium carbonate, and Trizma base were purchased from Sigma Aldrich (Sigma‐Aldrich, St. Louis, Missouri, USA). 4′,6‐Diamidino‐2‐Phenylindole (DAPI), and senescence bgalactosidase staining kit were purchased from Cell Signaling Technology (Danvers, Massachusetts, USA). Glycerol, and MitoTracker Green FM were purchased from Invitrogen (Carlsbad, California, USA). Laemmli sample buffer, Tris/glycine/SDS buffer, Tris/glycine Buffer, 12% Mini‐Protean TGX precast protein gels, PVDF membrane, and blotting grade blocker were purchased from Bio‐Rad (Hercules, California, USA). The LDH cytotoxicity detection kit was purchased from Roche Diagnostic GmbH (Mannheim, Germany). Short‐chain polyP was a gift from Dr. Toshikazu Shiba from Kitasato University, Tokyo, Japan.

### 2.2 Cell cultures

SH‐SY5Y cells were obtained from the American Type Culture Collection (Manassas, Virginia, USA), and grown following the instructions provided by the manufacturer, and as previously reported (Solesio et al., 2012; Solesio, Saez‐Atienzar, et al., 2013). SH‐SY5Y MitoPPX were generated and maintained in our laboratory as we previously reported (Guitart‐Mampel et al., 2022).

### 2.3 SH‐SY5Y differentiation

Seven thousand Wt cells per cm^2^, and 9000 MitoPPX cells per cm^2^ were plated in different supports, depending on the specific experiment for which they were planned to be used. We plated different number of cells at the beginning of the differentiation process because MitoPPX cells tend to grow slower. This strategy allowed us to have a similar number of cells at the end of the differentiation. To differentiate the cells, we followed the previously described protocol (da Costa et al., 2021; Kalinovskii et al., 2022; Korecka et al., 2013). Briefly, 48 h after seeding the cells in regular medium, this medium was replaced by DMEM/F12 containing 1% FBS and 10 μM RA. Cells were then maintained in this medium for 7 days. During this time, the medium was changed every 2–3 days.

### 2.4 STS

After differentiation, cells were cultured in STS medium (DMEM with glutamine and without glucose, completed with 1% FBS and penicillin/streptomycin, and supplemented with 540 mg/L D‐glucose). In the case of the control conditions, we used regular DMEM/F12, containing glutamine and sodium pyruvate, and also completed with 1% FBS and penicillin/streptomycin. In this second medium, the concentration of glucose was 3151 mg/L. The use of DMEM/F12 for the control conditions and DMEM with low glucose for the fasting medium has been used by other researchers in the field (including in studies conducted using SH‐SY5Y cells), as it contributes to mimic dietary restriction (Cui et al., 2020; Pak et al., 2022; Rizzo et al., 2017). After incubation in either STS or control medium, cells were washed with ice‐cold PBS and collected at 24, 48, and 72 h.

### 2.5 MitoTracker green and dark‐field imaging

Cells were imaged following the protocol provided by the manufacturer. Images were acquired using an EVOS Inverted Imaging Digital Microscope (LifeTechnologies, Carlsbad, California, USA). Contrast was adjusted using ImageJ (NIH, Bethesda, Maryland, USA).

### 2.6 Image quantification

Mitochondrial area, and average length of mitochondrial branches were quantified in single cells using the ImageJ software (NIH, Bethesda, Maryland, USA). To conduct these quantifications, six cells form each of the biological triplicates were analyzed. Results in both cases were normalized to the values obtained from Wt control cells.

### 2.7 β‐Galactosidase activity assay

To test the onset of senescence in our cells, we assayed the activity of β‐galactosidase. To conduct this assay, we followed the protocol provided by the manufacturer (Cell Signaling technology, Danvers, Massachusetts, USA). To image the cells, we used an EVOS Inverted Imaging Digital Microscope (LifeTechnologies, Carlsbad, California, USA). Specifically, we used 25% of light and a 20× magnification objective. Images were adjusted and analyzed with ImageJ (NIH, Bethesda, Maryland, USA) to assess the mean intensity of the staining adjusted by the area occupied by the cells in each image. The results were standardized with the values obtained from the Wt cells cultured in regular medium.

### 2.8 Cell count and protein concentration assay

Seventy thousand Wt and MitoPPX cells were differentiated, and used either under control conditions or after STS. On the day of the experiment, cells were washed and scraped on ice‐cold PBS, collected, and counted at the indicated time points (24, 48, and 72 h). Subsequently, samples were spun down at 4°C for 5 min. Pellets were lysed using RIPA buffer supplemented with proteases inhibitors. Protein content was then quantified using the Pierce BCA Protein Assay Kit (ThermoFisher Scientific, Waltham, Massachusetts, USA), following the instructions provided by the manufacturer.

### 2.9 ATP/ADP ratio quantification

After removing the medium, differentiated cells were washed with ice‐cold PBS, then 0.5 mL of ice‐cold perchloric acid 0.6 N (3.5%) were added per culture dish (diameter = 10 cm). Cells were scraped and centrifuged for 2 min at 13,000 ***g*** and 4°C.

Supernatants were collected and neutralized for 30 min with K_2_CO_3_ (pH 6.5–7.5). Subsequently, samples were centrifuged for 10 min at 13,000 ***g*** and 4°C, and supernatants were recovered and frozen at −80°C. Samples were then analyzed using HPLC, following the previously published protocols (Pelosse et al., 2019; Tokarska‐Schlattner et al., 2021).

### 2.10 Quantification of cellular polyP

Cells were washed and scraped on ice with cold PBS and spun down at 4°C for 5 min. Cell pellets were resuspended in 25 μL of lysis buffer (30 mM Tris–HCl, pH 7.4, 200 mM KCl, 0.5% Triton, 1× protease inhibitor and 1 mM PMSF) and incubated for 15 min at 4°C, vortexing in between. Samples were then sonicated (QSONICA sonicator, Newtown, Connecticut, USA) on ice at 30% amplitude for three cycles with 25 s on and 25 s off, and centrifuged for 10 min at 18,000 ***g*** and 4°C. Subsequently, supernatants were collected, and protein concentrations were determined using BCA assay. Samples were then brought to a final protein concentration of 0.1 μg/μL in a buffer containing 75 mM sucrose, 225 mM mannitol, and 5 mM Tris‐HCl pH 7.4, 15 mM of MgCl_2_; and 1 mg/mL of DNase and RNase. After that, samples were incubated for 40 min at 37°C. Once finished this incubation, they were placed on ice, and 1 mM EDTA and 1 mM EGTA were added. Fifty microliter of each sample were added to each well of a black 96‐well plate in triplicates. Afterward, 10 μM DAPI was added to the samples, and fluorescence intensity was measured at 415/550 nm using a ClarioStar multifunctional microplate reader (BMG LABTECH, Ortenberg, Germany). The levels of polyP were standardized by the levels of the polymer in Wt cells in regular medium.

### 2.11 Mass spectrometry

Proteomics analysis, including database search, was conducted by the Rutgers Center for Advanced Biotechnology and Medicine (Mass Spectrometry Facility), following their standard protocols. Biological quadruplicates were used to conduct these experiments.

Data were shared with our laboratory, and means and *p* values were calculated. Values were then plotted following a similar approach to that previously used in our laboratory (Gasparyan et al., 2023; Guitart‐Mampel et al., 2022). Specifically, heat maps and principal component analysis (PCAs) were plotted using MetaboAnalyst (https://www.metaboanalyst.ca/). Features with >30% missing values were removed, and values within the range were estimated using k‐nearest neighbors. Normalization was then conducted using the median, and values were log‐transformed. For the volcano plots, a fold change threshold of two and a *p* value threshold of 0.05 were used. Subsequently, Ingenuity Pathway Analysis (IPA, Qiagen, Hilden, Germany) was used to further analyze the data and to create predictions. Only values with *p* values higher than 0.05 were used for the IPA analysis. Diseases and functions which are relevant for this manuscript were selected and plotted based on their z‐scores, using Microsoft Excel. The complete set of data is included in the Supplementary Sheet. Raw data is uploaded to MassIVE and publically avalaible (MSV000095282).

### 2.12 Immunoblotting

Immunoblots were conducted following our previously published protocol (Solesio et al., 2021). We used the following primary antibodies from Cell Signaling Technology (Danvers, Massachusetts, USA): anti‐P‐Drp1 Ser^616^ (rabbit, cat. number 3455), anti‐TOM20 (rabbit, cat. number 42406), anti‐Mitofusin‐2 (rabbit, cat. number 9482), anti‐Bax (rabbit, cat. number 5023), anti‐P‐AMPKα Thr^172^ (rabbit, cat. number 2535), anti‐AMPKα (rabbit, cat. number 2532), anti‐phospho‐CREB Ser^133^ (rabbit, cat. number 9191), and anti‐CREB (rabbit, cat. number 4820). Moreover, anti‐Parkin (mouse, cat. number sc‐32282) and anti‐β‐actin (mouse, cat. number 3700) were purchased from Santa Cruz Biotechnology (Dallas, Texas, USA). Anti‐Tom20 (Danvers, Massachusetts, USA), and anti‐total human OXPHOS cocktail (mouse, cat. number ab110411) and anti‐LC3B (rabbit, cat. number ab192890) were purchased from AbCam (Cambridge, Cambridgeshire, UK). All the incubations with primary antibodies were conducted overnight at 4°C. Anti‐rabbit and anti‐mouse secondary antibodies (cat. numbers 1706515 and 1706516, respectively) were purchased from Bio‐Rad (Hercules, California, USA). All the incubations with secondary antibodies were conducted at room temperature for 1 h. Unless otherwise stated, all the equipment and materials used to conduct the immunoblots were purchased from Bio‐Rad (Hercules, California, USA). All the quantified uncropped membranes from all the provided immunoblots are included in a Supplementary File.

### 2.13 Mice growth and maintenance

The protocol for using these mice was approved by the University of Pennsylvania IACUC. Twenty‐four six‐month‐old C57BL/6J mice (12 female and 12 male) were used to conduct the experiments included in this manuscript (Jackson Laboratory, Bar Harbor, Maine, USA). Mice were randomized to cages and divided into two groups: control and treatment. Mice included in the treatment group were subjected to a 48‐h fasting (an in vivo version of STS, water was provided ad libitum) once a week. The treatment was repeated on three consecutive weeks. Control mice had access to an ad libitum diet. During the fasting period, mice included in the treatment group were isolated on specific cages. Body weight was measured immediately before, during, and after fasting. If a mouse had a weight loss higher than 20%, fasting was interrupted, and the specific mice was returned to an ad libitum diet. At the end of the third cycle, after 48 h of fasting, glucose and ketone bodies were assessed in mice from both groups. Blood to conduct these assays was extracted from a small tail snip. Specifically, one drop of blood was placed on a glucose and ketone test strip, blood was analyzed using GlucoMen Areo 2 k (Menarini, Florence, Italy). Subsequently, mice of both groups were euthanized, and brains were collected.

A follow‐up experiment was conducted, using the same protocol as in the previous experiments, and including six male, and six female C57BL/6J mice. In this case, glucose levels were assayed again at the baseline, and also immediately before the last fasting cycle. Lactate levels were also assayed at the end of the experiment, using Lactate Plus (Nova Biomedical Corporation, Waltham, Massachusetts, USA). Lastly, a glucose tolerance test was performed following the methodology described in Benede‐Ubieto et al. (2020).

### 2.14 PolyP assay levels in mice brains

Brains were weighed and lysed. Specifically, for every 102 mg of weight, 600 μL of lysis buffer was added. The lysis buffer was the same as the one used for the assay of DAPI‐polyP in total cells. After homogenizing the samples, brains were frozen at −80°C and thawed for three times to ensure lysis. Subsequently, samples were sonicated on ice at 30% amplitude for three cycles with 50 s on and 30 s off (QSONICA Sonicator, Newtown, Connecticut, USA). Lastly, samples were centrifuged for 10 min at 18,000 ***g*** and 4°C, and supernatants were recovered. To assay the levels of polyP in these samples, we followed the same protocol as in the case of the cells.

### 2.15 Immunoblotting in mice brains

For every 100 mg of brain weight, 1 mL of tissue homogenizing buffer (THB) was added. THB was composed of 20 mM Tris base pH 7.4, 250 mM sucrose, and 1 mM EDTA and EGTA. Just before conducting the experiments, 1 mM PMSF and protease and phosphatase inhibitor cocktails were added to THB. Samples were then homogenized on a ice/water bath, and BSA protein quantification was conducted, also in the same bath. Equal amounts of protein were used for immunoblotting. All the quantified membranes are included uncropped in a Supplementary File.

### 2.16 High‐resolution respirometry

High‐resolution respirometry was conducted using an Oroboros O2K Oxygraph (Oroboros Instruments, Innsbruck, Austria), and following the protocol previously described by da Costa et al. (2021). Briefly, cellular respiration was assessed using 2 × 10^6^ cells per chamber at 37°C, while the samples were stirred in Mir05 medium (Oroboros Instruments, Innsbruck, Austria). To access different respiratory states, 0.5 μM oligomycin, 1.5 μM CCCP, and 0.5 μM antimycin A were added to the cells. Oxygen consumption rate (OCR) was recorded using DataLab 4 software (Oroboros Instruments, Innsbruck, Austria), and it is expressed as pmol of O_2_/s/10^6^ cells. All the respiratory states were subsequently corrected for non‐mitochondrial respiration.

### 2.17 Mitochondrial isolation

Mitochondrial isolation in Wt and MitoPPX dSH‐SY5Y cells was conducted following our previously published protocols (Fossati et al., 2016; Solesio et al., 2018).

### 2.18 Cytotoxicity assay

Cytoxocity was assayed using the LDH kit and following the instructions provided by the manufacturer. Signal was acquired using a ClarioStar multifunctional microplate reader (BMG LabTech, Ortenberg, Germany). Obtained values were normalized by those obtained from Wt control in each experiment.

### 2.19 Statistical analysis

Detailed explanation of the analysis of the mass spectrometry data are included in the specific section of the results. Regarding the rest of the experiments, all data are presented as mean and standard error of the mean (SEM) of at least three independent experiments. Statistical significance of the differences between groups was determined by one‐way or two‐way anova according to the number of independent variables. In the case of comparison between two groups, an unpaired *t* test was used. For longitudinal data, a paired t‐test was used. Outliers were identified and removed from the analysis according to Tukey's fences. The level of statistical significance was set at α = 0.05 (*p ≤ 0.05, **p ≤ 0.01, ***p ≤ 0.001, and ****p ≤ 0.0001). GraphPad Prism version 10 (San Diego, California, USA) was used to conduct the statistical analysis and plot the graphical representation.

## 3 RESULTS

### 3.1 72 h STS affects neuronal morphology and induces senescence in MitoPPX dSH‐SY5Y cells

**FIGURE 1:**
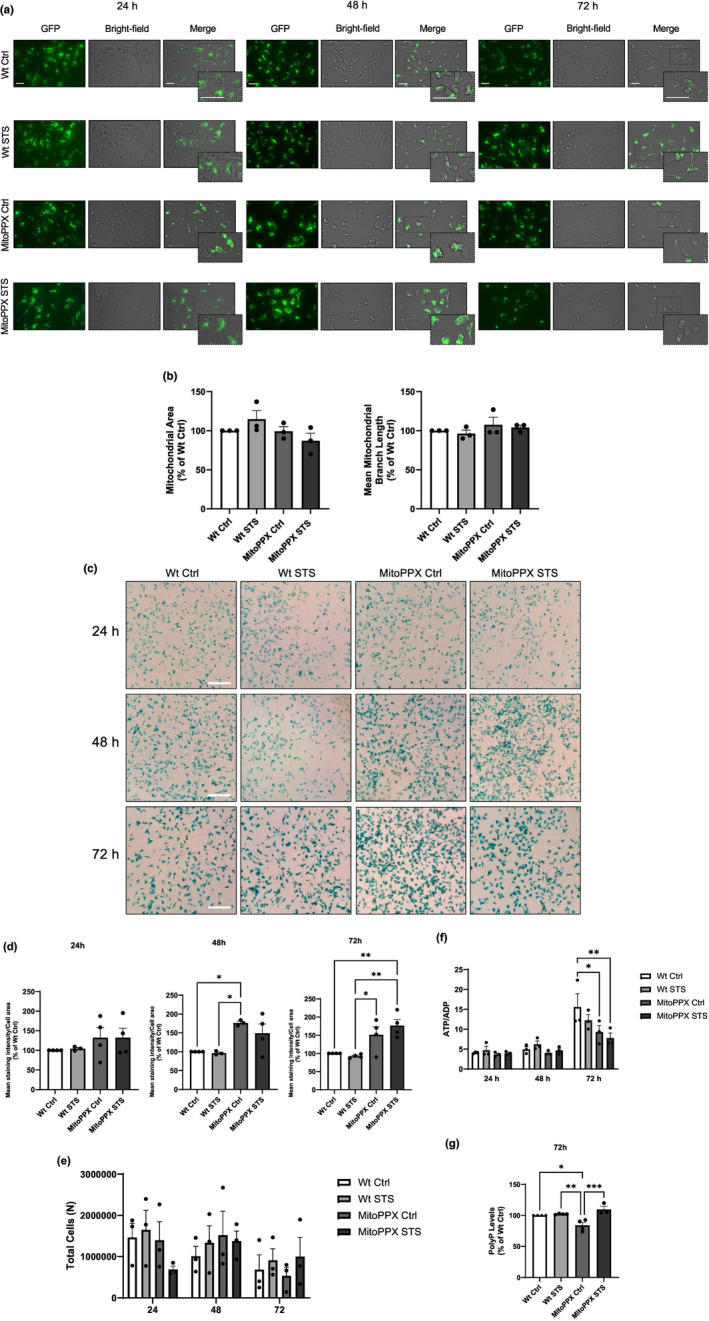
Depletion of mitochondrial polyP affects cellular morphology and it induces senescence in dSH‐SY5Y cells. 72 h of STS is able to restore mitochondrial polyP levels. (a) Representative images displaying mitochondrial and cellular morphology of dSH‐SY5Y Wt and MitoPPX cells. MitoTracker green fluorescence and dark‐field imaging were utilized to visualize mitochondrial and cellular morphology, respectively, in all experimental conditions. (b) Graph showing the quantification of the mitochondrial area, and the mean length of the mitochondrial branches in single cells. (c) Representative microscope images illustrating β‐galactosidase activity (increased activity of this enzyme has been broadly described as a marker of senescence). (d) Graphs presenting quantification of the intensity of β‐galactosidase staining in different experimental conditions. The mean intensity of the staining adjusted by the area occupied by the cells in each image, and normalized by the levels of the staining in Wt control cells is shown in the graphs. The experiment was conducted under control and 24, 48, and 72 h of STS in Wt and MitoPPX dSH‐SY5Y cells. Based on the findings from this figure, subsequent experiments in this study were conducted at STS 72 h. (e) Graph showing the quantification of the number of cells in all the conditions included in our studies. (f) Graphs showing the ATP/ADP ratio assayed in dSHYSY5Y Wt and MitoPPX cells, under control conditions and after STS. Cells were maintained in STS for 24, 48, and 72 h. (g) PolyP levels were assayed in all experimental conditions using the DAPI‐polyP assay. Please, note that significant differences are observed between Wt and MitoPPX dSH‐SY5Y cells. Moreover, 72 h STS is able to recover the decreased levels of the polymer in MitoPPX cells. Scale bar = 30 μM for images in panel A and 75 μM for images in panel c. Data are presented as mean ± SEM of at least three independent experiments. *p ≤ 0.05, **p ≤ 0.01, and ***p ≤ 0.001.

Our data suggest no major differences in mitochondrial morphology between Wt and MitoPPX dSH‐SY5Y cells at any of the time points that we assayed (Figure 1a,b). This morphology was visualized using MitoTracker green and fluorescence microscopy, and quantified using the average length of mitochondrial branches per cell, as well as the mitochondrial area. However, when cellular morphology was imaged using dark‐field microscopy, the characteristic neuron‐like shape of the dSH‐SY5Y cells seems to be affected by STS, time, and expression of MitoPPX.

To determine the presence of senescent Wt and MitoPPX dSH‐SY5Y cells under control conditions and after STS, βgalactosidase was detected using fluorescence, and the signal was visualized and quantified. To normalize the data, we plotted the intensity of the β‐galactosidase staining corrected by the area occupied by the cells. Our results show no major differences in senescence between Wt control and Wt STS dSH‐SY5Y cells at 24, 48, and 72 h. However, β‐galactosidase activity is significantly increased in MitoPPX cells after 48 h of STS (148.80% ± 25.04, Wt Ctrl was considered as 100%); and this effect is even more dramatic after 72 h (176.80% ± 16.73, Wt Ctrl was considered as 100%). In fact, MitoPPX cells show an increase of more than 50% in the activity of β‐galactosidase compared with the Wt samples, both under control conditions and after STS (Figure 1c,d). In all the conditions assayed, no significant differences in cell proliferation were found (Figure 1e).

One of the well‐described effects of STS in neurons is the increased activation of autophagy (Alirezaei et al., 2010). To corroborate that STS activates autophagy in dSH‐SY5Y cells, we assayed the LC3II/LC3I ratio in our cells. A significant increase in this ratio, which is of a similar magnitude in Wt and MitoPPX cells, is present after STS (Figure S1). Moreover, cellular protein content, assayed by BCA, does not show any significant differences between (i) Wt and MitoPPX, and (ii) control and STS dSH‐SY5Y cells at any of the time points that were assayed (24, 48, and 72 h; Figure S2a).

To address the status of energy metabolism in the experimental models, which is often compromised in senescence, we assayed the ATP/ADP ratio. Our results show that after 72 h under STS, MitoPPX cells have a significantly decreased ATP/ADP ratio compared to the Wt samples, both under control and STS conditions (9.28 ± 1.66, and 7.80 ± 1.27, respectively, vs. 15.57 ± 3.38 Wt Ctrl) (Figure 1f). Moreover, no increased cytotoxicity is observed at this time point in any of the conditions (Figure S2b). Based on all these results, we decided to use STS 72 h as the time point to conduct the rest of the experiments included in this manuscript. Interestingly, at this time point, we could not describe any significant differences in routine respiration, ATP‐linked respiration, and maximal respiration; when we used Oroboros to assay all of them (Figure S3).

To quantify the levels of polyP in the samples we used our recently optimized protocol (Hambardikar et al., 2023), which is an adaptation of the DAPI‐polyP method that was previously described by Solesio & Pavlov (2016). While STS 72 h does not affect the levels of polyP in the Wt samples, these levels are significantly decreased in MitoPPX cells under control conditions (83.94% ± 4.52, Wt Ctrl was considered as 100%), and they increased in the same cells when they were maintained in STS (109.70% ± 5.18, Wt Ctrl was considered as 100%) (Figure 1g). A similar trend is observed when the levels of polyP were assayed in isolated mitochondria, while no changes were present when this assay was conducted in cytoplasmic fractions (Figure S4).

### 3.2 The depletion of mitochondrial polyP and the presence of STS have significant effects on the proteome of dSH‐SY5Y cells

**FIGURE 2:**
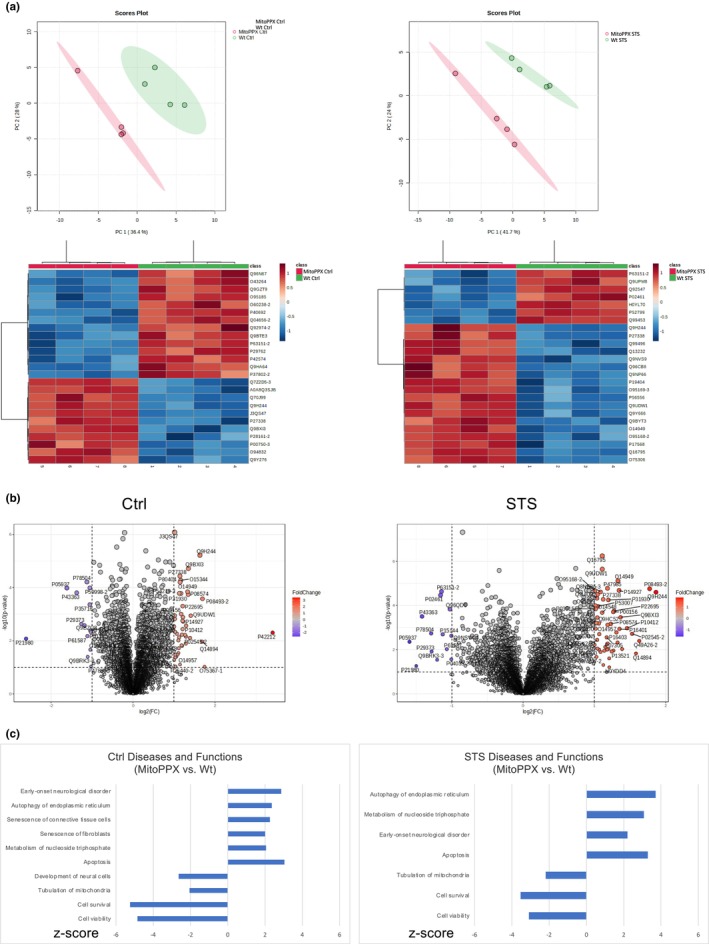
The proteome of Wt and MitoPPX dSH‐SY5Y cells is significantly different both under control conditions and after STS. (a) PCAs and heat maps (displaying the top 25 differently expressed proteins) showing the differences in the proteome between Wt and MitoPPX dSH‐SY5Y cells, under control conditions and after STS. (b) Volcano plot representations of the analysis of the same data. (c) Graphs presenting the z‐scores of the main diseases and functions relevant to this study and predicted to be differently affected in Wt and MitoPPX dSH‐SY5Y cells, under control and STS conditions. Predictions were conducted using IPA. Only data with *p* ≤ 0.05 were included. Mass spectrometry was conducted in biological quadruplicates.

Proteomics assays were conducted to address the effects of (i) STS, and (ii) the depletion of polyP and STS (which induces senescence—Figure 1c,d), in dSH‐SY5Y cells. To conduct our analysis, we calculated the MitoPPX Ctrl/Wt Ctrl and MitoPPX STS/Wt STS ratios. PCAs and heat maps were plotted, showing that the assayed conditions induce significant changes in the proteome of the compared experimental groups (Figure 2a, and Table S1). Volcano plot data are presented in Figure 2b. Predictions were created using IPA. Selected diseases and functions that were predicted to be differently affected in Wt and MitoPPX dSY‐SY5Y cells and to be significant in the context of this study were then plotted based on their *z*‐scores (Figure 2c). PCAs and heat maps obtained from the Wt STS/Wt Ctrl and the MitoPPX STS/MitoPPX Ctrl ratio are included in Figure S5.

### 3.3 The protective effects of polyP against senescence could be mediated by the regulation of the activity of AMPK

**FIGURE 3:**
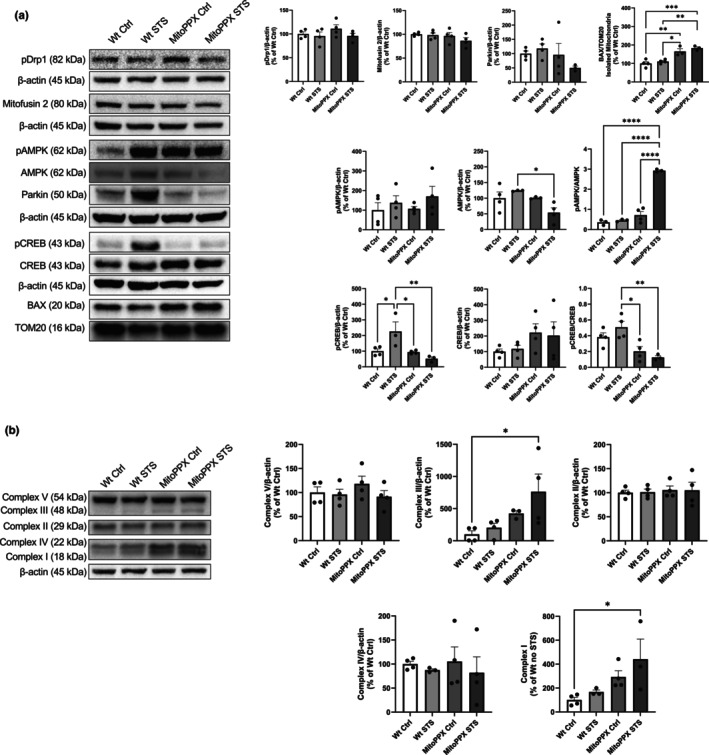
Effects of STS on the mitochondrial physiology of dSH‐SY5Y Wt and MitoPPX cells. (a) Representative immunoblots and quantification of the signal of some main proteins involved in the regulation of mitochondrial physiology. Note the increased levels of Bax in dSH‐SY5Y MitoPPX cells under both control conditions and after STS, and the significant rise in the pAMPK/AMPK ratio in dSH‐SY5Y MitoPPX cells after STS. (b) Representative immunoblots and quantification of the signal of the complexes of the mitochondrial ETC. Complexes I and III exhibit significant differences in MitoPPX cells under STS. Please, note that some membranes were re‐blotted for multiple antibodies and therefore, the same immunoblot showing the signal for β‐actin is used for more than one protein. All uncropped membranes that were quantified are included in a Supplementary File. Data are presented as mean ± SEM of at least three independent experiments. *p ≤ 0.05, **p ≤ 0.01, and ****p ≤ 0.0001.

Immunoblotting assays for some of the main proteins involved in the maintenance of mitochondrial physiology, such as pDrp1 (Ser^616^), mitofusin 2, or parkin, show no major differences in any of our experimental conditions. These proteins are crucially involved in the regulation of fission, fusion, and mitophagy, respectively. However, increased mitochondrial levels of Bax, are observed in MitoPPX cells, both under control conditions and after STS (165.20% ± 14.80 and +183.10% ± 6.62, respectively. Wt Ctrl was considered as 100%). Our data also show that the pAMPK (Thr^172^)/AMPK ratio is significantly increased in STS MitoPPX cells (2.93 ± 0.04 vs. 0.36 ± 0.08 Wt Ctrl, and 0.72 ± 0.18 MitoPPX Ctrl). Alterations in this ratio are usually related to bioenergetics dysregulation, and they can affect the regulation of cAMP Response Element‐Binding Protein (CREB) (Thomson et al., 2008). However, the pCREB (Ser^133^)/CREB ratio was decreased in MitoPPX cells, both under Ctrl and STS conditions, when compared to STS Wt (0.20 ± 0.06 and 0.13 ± 0.02, respectively vs. 0.51 ± 0.07 Wt STS, Figure 3a).

To further address the effects of polyP and STS in the regulation of mitochondrial physiology in Wt and MitoPPX dSH‐SY5Y cells, we assayed the protein levels of the complexes forming the electron transfer chain (ETC) in all our experimental conditions. Our data show only significant changes in complexes I and III. Specifically, in STS MitoPPX dSH‐SY5Y cells, the presence of both complexes I and III is increased when compared to the Wt Ctrl conditions (441.80% ± 167.70 and 762.50% ± 275.10), respectively. Wt Ctrl was considered as 100% (Figure 3b). However, no differences are present in the protein levels of any of the complexes between MitoPPX under control and STS conditions are observed.

### 3.4 PolyP levels and the pAMPK/AMPK ratio are also increased in C57BJ/6 male mice under intermittent fasting

**FIGURE 4:**
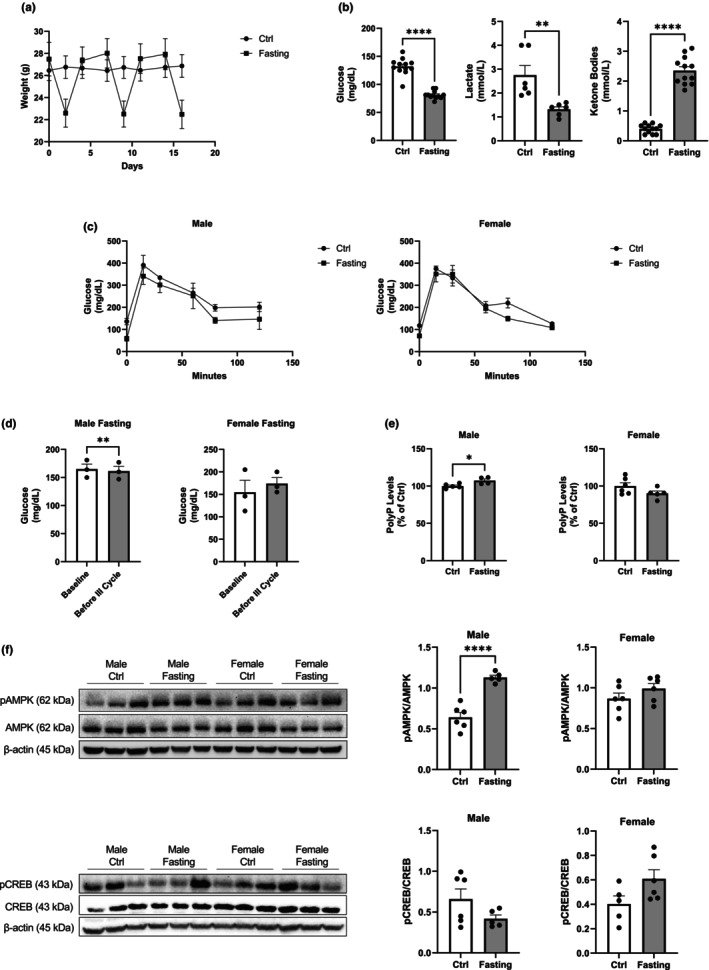
Intermittent fasting increases polyP levels in male C57BJ/6 mice. (a) Graph displaying the variation in mice weight during the different cycles of intermittent fasting. Measurements include both female and male C57BJ/6 mice. (b) Quantification of glucose and lactate levels, as well as of the presence of ketone bodies at the end of the final cycle of intermittent fasting. Also in this case, measurements include both female and male C57BJ/6 mice. (c) Graphs showing the results from the glucose tolerance test in each of the sexes. This test was conducted at the end of the third cycle of fasting. (d) Graphs showing the results from the assay of non‐fasting glucose levels at baseline and immediately before the last cycle of intermittent fasting. This parameter was evaluated independently for males and females. (e) Assay of polyP levels in the brains of male and female C57BJ/6 mice. Measurements were conducted immediately after euthanasia using the DAPI‐polyP method. (f) Representative immunoblots and quantification of the signal of the pAMPK/AMPK ratio, as well as of the pCREB/CREB ratio in male and female C57BJ/6 mice. Data are presented as mean ± SEM of at least three mice. *p ≤ 0.0.05, **p ≤ 0.01, and ****p ≤ 0.0001.

To extend our in vitro findings, we explored whether dietary restriction influences the levels of polyP. Three cycles of 48 h intermittent fasting were conducted in male and female C57BL/6 mice over 2 weeks, and their body weights were recorded (Figure 4a). Following those two weeks, the levels of glucose and lactate, as well as the presence of ketone bodies, and glucose tolerance in mice were analyzed (these analyses were conducted immediately after 48 h of fasting at the end of the third cycle of fasting). Our data show decreased glucose levels (81.08 ± 2.02 vs. 131.80 ± 4.33 mg/dL in Ctrl mice), and lactate levels (1.32 ± 0.11 vs. 2.75 ± 0.40 mmol/L in Ctrl mice); as well as increased presence of ketone bodies in these animals, compared to the control mice (2.35 ± 0.13 vs. 0.40 ± 0.04 mmol/L in Ctrl mice) (Figure 4b). Glucose tolerance was also analyzed, but no significant differences were observed in this parameter between the two experimental groups, in either of the sexes (Figure 4c). Furthermore, non‐fasting glucose levels at the baseline and immediately before the last cycle of intermittent fasting were evaluated. Our results show a reduction of this parameter in male mice (165.00 ± 8.96 mg/dL baseline vs. 161.30 ± 8.69 mg/dL) immediately before the last fasting cycle), but no significant differences in the case of the female mice (154.70 ± 26.91 mg/dL baseline vs.174.00 ± 13.45 mg/dL) immediately before the last fasting cycle (Figure 4d).

After the last cycle, mice were euthanized and brains were harvested to compare the levels of polyP between animals under control and fasting conditions. No significant differences were observed in polyP levels in female mice. However, these levels were significantly increased in males after fasting, similarly to what we observed in the MitoPPX cells (107.30% ± 2.27, Ctrl was considered as 100%) (Figure 4e). In addition, similarly to what was observed in cells, this effect was associated with the regulation of the activity of AMPK, but not of CREB (Figure 4f).

## 4 DISCUSSION

Mitochondrial dysfunction has been broadly described in aging and aging‐related pathologies (Fossati et al., 2016; Miwa et al., 2022; Solesio et al., 2012; Solesio et al., 2018; Solesio, Prime, et al., 2013; Solesio, Saez‐Atienzar, et al., 2013); and it is a known trigger of senescence (Martini & Passos, 2023). Accordingly, the beneficial effects of dietary restriction on mitochondrial physiology seem to be crucial to explain the protective outcomes of this strategy against neuronal senescence (Deus et al., 2020). However, the exact mitochondrial mechanism that underlies these protective effects remain still poorly understood.

Our studies were conducted using dSH‐SY5Y cells. These cells express different markers of mature neurons, including but not limited to synaptophysin, neuron‐specific enolase, and neuronal nuclei; and they lack expression of glial markers (Cheung et al., 2009; Encinas et al., 2000; Gimenez‐Cassina et al., 2006). To induce senescence, which was determined by the assay of the activity of the β‐galactosidase enzyme, we kept the cells in culture for up to 72 h after differentiation. During this time, cells were maintained under control or STS conditions. STS is a type of dietary restriction commonly used in mammalian models (Brandhorst et al., 2013; Cangemi et al., 2016; Qin et al., 2016). By assaying the increased levels of LC3 I/II in our samples after STS, we corroborated the validity of the model, as increased values of this ratio are a well‐known marker of dietary restriction (Green et al., 2022). With this experiment, we did not aim to address the presence of senescence in our cells because while some authors have described increased senescence‐associated autophagy (Young et al., 2009), others described the opposite process (Suelves et al., 2023).

Our findings show that, 72 h post‐differentiation, the enzymatic depletion of mitochondrial polyP affects cellular morphology and induces the onset of senescence in MitoPPX dSH‐SY5Y cells, while these effects are not observed in the Wt samples. Decreased levels of polyP have already been shown in aging mammalian organisms (Lorenz et al., 1997), and the protective effects of the polymer against amyloid‐induced mitochondrial toxicity, which is present in many neurodegenerative disorders, have also been described in different organisms (Cremers et al., 2016; Muller et al., 2017). Moreover, the literature is rich in examples showing the potent effects of polyP on the regulation of mitochondrial physiology (Angelova et al., 2016; Baev et al., 2017; Borden et al., 2021; Da Costa & Solesio, 2023; Guitart‐Mampel et al., 2022; Hambardikar et al., 2022; Osorio et al., 2022; Pavlov et al., 2010; Seidlmayer et al., 2012; Seidlmayer et al., 2019; Solesio et al., 2020; Solesio, Demirkhanyan, et al., 2016; Solesio, Elustondo, et al., 2016). Therefore, decreased levels of mitochondrial polyP could negatively affect the physiology of the organelle, and consequently promote the onset of senescence. While STS does not affect the levels of polyP in Wt dSH‐SY5Y cells, it increases those levels in MitoPPX cells, bringing them to values similar to those found in the Wt cells. However, this increase is not sufficient to revert senescence in MitoPPX cells. Accordingly, decreased ATP, which has been already described in senescence (Delfarah et al., 2019), and which based on our findings and previous studies (Solesio et al., 2021) could also be mediated by a shift from OXPHOS to glycolysis, is observed in MitoPPX cells, and it is not reverted by STS. These findings suggest that the effects induced by the depletion of mitochondrial polyP in senescence are not reversible by the simple increase of the levels of the polymer.

Mammalian senescence is associated with profound changes in the cellular proteome, including proteins exerting their physiological functions in mitochondria (Kim et al., 2023). Moreover, the regulatory effects of dietary restriction on the proteome have already been shown (Escobar et al., 2019). Therefore, to further assay the effects of polyP in the mechanism that links dietary restriction and neuronal senescence, we assayed proteomics in our samples. Our data show that the proteomes of Wt and MitoPPX dSH‐SY5Y cells are significantly different, both under control conditions and after STS, even if the total protein content is not affected. Moreover, IPA analysis of the mass spectrometry data allowed us to determine the important regulatory role of mitochondrial polyP in different senescence‐related pathways and diseases in which mitochondrial physiology is involved. For example, under control conditions, early onset of neurological disorders, apoptosis, and autophagy are increased in MitoPPX cells; while cell survival and viability are decreased in these cells. Moreover, STS is able to partially revert or ameliorate these effects, corroborating the protective effects of dietary restriction in senescence, but also that these effects are not powerful enough to revert the increased senescence induced by the enzymatic depletion of mitochondrial polyP. Our findings align with previous research showing that polyP is able to modulate the proteome of mammalian cells, via a mechanism that remains still unknown (Guitart‐Mampel et al., 2022); but that might be dependent on the length of the polymer (Bondy‐Chorney et al., 2020).

No significant effects are induced by the depletion of mitochondrial polyP on the main proteins involved in fission, fusion, and mitophagy in our models, even if we recognize that further experiments should be conducted to unequivocally determine this lack of effect. Previous studies show increased Drp1‐mediated mitochondrial tubulation in mammalian cells under STS (Rambold et al., 2011). The differences between the results from that study and our findings could be attributed to the specific type and the length of STS, which was determined to play a crucial role on mitochondrial elongation by the same authors (Rambold et al., 2011). In fact, they concluded that shorter times of STS and simultaneous depletion of multiple nutrients are most likely to induce mitochondrial elongation.

The depletion of mitochondrial polyP increases Bax levels within the organelle. Increased mitochondrial Bax was not reverted by STS, similarly to what is observed in the case of senescence. Increased mitochondrial Bax has been broadly demonstrated in many models of senescence; and it is not necessarily always associated to increased apoptosis in senescent samples (Childs et al., 2014; Hu et al., 2022; Victorelli et al., 2023). Therefore, it could be interpreted as a consequence of the increased senescence described in MitoPPX cells. The increased levels of polyP present in MitoPPX cells under STS induce an increased activation of AMPK, which could mediate some other effects exerted by STS in MitoPPX cells. Increased activity of AMPK has been already demonstrated in senescent cells as a protective mechanism against excessive mitochondrial dysfunction and, ultimately, cell death (Han et al., 2016). Therefore, in MitoPPX cells, this activation could be a consequence of the increased levels of ADP/AMP and the decreased levels of ATP, probably activated as a compensatory mechanism induced by STS, aiming to diminish the cellular damage induced by senescence. Specifically, polyP could induce an allosteric activation of AMPK, acting at the cystathionine beta‐synthase domain of the enzyme, similarly to what has been described for AMP or ADP (Coccimiglio & Clarke, 2020; Suter et al., 2006). However, further studies need to be conducted to elucidate the exact mechanism by which polyP modulates the activation of AMPK. One classical downstream effect of increased AMPK activity is the increased activation of CREB (Thomson et al., 2008). However, we do not observe this effect in MitoPPX cells after STS. It has been described that neurotrophins might be needed to activate CREB (Finkbeiner et al., 1997). The lack of these compounds in our STS medium could also explain our results.

The regulatory effects of polyP in OXPHOS have been described by us and others (Abramov et al., 2007; Hambardikar et al., 2022; Pavlov et al., 2010; Solesio et al., 2021), and the close relationship between the status of AMPK and that of OXPHOS is also known (Herzig & Shaw, 2018). Considering all this, as well as the potent effects that the depletion of mitochondrial polyP has in the mammalian proteome, we assayed the levels of the different complexes of the ETC in all our samples. While there is a significantly increased presence of complex I and complex III in STS MitoPPX cells compared to the Wt control samples, this increase is not present between control and STS dSHSY5Y MitoPPX cells. Therefore, it is not a consequence of the protective effects of STS in the cells. The increased levels of complexes I and III could be a consequence of the effects of polyP in the structure of the mitochondrial pores and channels (Solesio, Elustondo, et al., 2016), which are needed for the import of the subunits of ETC; as well as on the regulatory role of the polymer on proteostasis (Gray et al., 2014; Lempart et al., 2019; Yoo et al., 2018), including mitochondrial proteostasis (PMID: 39050895), which could affect the structure and the assembly of the subunits. In the case of complex III, the target of the used antibody is UQCRC2. The peptidase activity of this complex is crucial for the complex post‐transcriptional modifications needed for the assembly of this complex (Fernandez‐Vizarra & Zeviani, 2018). Increased levels of polyP induced by STS could affect this process. Complex I is the largest and most complex component of the ETC, therefore, changes in protein import and homeostasis could also have a major effect on it. However, further experiments will be needed to confirm these mechanisms.

Lastly, we conducted intermittent fasting in male and female mice to address whether dietary restriction in the brain of mammals induces a similar effect to STS in Wt dSHSY5Y cells, therefore affecting the levels of polyP and the activity of AMPK. The lack of animal models in which mitochondrial polyP is depleted prevented us from also addressing whether this lack induces the onset of senescence in mice. Our data show increased levels of polyP and activation of AMPK in brain extracted from male mice after intermittent fasting, similarly to what we observed in dSH‐SY5Y MitoPPX cells after STS, therefore corroborating the regulatory effects of dietary restriction of polyP levels and AMPK activation. However, we could not reproduce these results in female mice. From our knowledge, there are no previous studies in the field of polyP in which sex has been considered as a biological variable, especially considering that the number of studies conducted in mammals is extremely limited. However, it is known that the metabolism of phosphates (and that of calcium, which is closely related (Shaker & Deftos, 2000)) is different in mammalian females and males (Turner et al., 2023). Specifically, estrogen plays a crucial role in the metabolism of phosphates (Guttmann‐Rubinstein et al., 2010; Zhang et al., 2014), and therefore, probably in that of polyP. Accordingly, the differences observed in male and female mice could be a consequence of the differences in the metabolism of the polymer. Moreover, the body weight and fat mass of male and female mice are known to be different (Hong et al., 2009). This could also have an effect on the observed differences. Furthermore, the observed differences in cerebral polyP levels between male and female mice could also be part of a broader molecular mechanism underlying a different response to intermittent fasting in both sexes, which has been already proposed by some authors (Kennard et al., 2022; Saravanan et al., 2023). In fact, while no differences were observed between males and females on the non‐fasting glucose levels at baseline, when compared to those before the last cycle of fasting, this parameter significantly decreased in males, but it was not affected in females. These findings align with the previous literature in the field (Endo et al., 2023; Liu et al., 2019).

For the first time, we show the potent effects induced by the depletion of mitochondrial polyP in the onset of neuronal senescence. Increased senescence induced by the depletion of mitochondrial polyP is not reverted by STS. However, STS in dSH‐SY5Y MitoPPX cells is able to activate AMPK, probably as a compensatory mechanism aimed to revert the deleterious effects of this depletion. The fact that the STS‐induced increased levels of polyP and the consequent increased activation of AMPK are found in brain tissue from male mice after intermittent fasting suggests that the effects of this polymer in mammalian physiology are conserved in different species. Our findings pave the road for further studies in which the metabolism of polyP could be used as a promising target against neuronal senescence.

## AUTHOR CONTRIBUTIONS

Luca Tagliafico, Tommaso Bonfiglio, Irene Caffa, Fiammetta Monacelli, J Nicholas Betley, Alessio Nencioni, Uwe Schlattner, and Maria E Solesio conceived and designed the analysis. Luca Tagliafico, Renata T Da Costa, Lavinia Boccia, Sheida Kavehmoghaddam, Bryan Ramirez, Malgorzata Tokarska‐Schlattner, and Uwe Schlattner collected the data. Luca Tagliafico, Renata T Da Costa, Ernest R Scoma, Vedangi Hambardikar, Uwe Schlattner, J Nicholas Betley, and Maria E Solesio contributed to data analysis. Luca Tagliafico, Renata T Da Costa, and Maria E Solesio performed the analysis and wrote the paper. All authors have read and agreed to the published version of the manuscript.

## FUNDING INFORMATION

This project was mostly funded by the StartUp funds from Rutgers University to MES. Moreover, JNB and his team were supported by 1R01AG079877 and 1R01DK13339 (both to JNB) to conduct the experiments in which mice were involved. The stipend of Dr. Tagliafico was supported by the University of Genoa (Genoa, Italy).

## CONFLICT OF INTEREST STATEMENT

The authors declare no conflict of interest.

## Supporting information


Figures S1‐S5.

Data S1.

Data S2.

Table S1.

## Data Availability

Further information and requests for resources and reagents should be directed to and will be fulfilled by the Lead Contact, Dr. Maria E. Solesio (m.solesio@rutgers.edu). The analysis of the LC‐MS/MS data is included in an excel file as a Supplementary Datasheet. Raw data are uploaded to MassIVE, and it is publically accessible (MSV000095282).
