# Supplementary material for: Short‐term starvation activates AMPK and restores mitochondrial inorganic polyphosphate, but fails to reverse associated neuronal senescence
Source: Aging Cell. 2024 Aug 5;23(11):e14289. doi: 10.1111/acel.14289 (PMC11561667; doi:10.1111/acel.14289)

Supplementary Figure 1

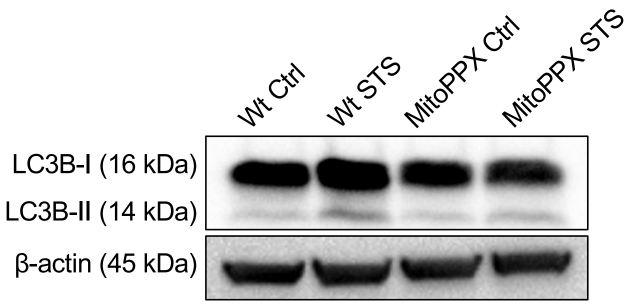

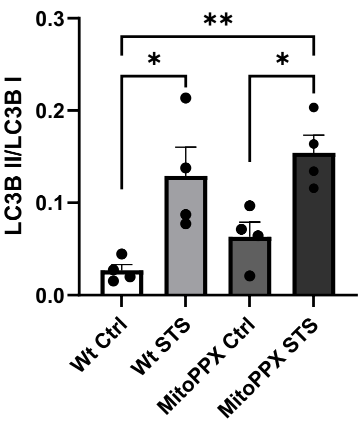

Supplementary Figure 2

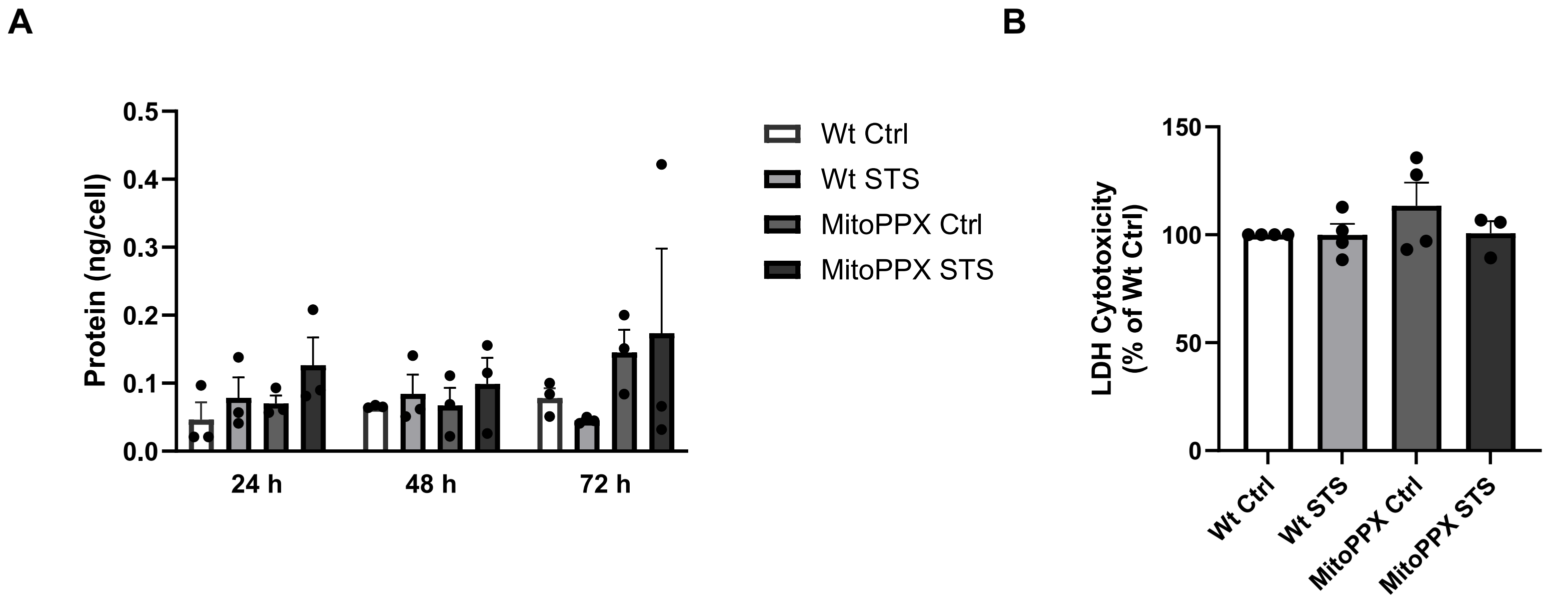

Supplementary Figure 3

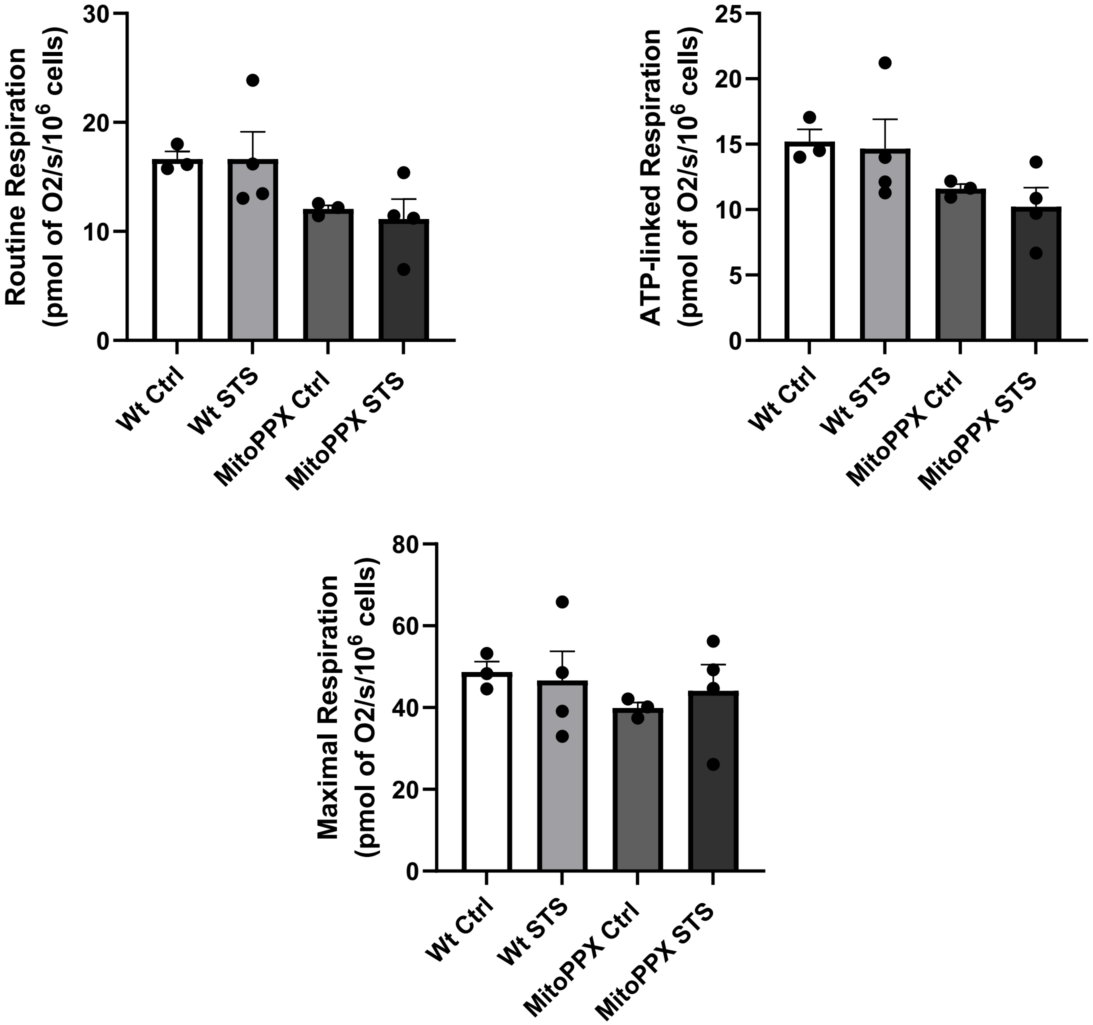

Supplementary Figure 4

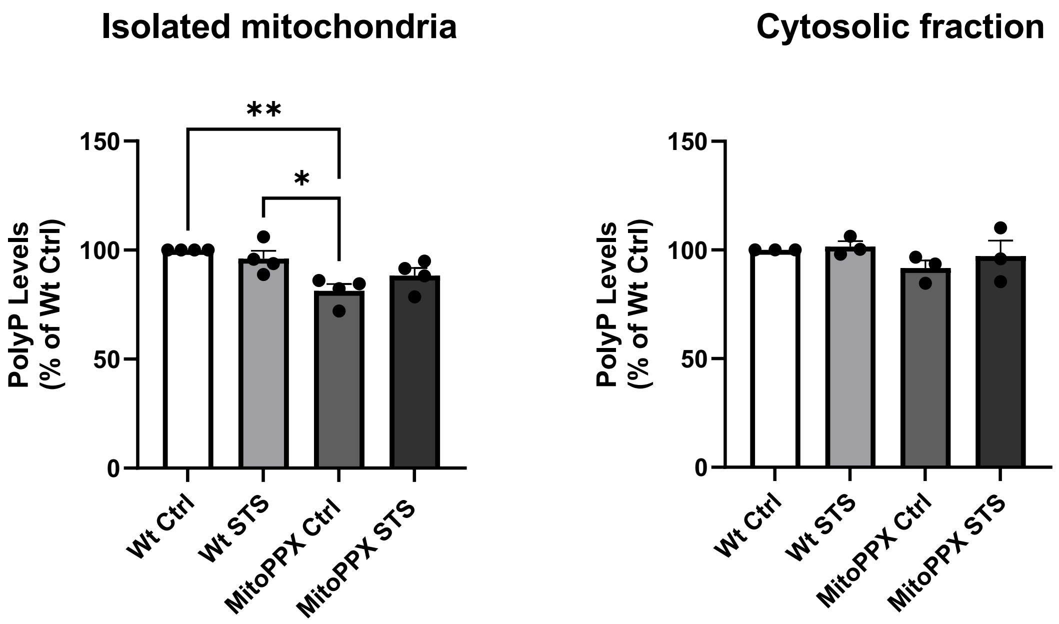

Supplementary Figure 5

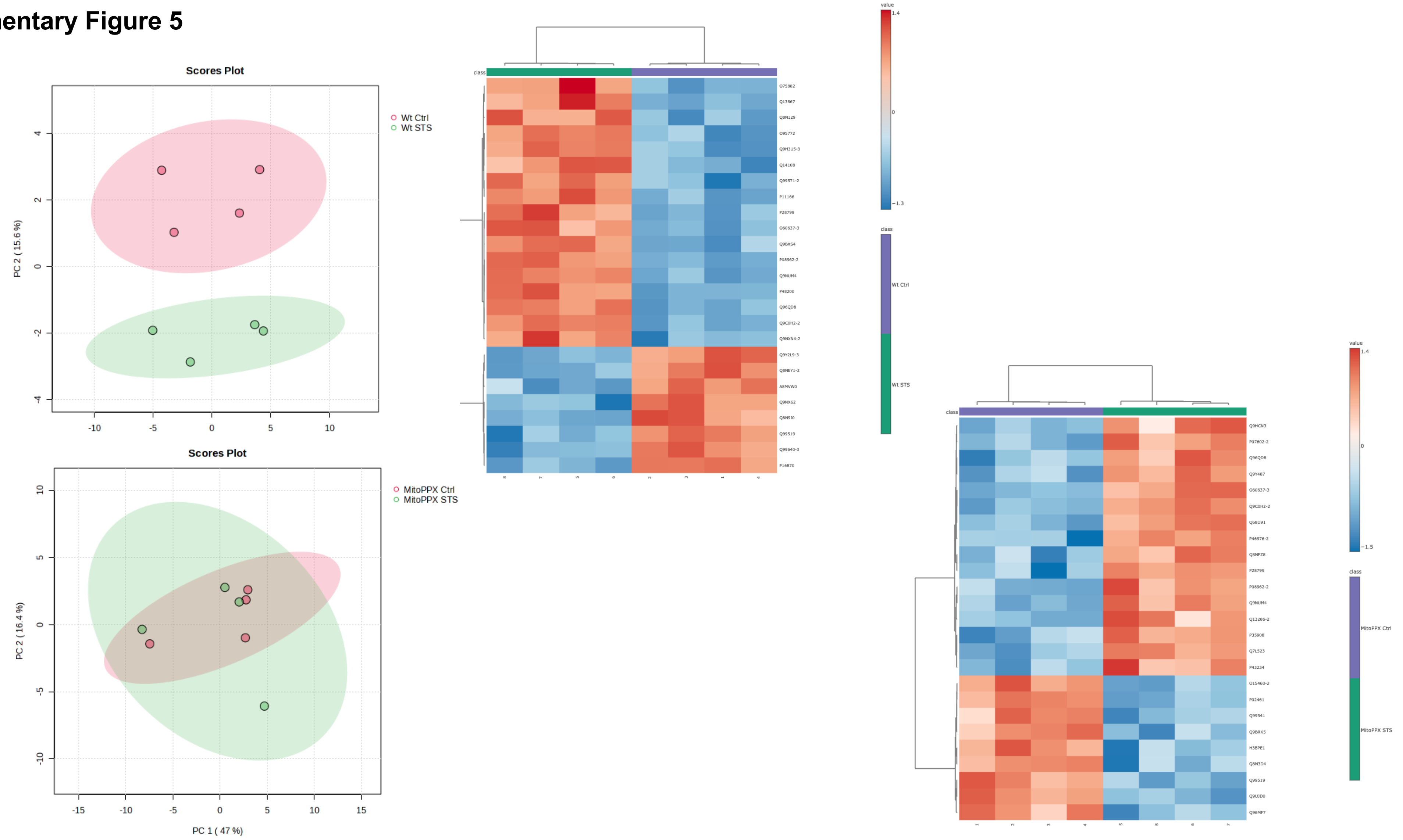

Supplement: Supplementary file 1 — Figures S1‐S5. [file ACEL-23-e14289-s003.pdf]
