# Supplementary material for: Short‐term starvation activates AMPK and restores mitochondrial inorganic polyphosphate, but fails to reverse associated neuronal senescence
Source: Aging Cell. 2024 Aug 5;23(11):e14289. doi: 10.1111/acel.14289 (PMC11561667; doi:10.1111/acel.14289)
Supplement: Supplementary file 2 — Data S1. [file ACEL-23-e14289-s001.pdf]

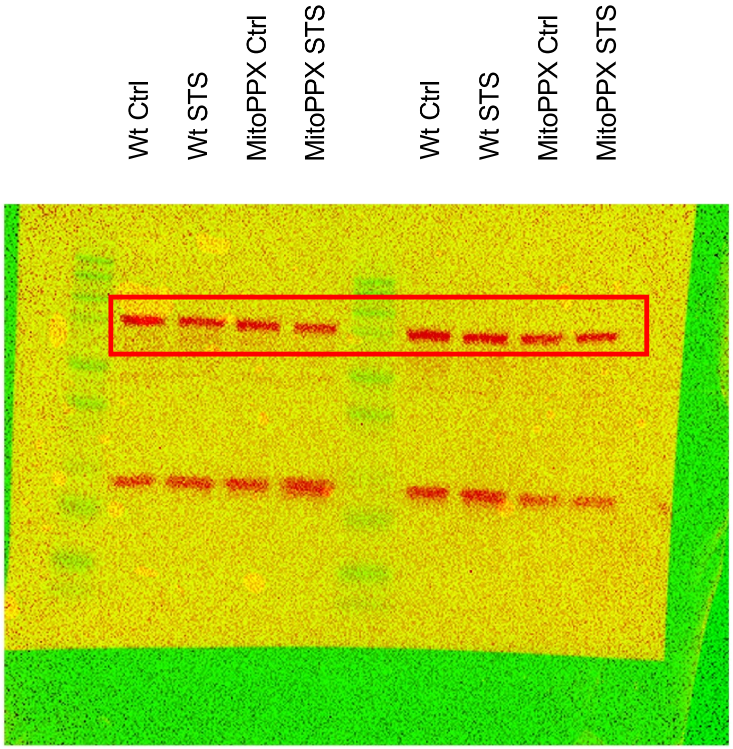

pDrp1 1

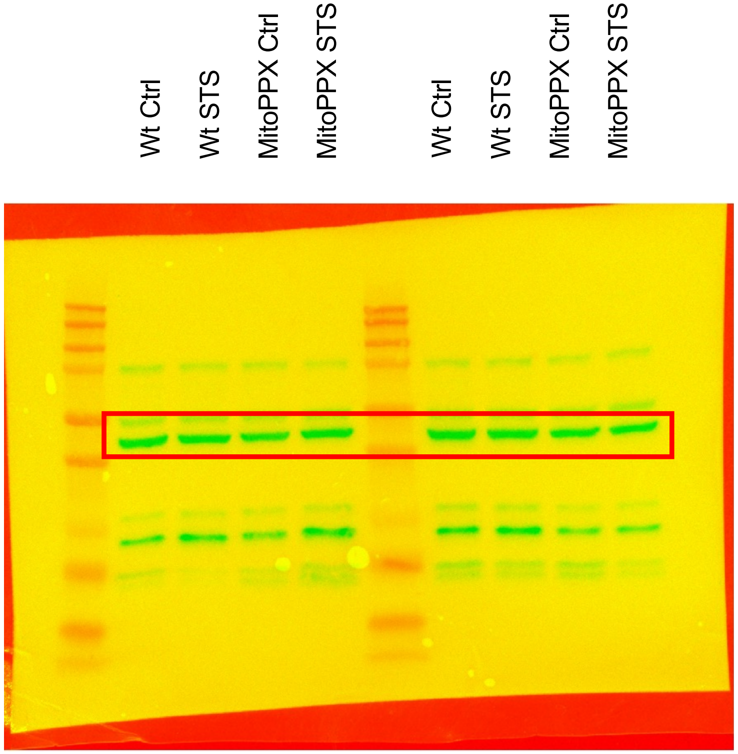

β-actin 1

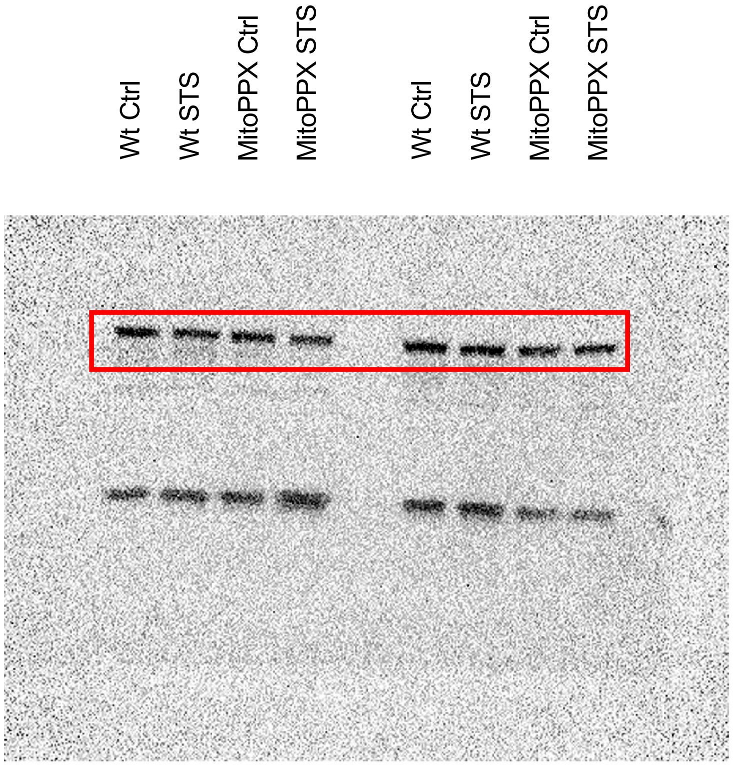

pDrp1 1

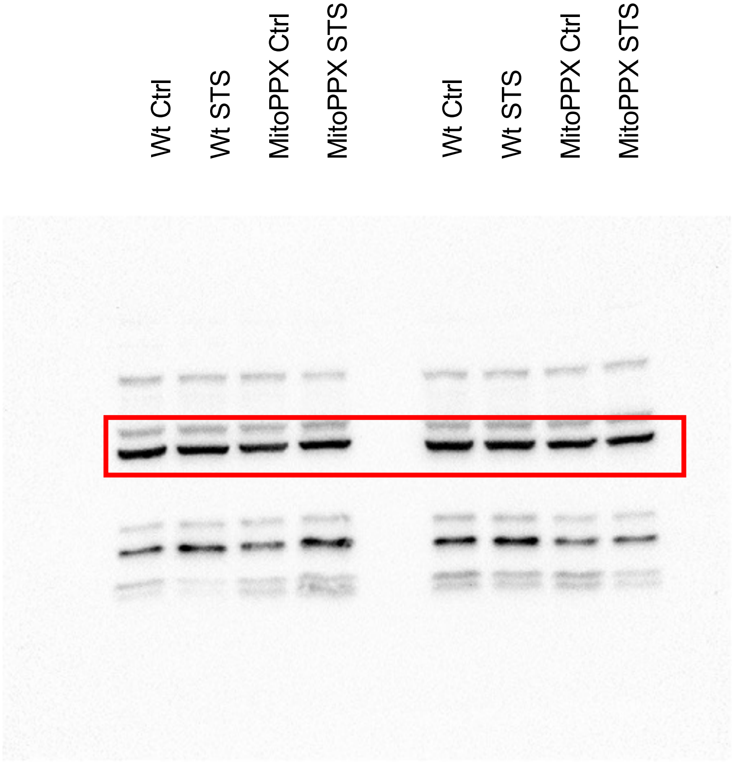

β-actin 1

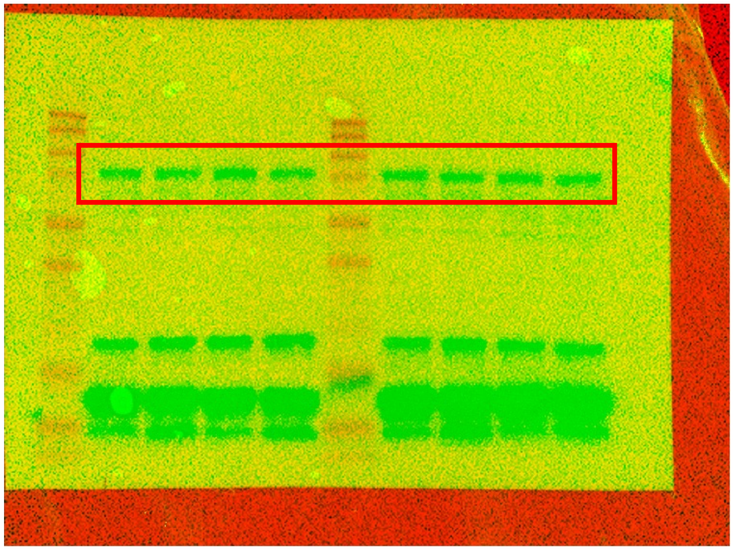

pDrp1 2

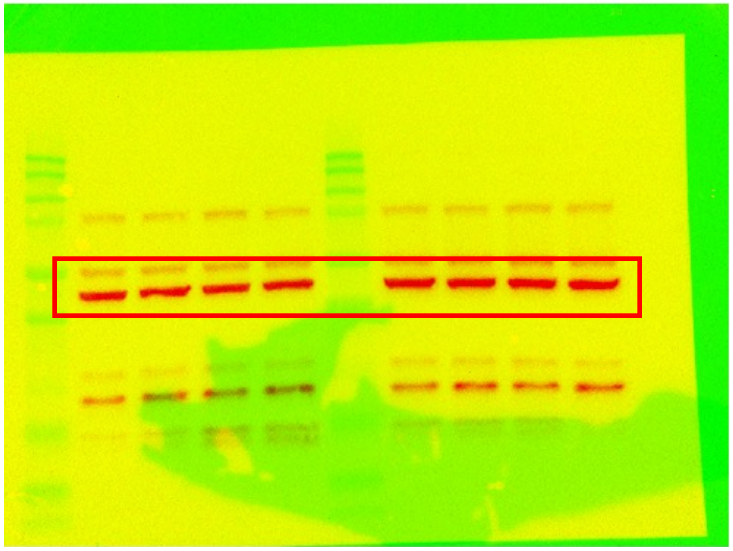

β-actin 2

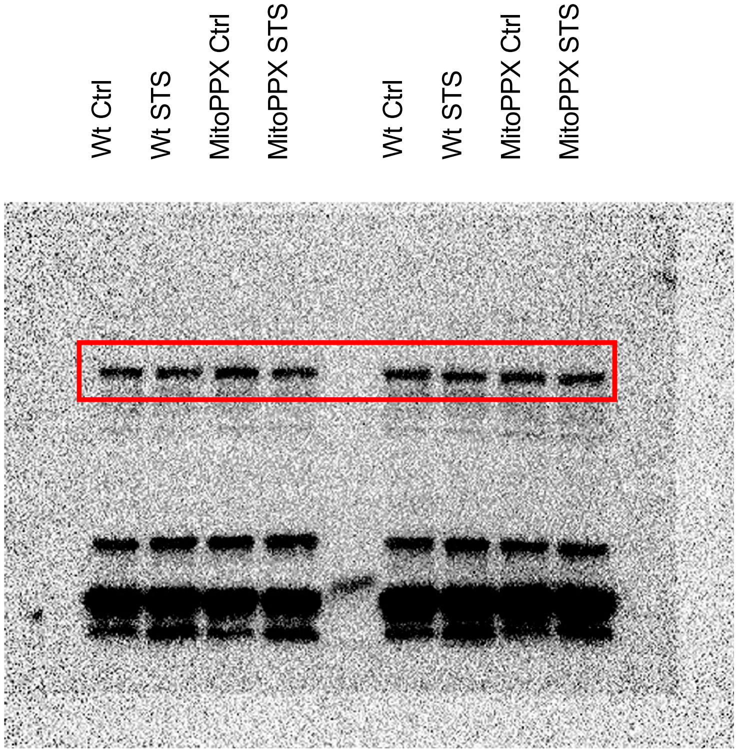

pDrp1 2

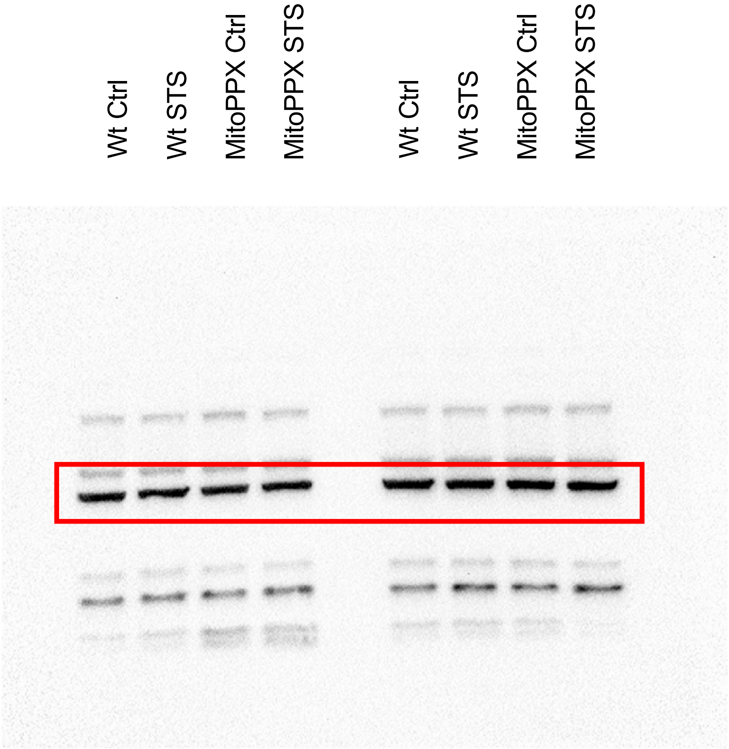

β-actin 2

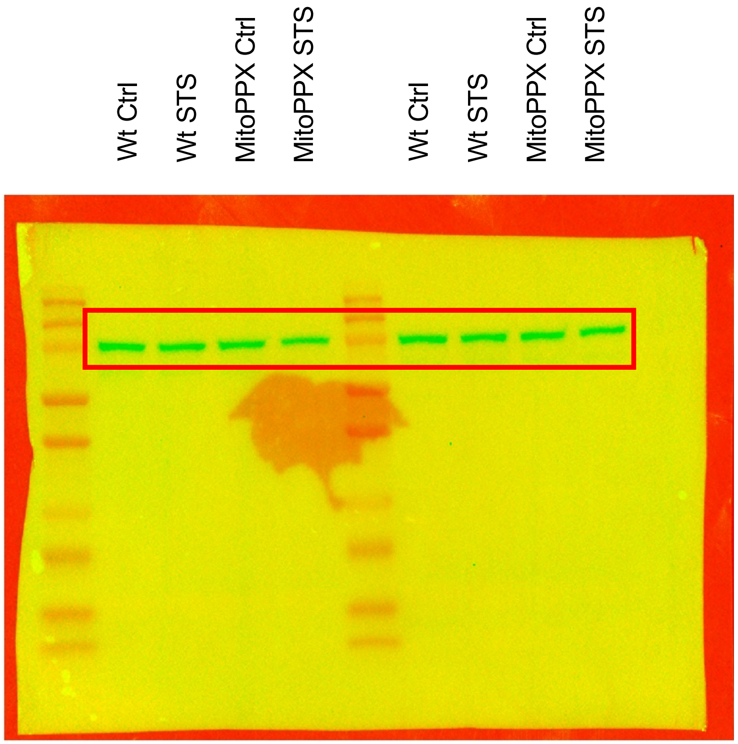

Mfn2 1

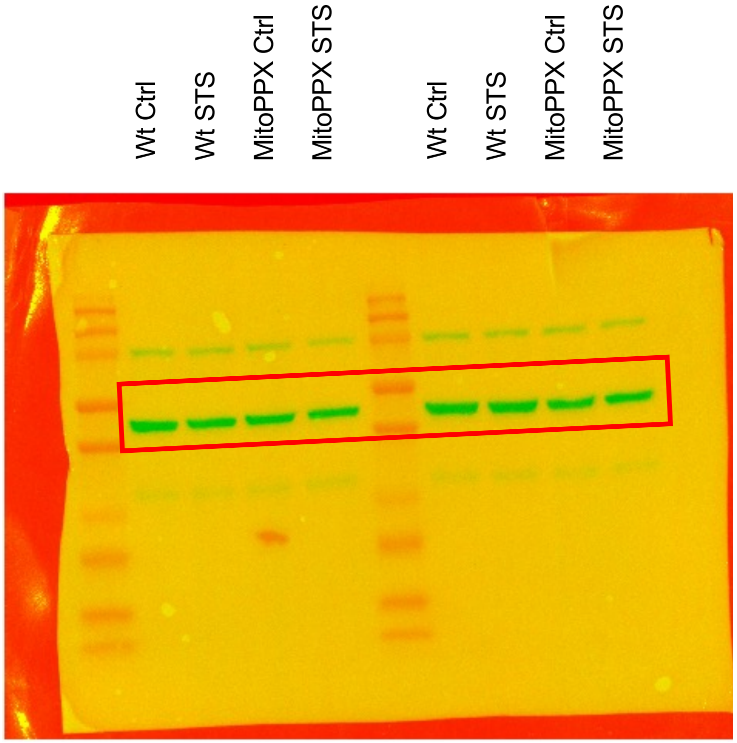

β-actin 1

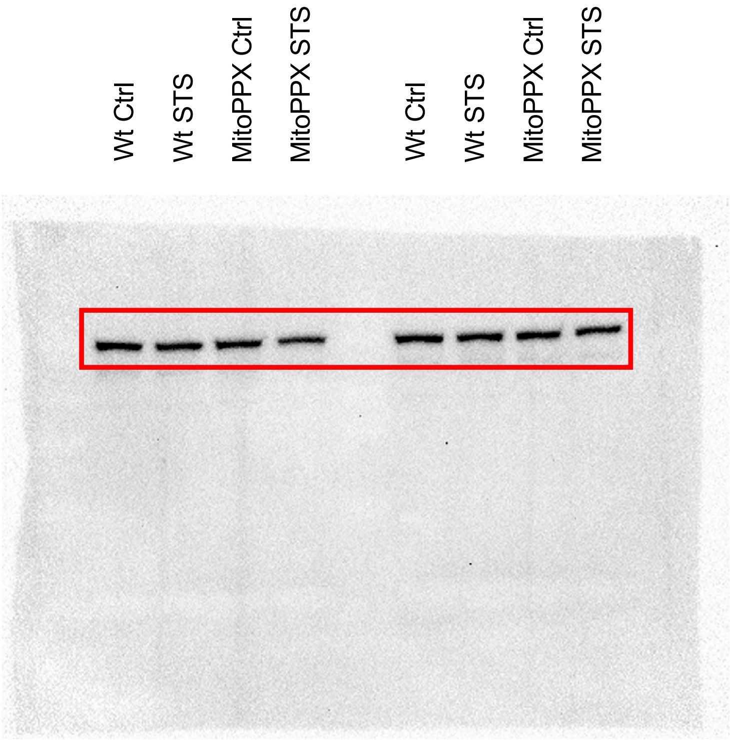

Mfn2 1

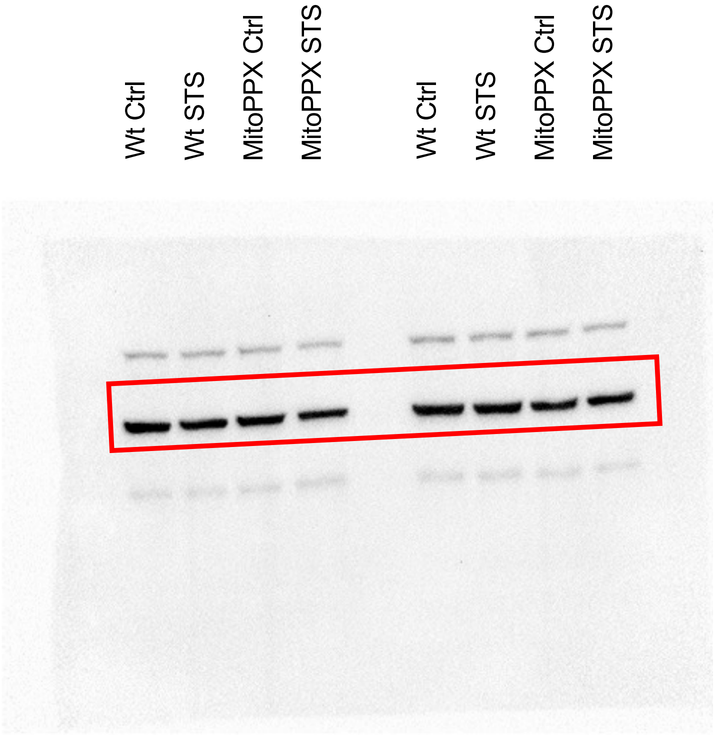

β-actin 1

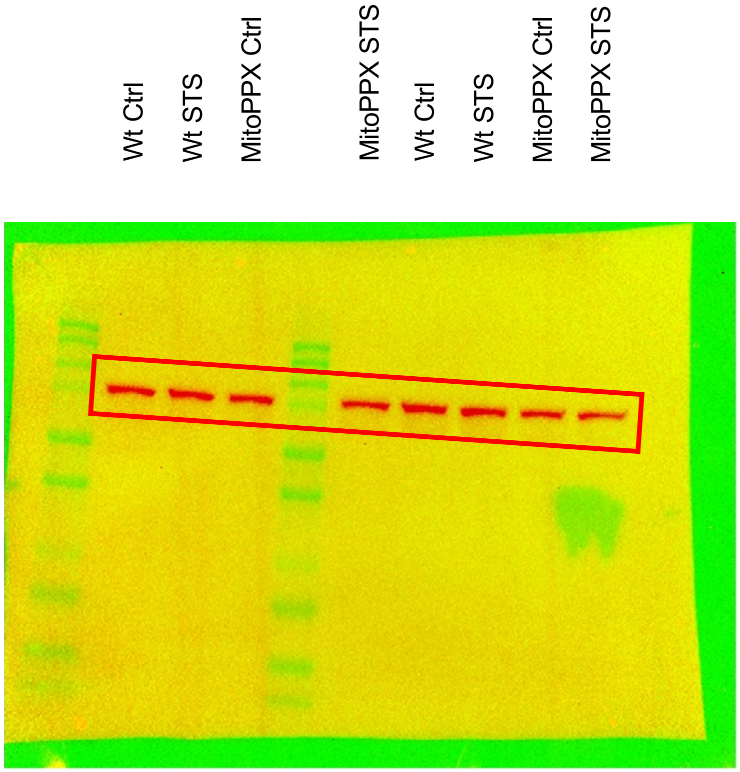

Mfn2 2

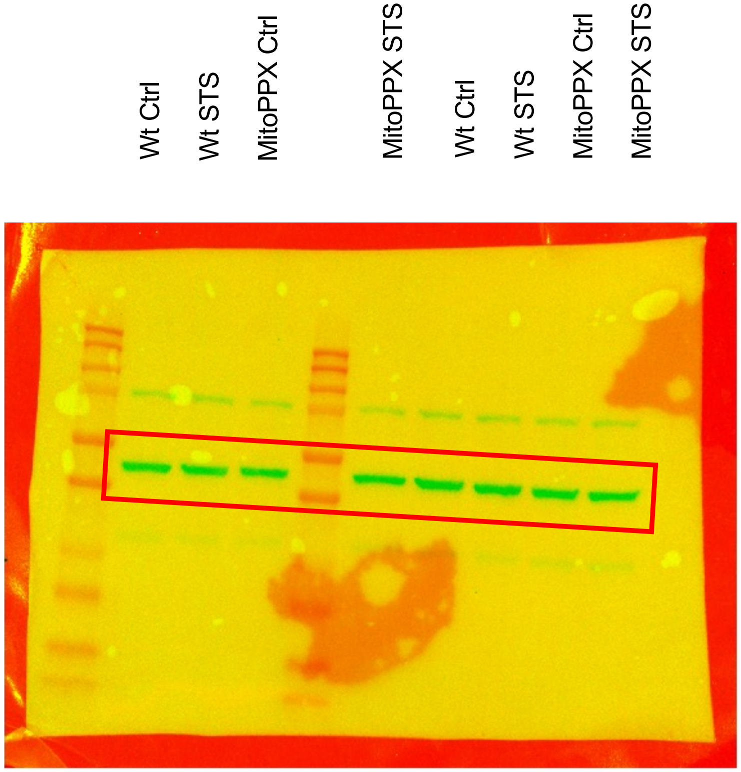

β-actin 2

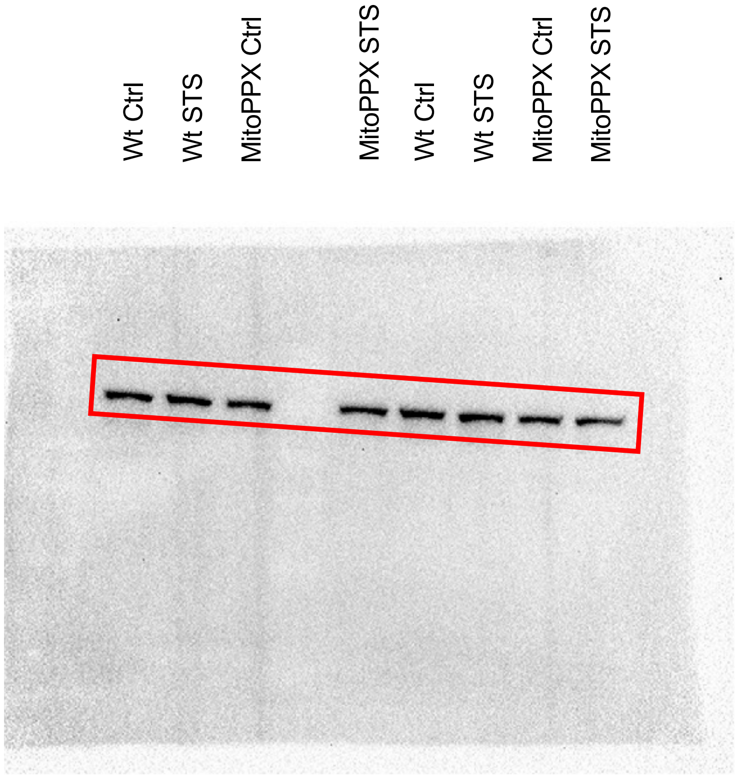

Mfn2 2

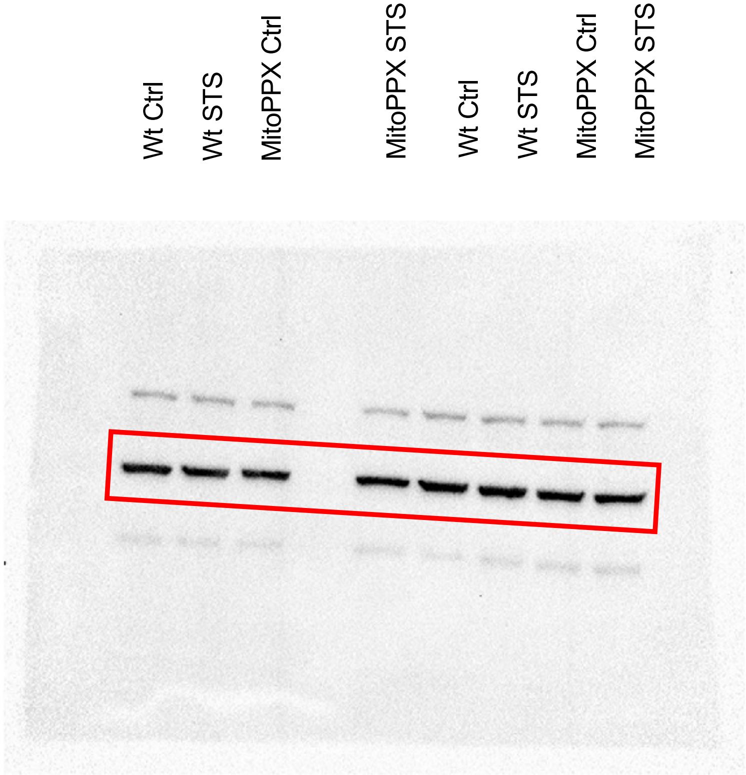

β-actin 2

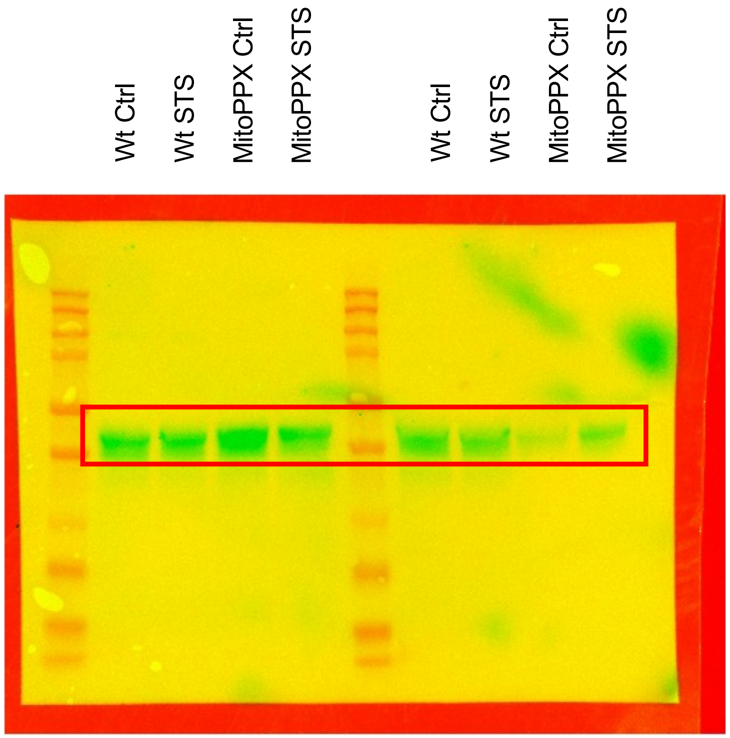

Parkin 1

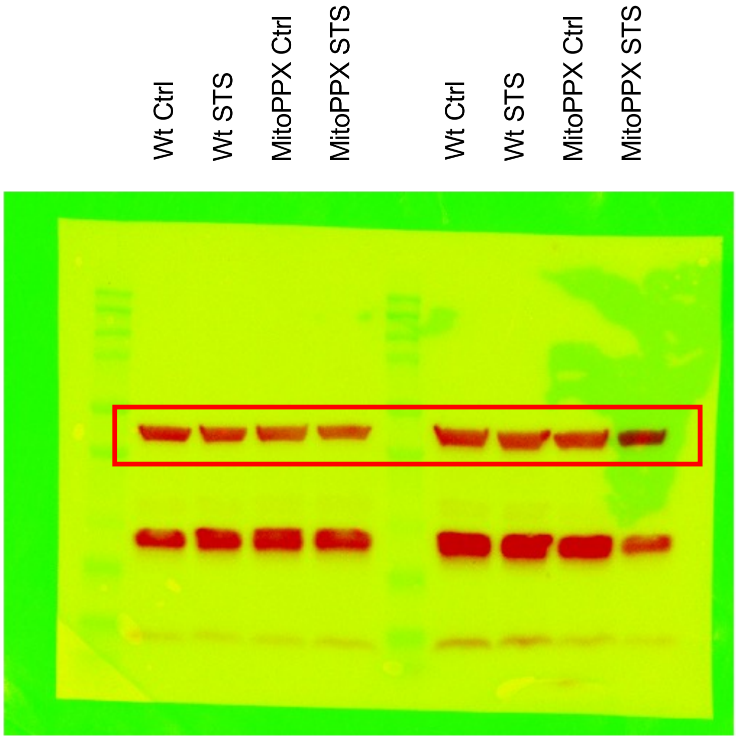

β-actin 1

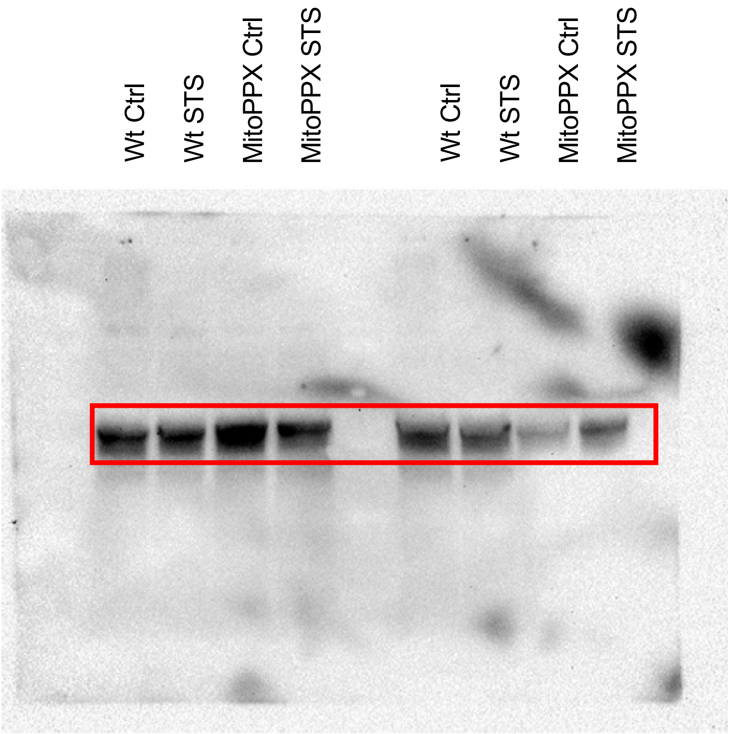

Parkin 1

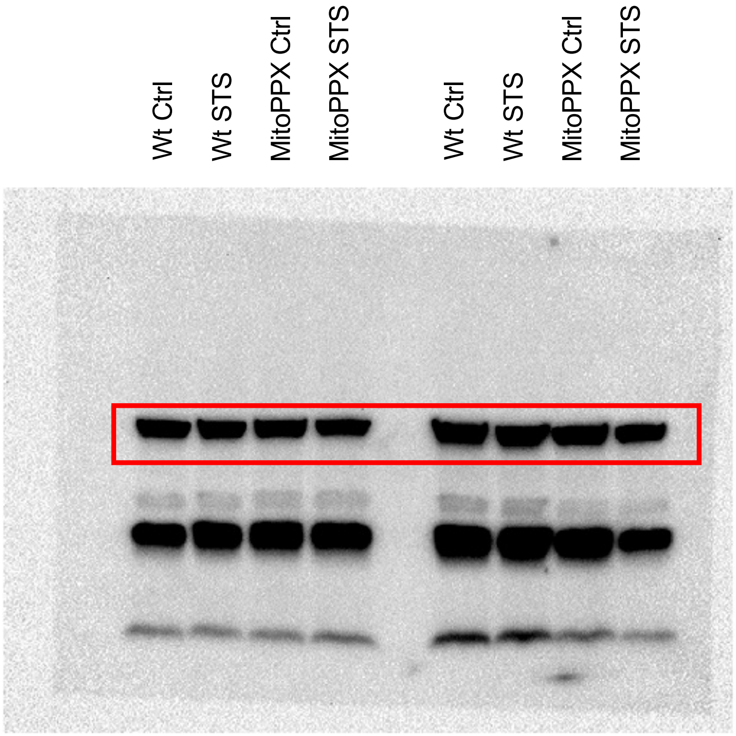

β-actin 1

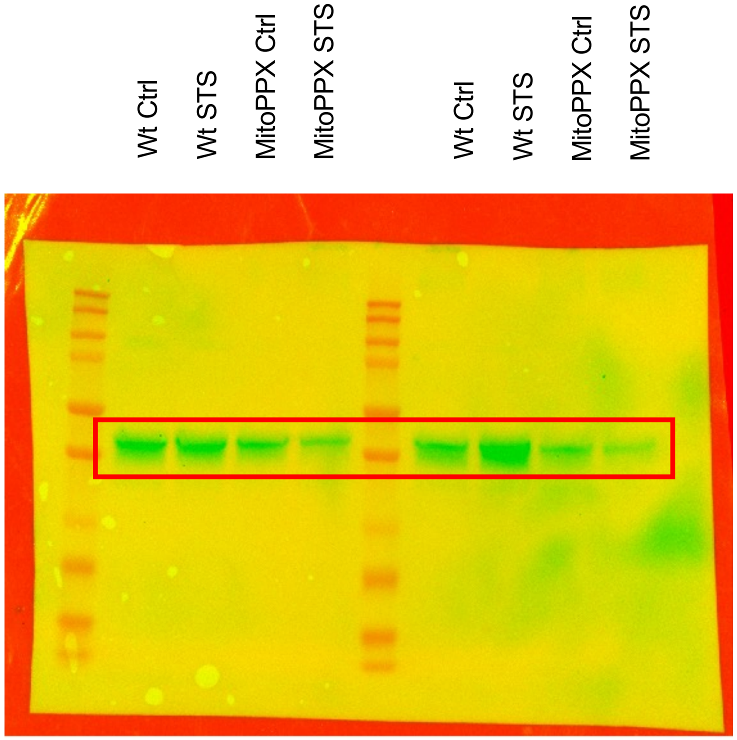

Parkin 2

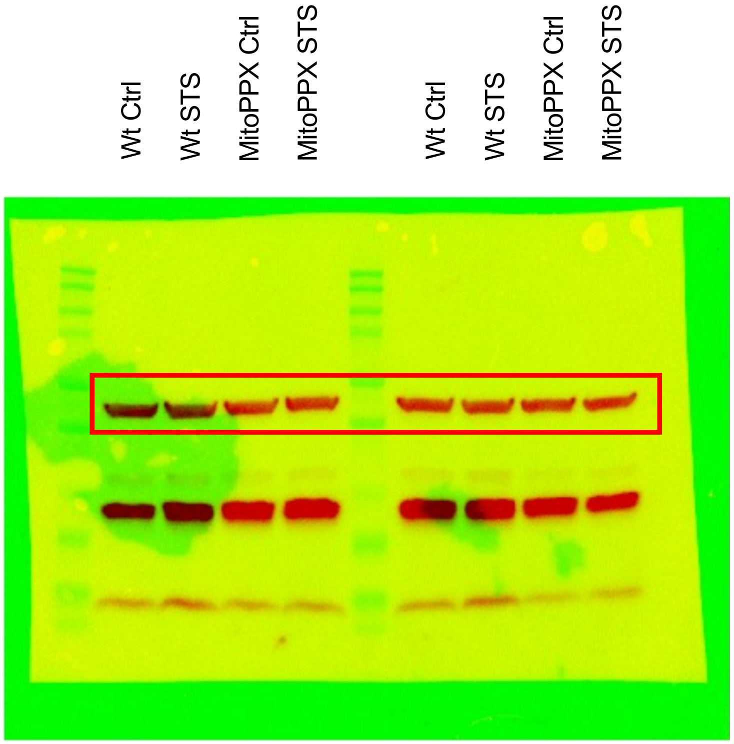

β-actin 2

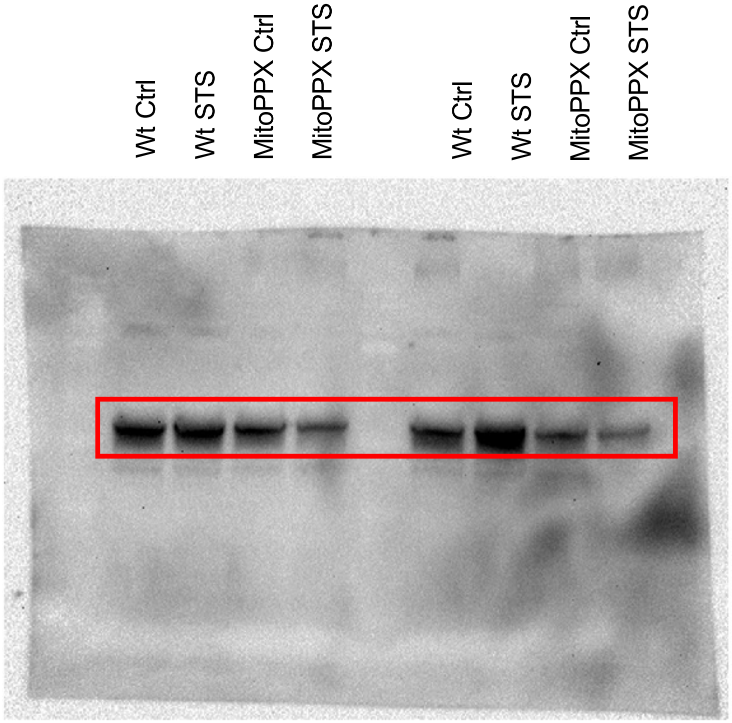

Parkin 2

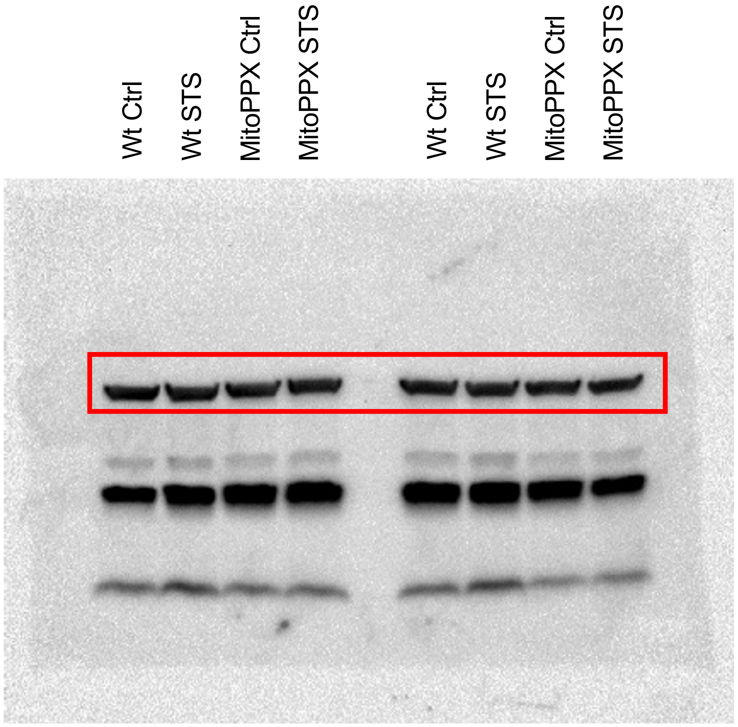

β-actin 2

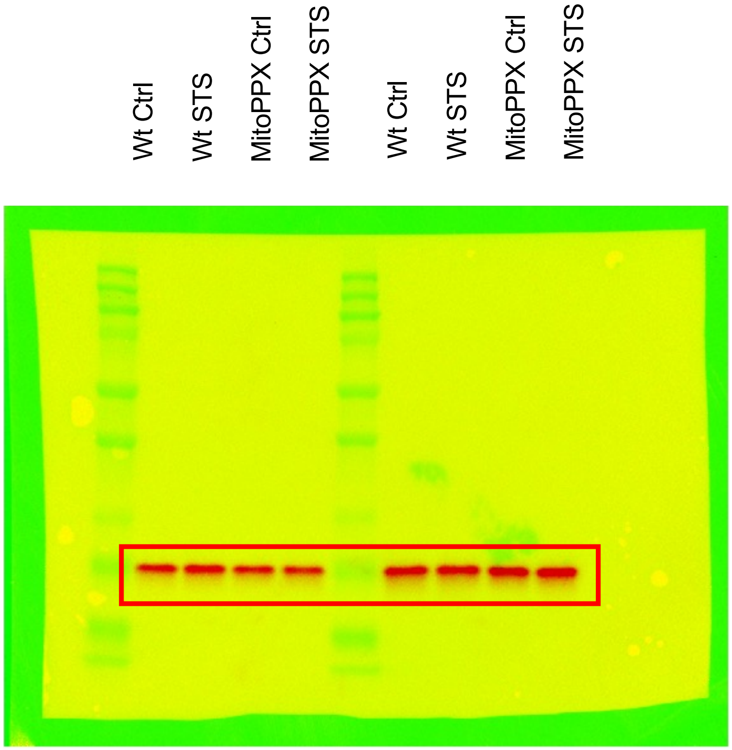
Wt Ctrl
Wt STS
MitoPPX Ctrl
MitoPPX STS
Wt Ctrl
Wt STS
MitoPPX Ctrl
MitoPPX STS

BAX 1

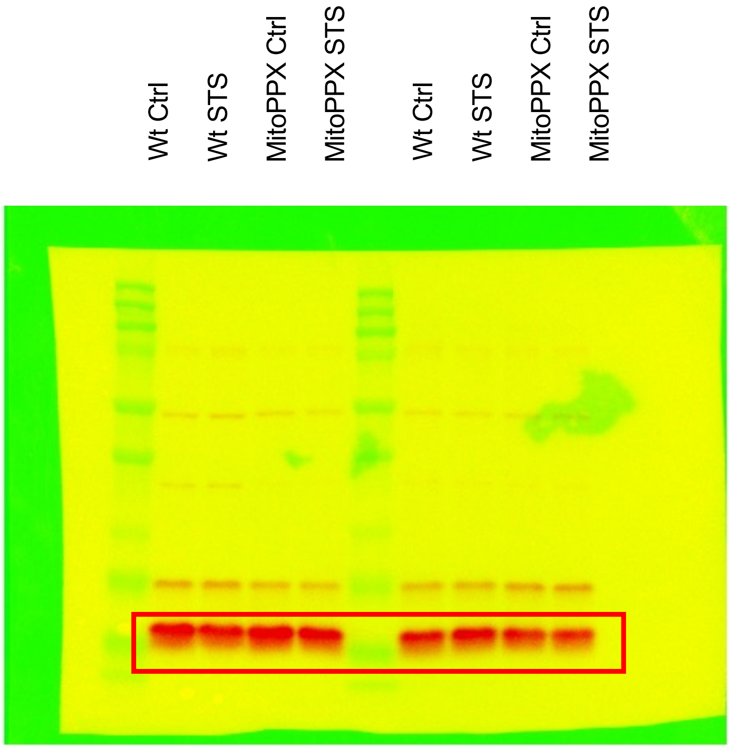
Wt Ctrl
Wt STS
MitoPPX Ctrl
MitoPPX STS
Wt Ctrl
Wt STS
MitoPPX Ctrl
MitoPPX STS

TOM20 1

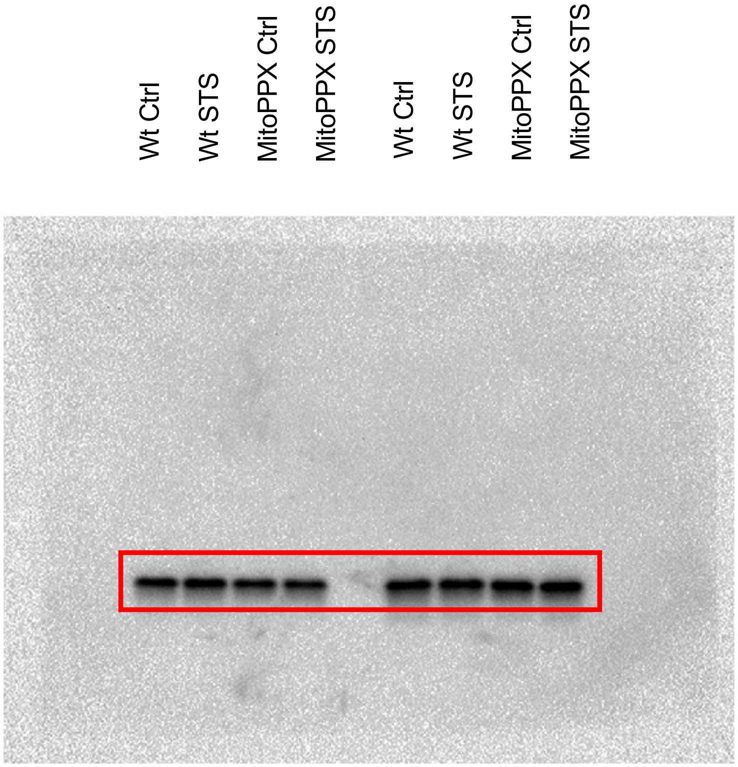
Wt Ctrl
Wt STS
MitoPPX Ctrl
MitoPPX STS
Wt Ctrl
Wt STS
MitoPPX Ctrl
MitoPPX STS

BAX 1

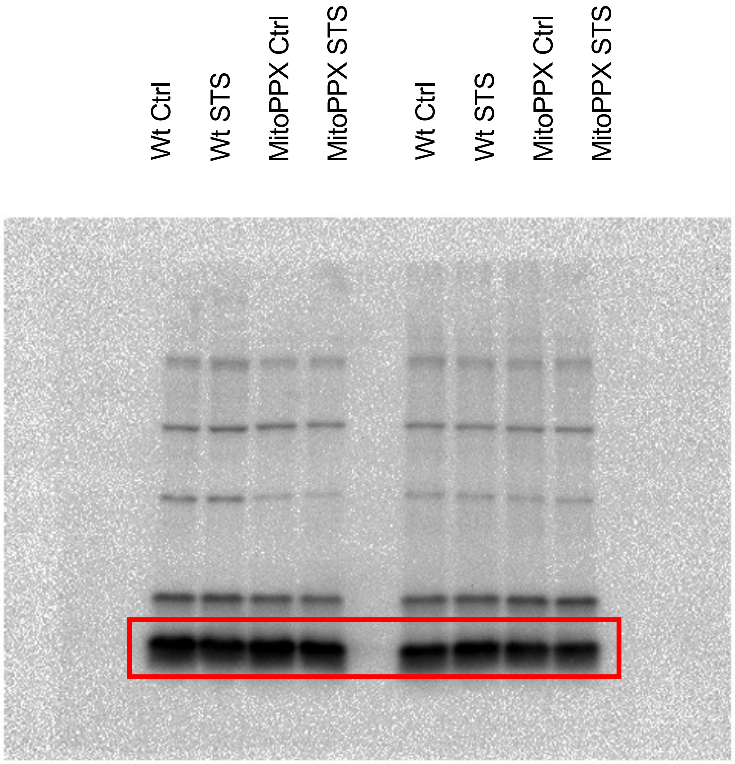
Wt Ctrl
Wt STS
MitoPPX Ctrl
MitoPPX STS
Wt Ctrl
Wt STS
MitoPPX Ctrl
MitoPPX STS

TOM20 1

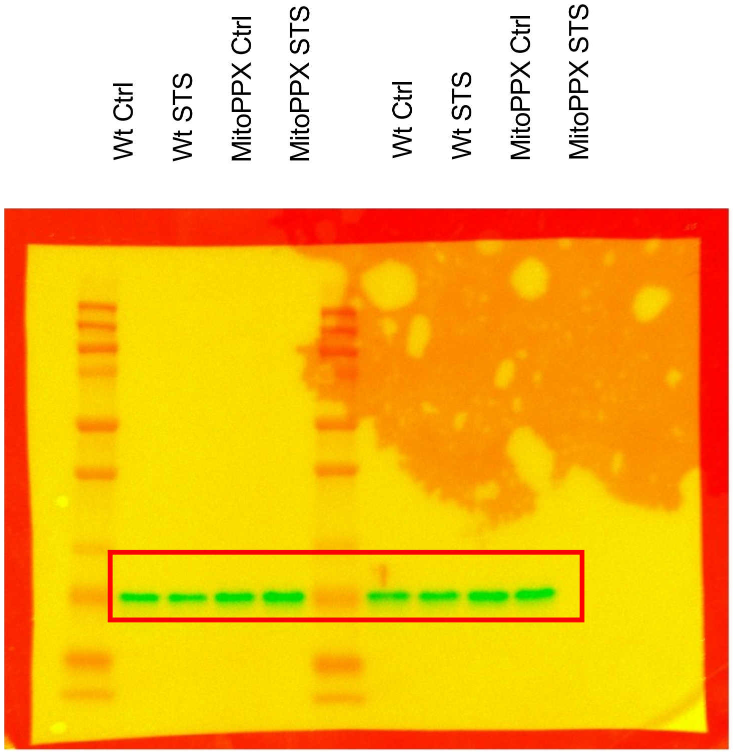
Wt Ctrl
Wt STS
MitoPPX Ctrl
MitoPPX STS
Wt Ctrl
Wt STS
MitoPPX Ctrl
MitoPPX STS

BAX 2

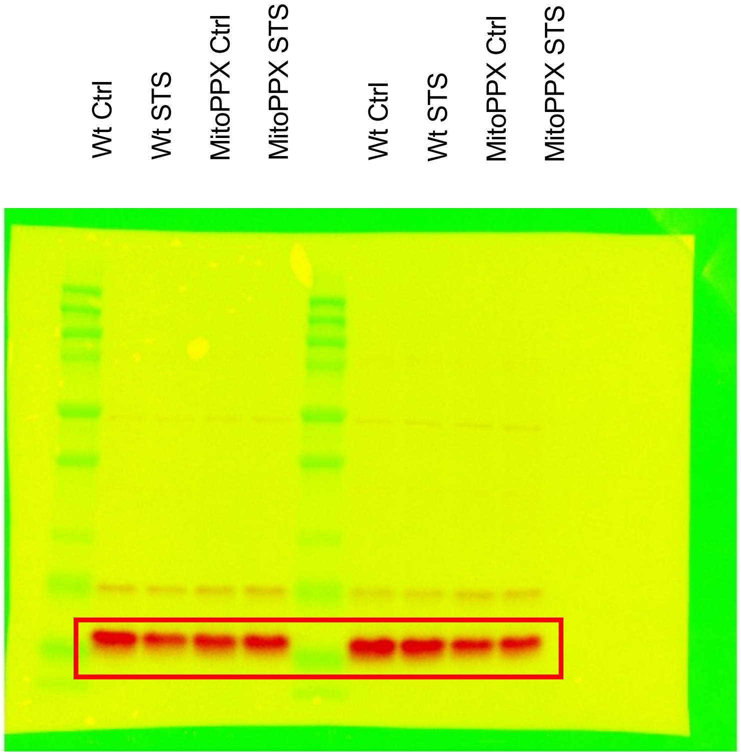
Wt Ctrl
Wt STS
MitoPPX Ctrl
MitoPPX STS
Wt Ctrl
Wt STS
MitoPPX Ctrl
MitoPPX STS

TOM20 2

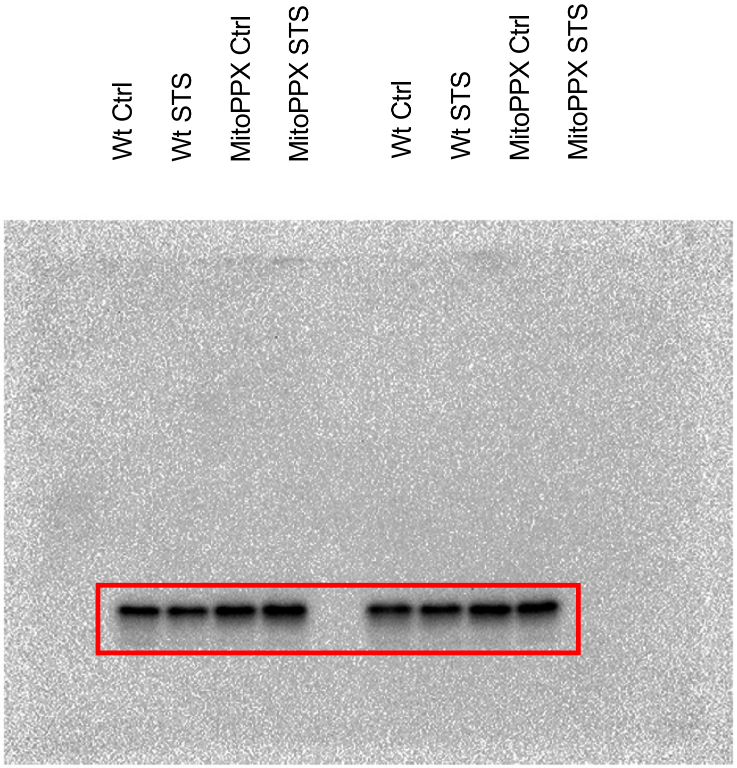
Wt Ctrl
Wt STS
MitoPPX Ctrl
MitoPPX STS
Wt Ctrl
Wt STS
MitoPPX Ctrl
MitoPPX STS

BAX 2

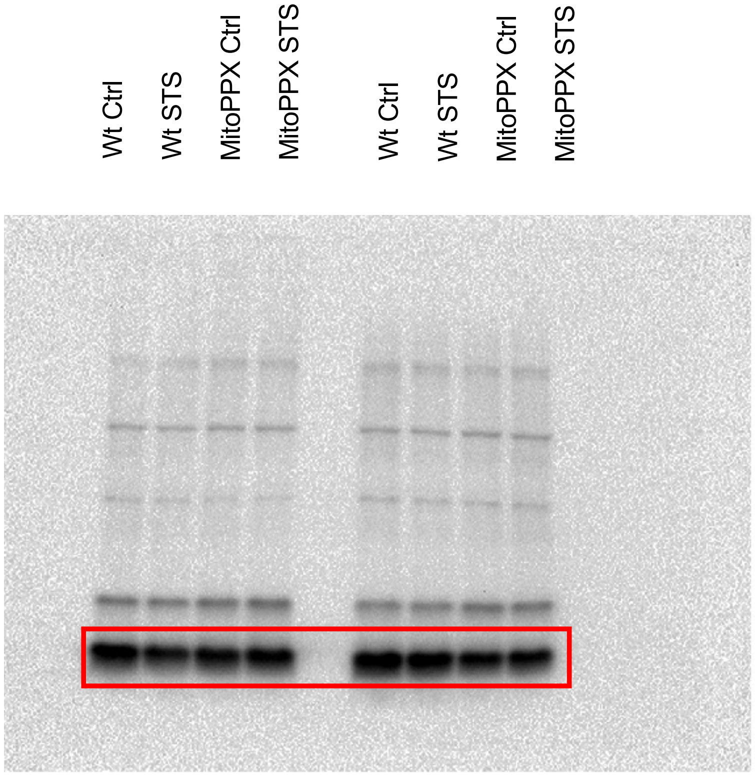
Wt Ctrl
Wt STS
MitoPPX Ctrl
MitoPPX STS
Wt Ctrl
Wt STS
MitoPPX Ctrl
MitoPPX STS

TOM20 2

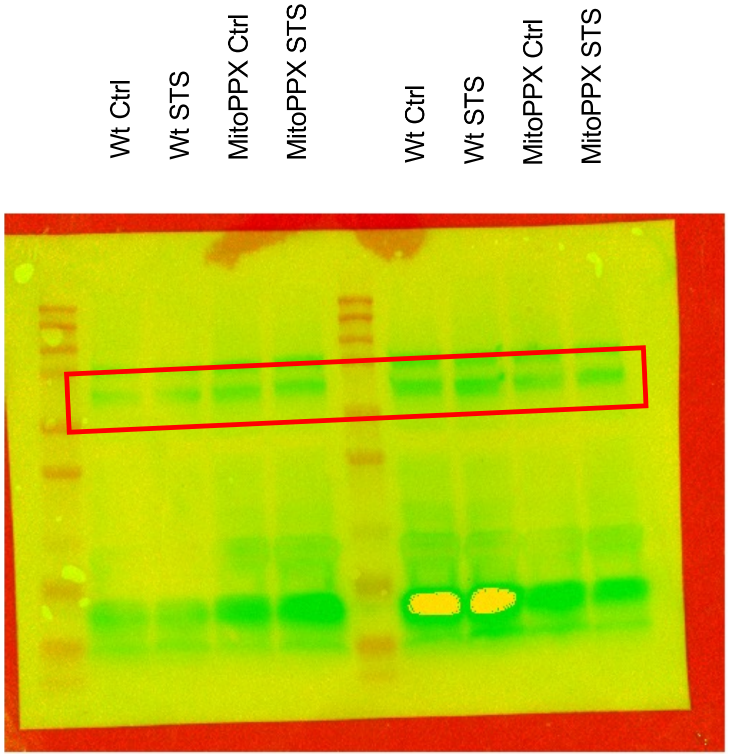

pAMPK 1

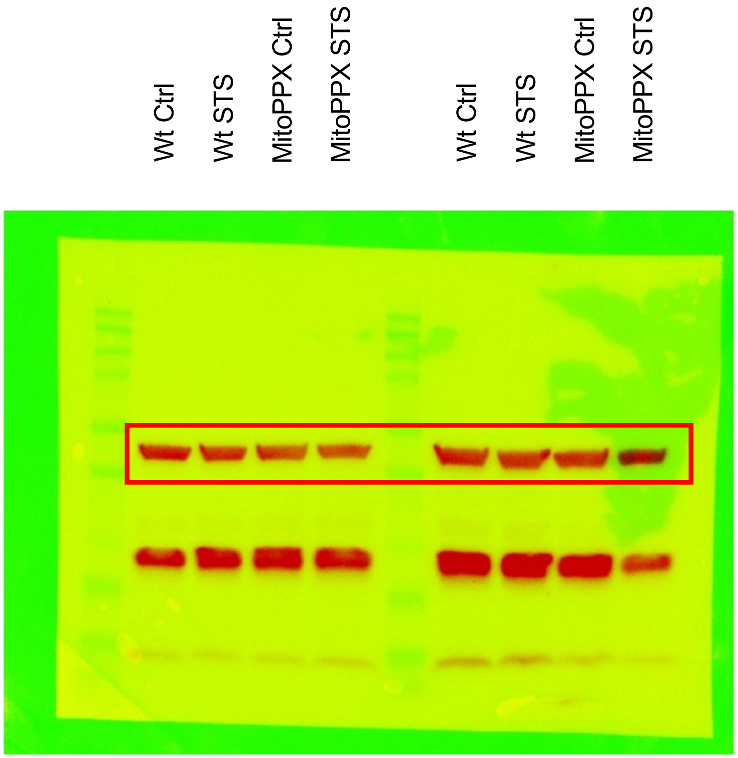

β-actin 1

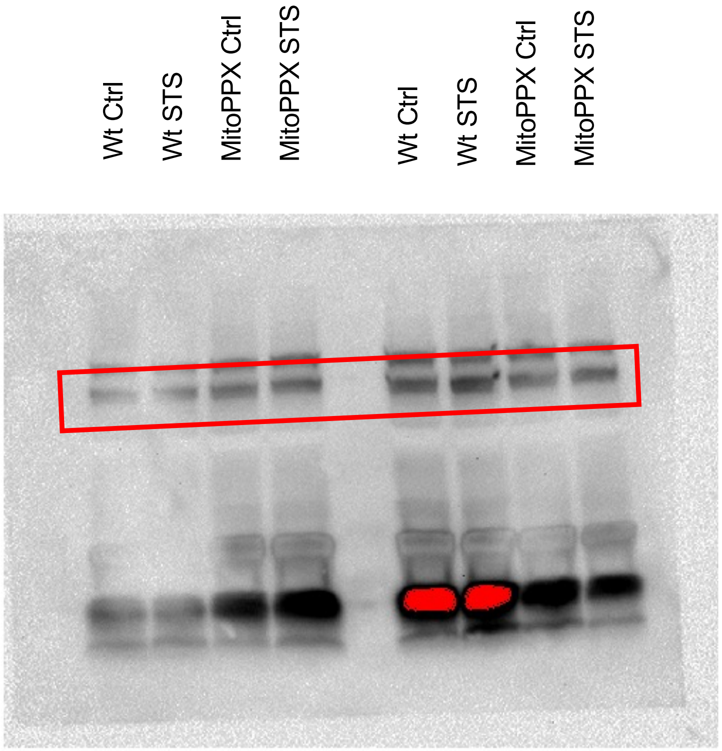

pAMPK 1

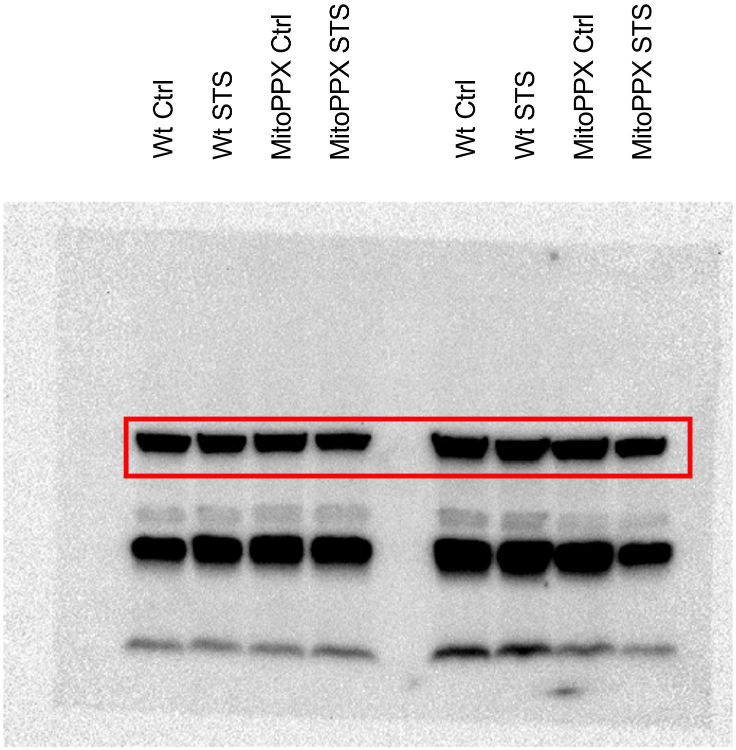

β-actin 1

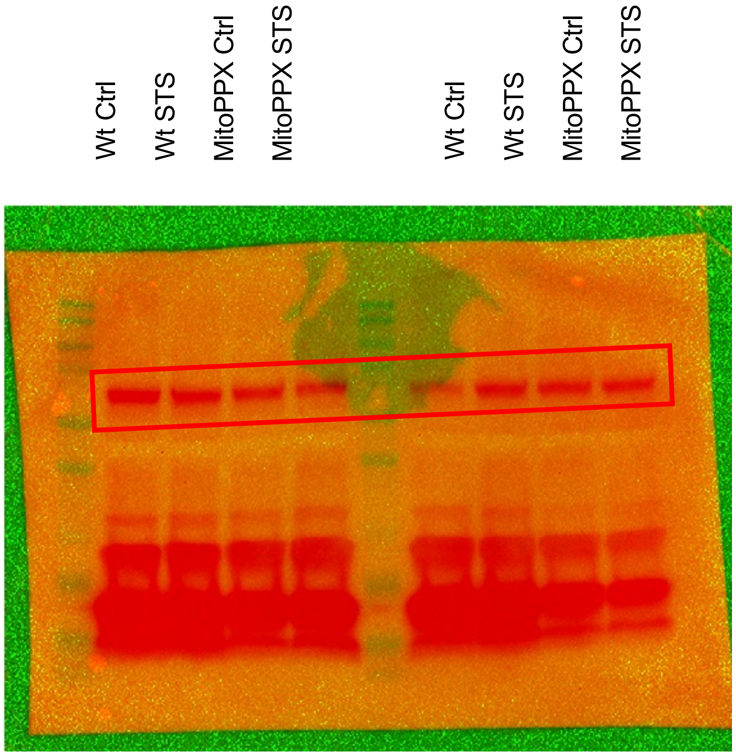

pAMPK 2

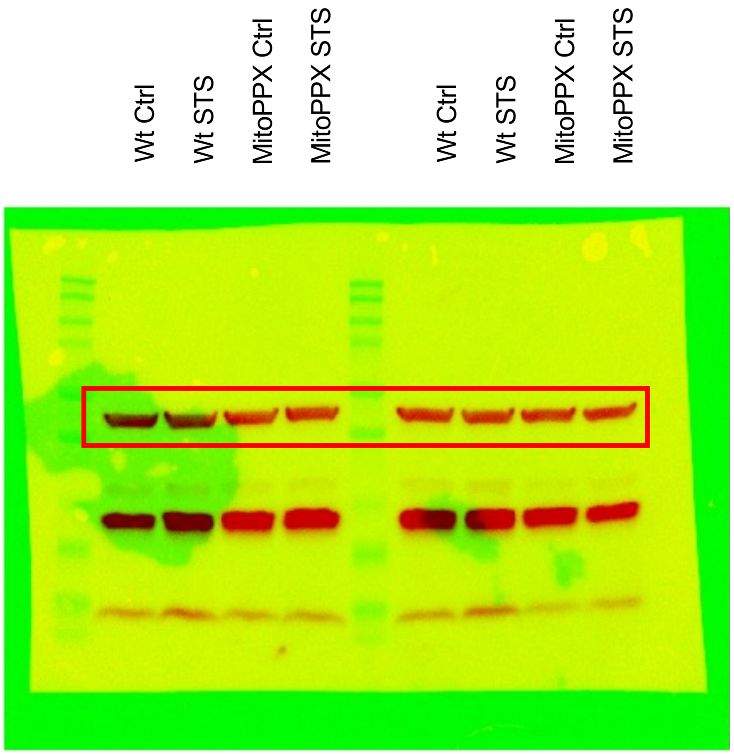

β-actin 2

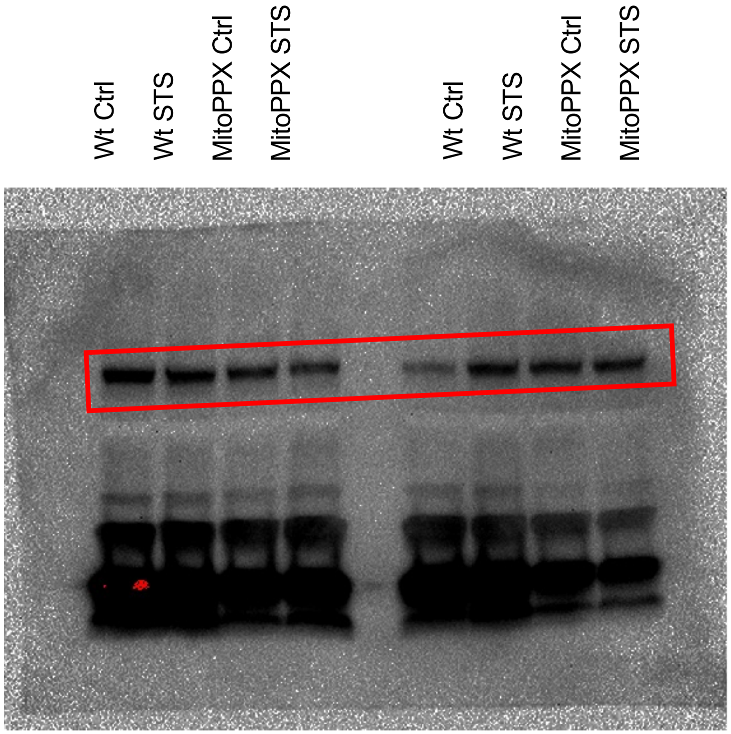

pAMPK 2

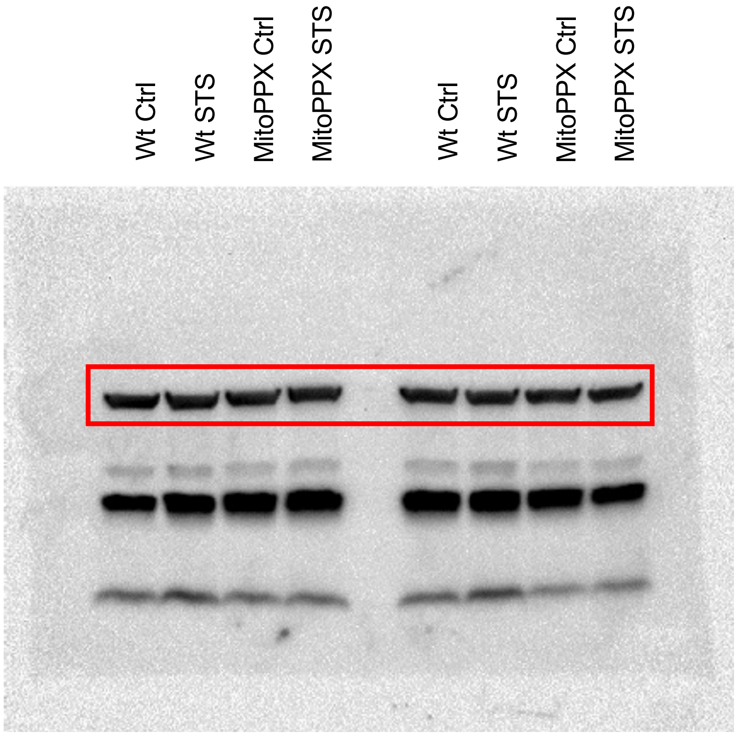

β-actin 2

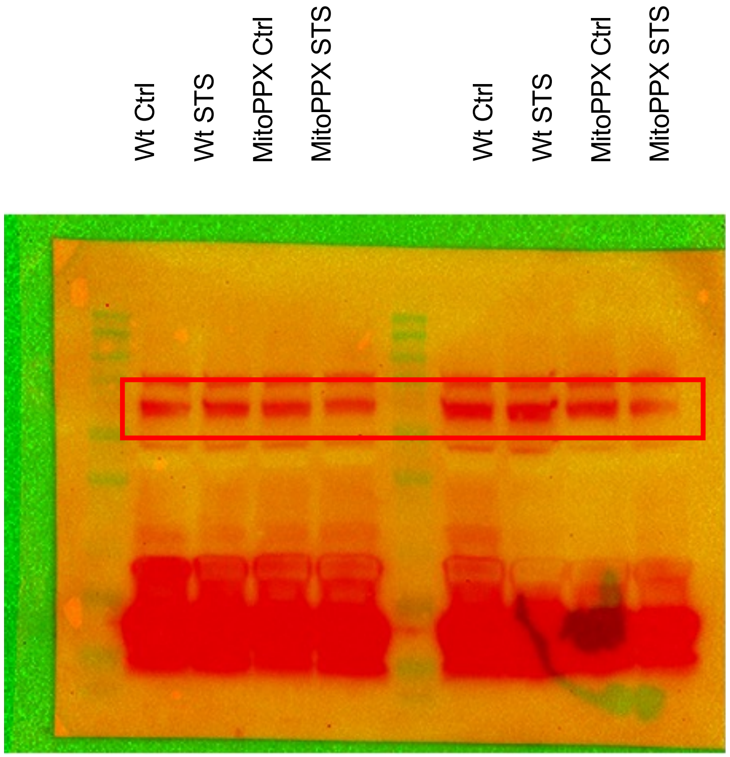

AMPK 1

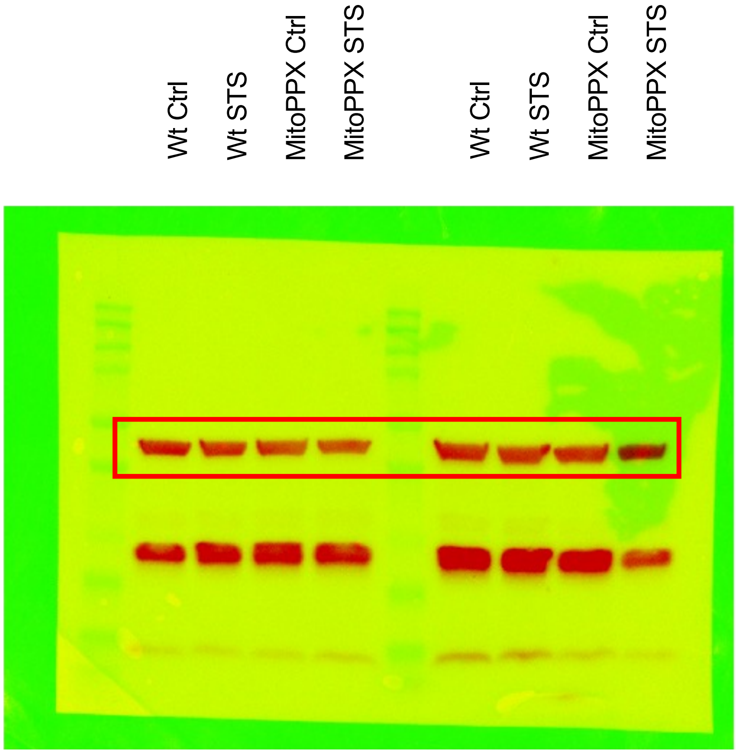

β-actin 1

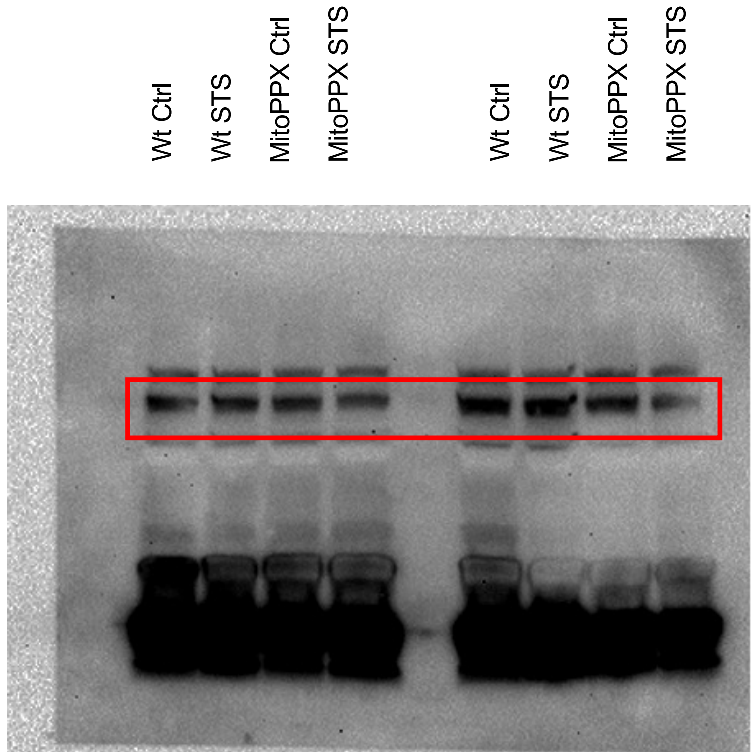

AMPK 1

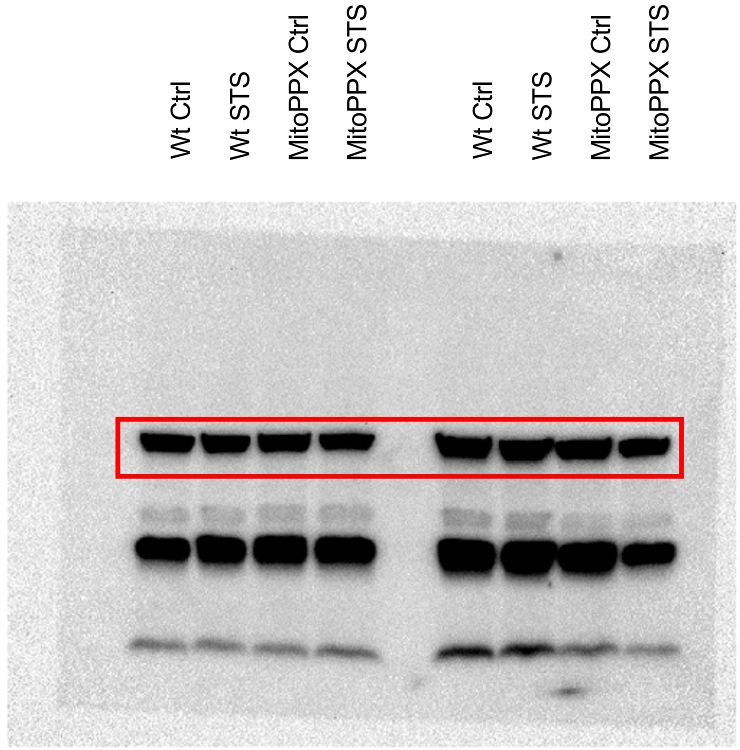

β-actin 1

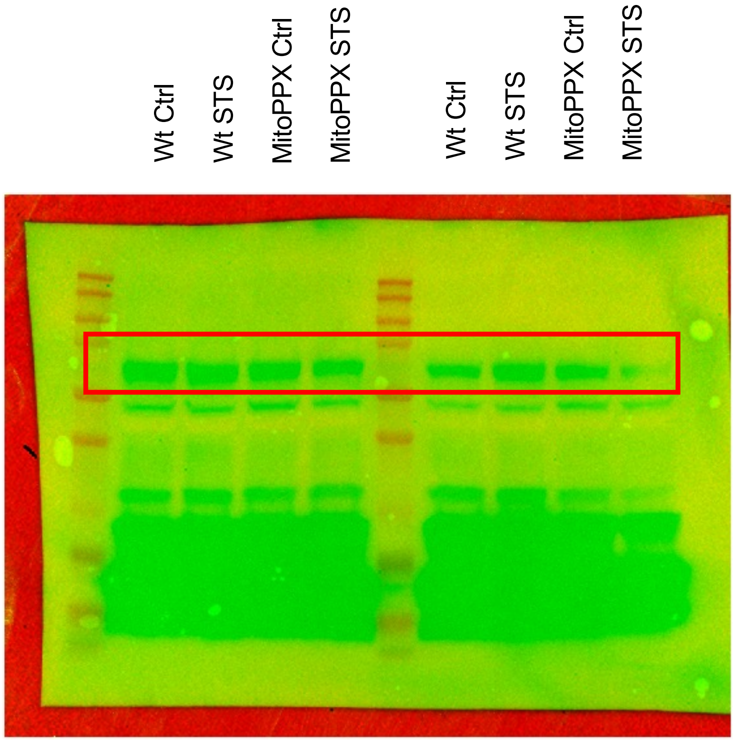

AMPK 2

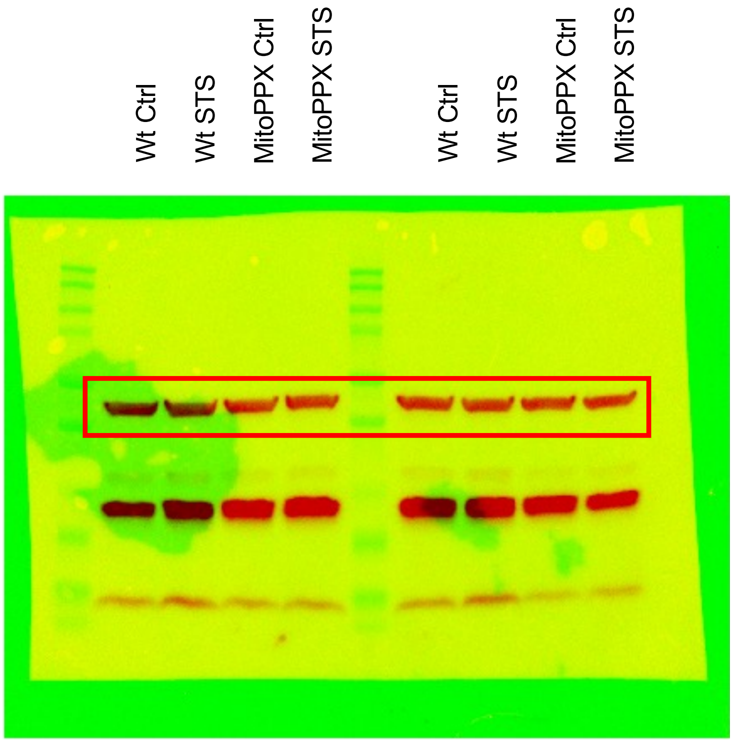

β-actin 2

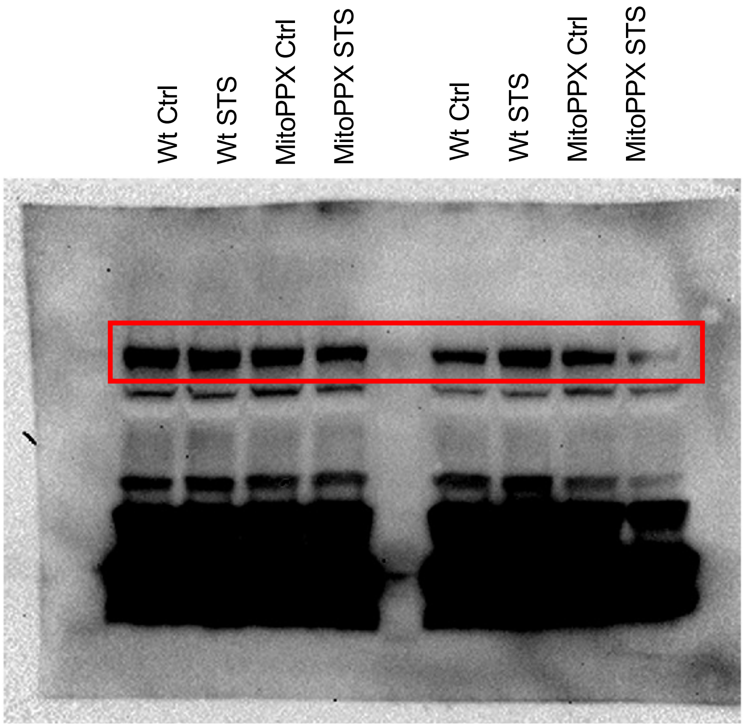

AMPK 2

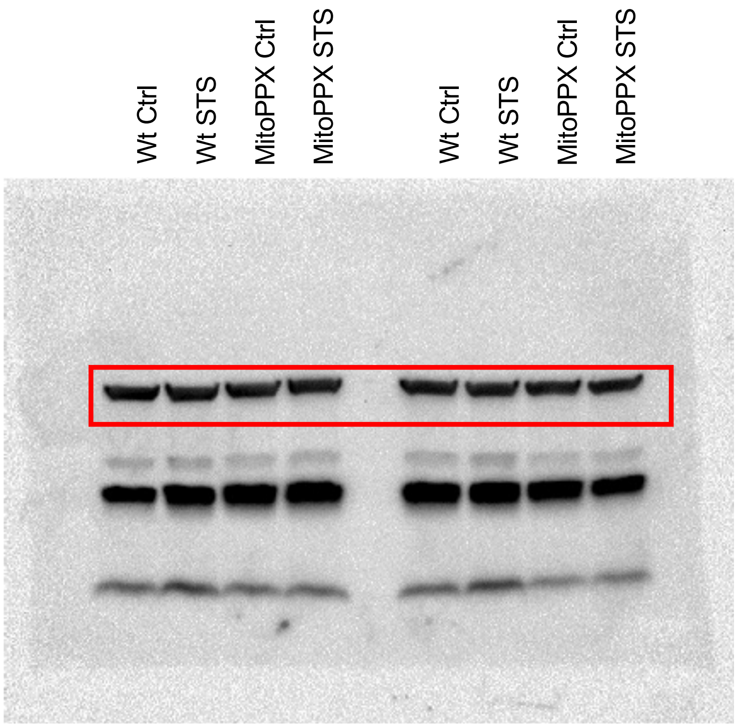

β-actin 2

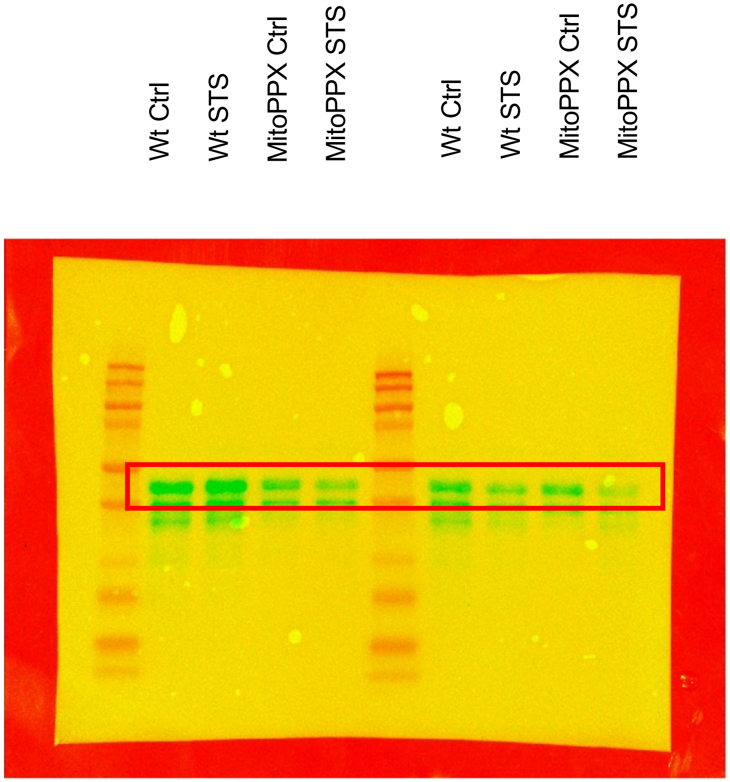

pCREB 1

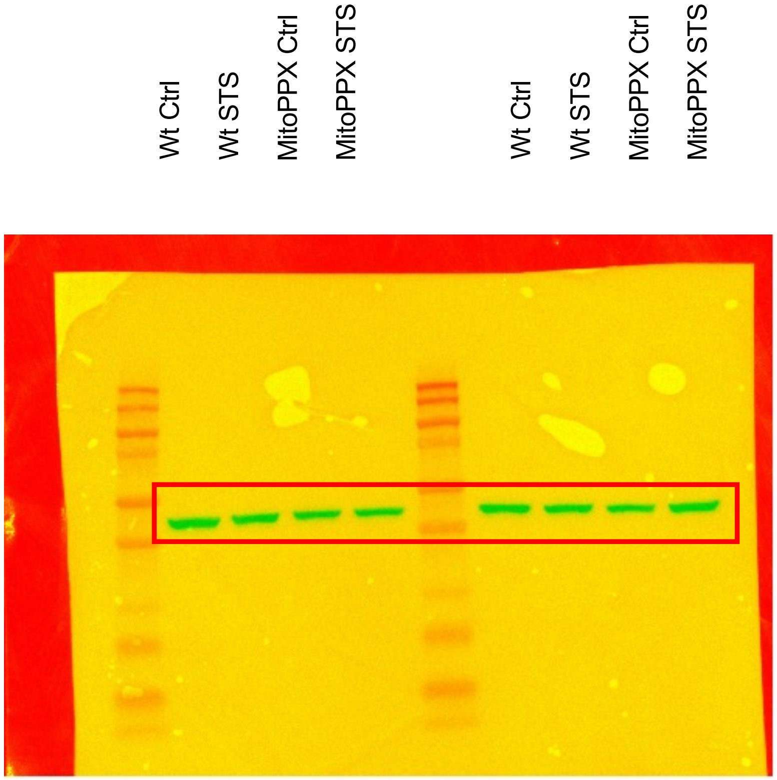

β-actin 1

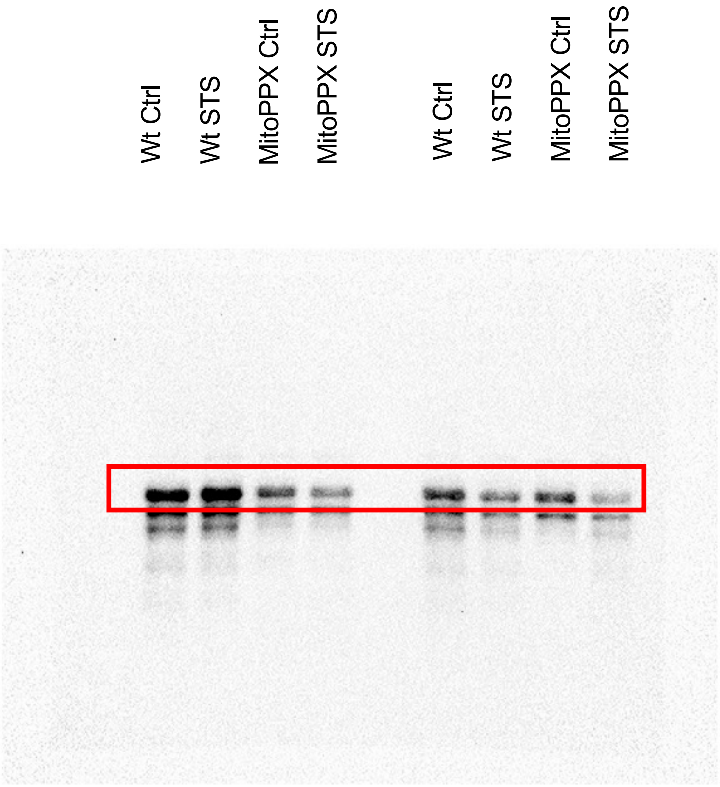

pCREB 1

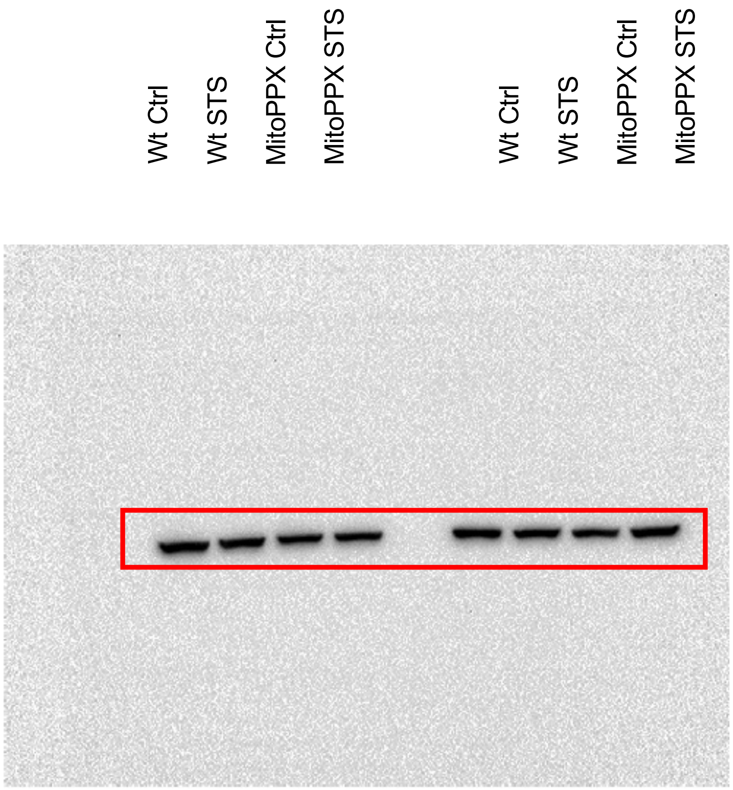

β-actin 1

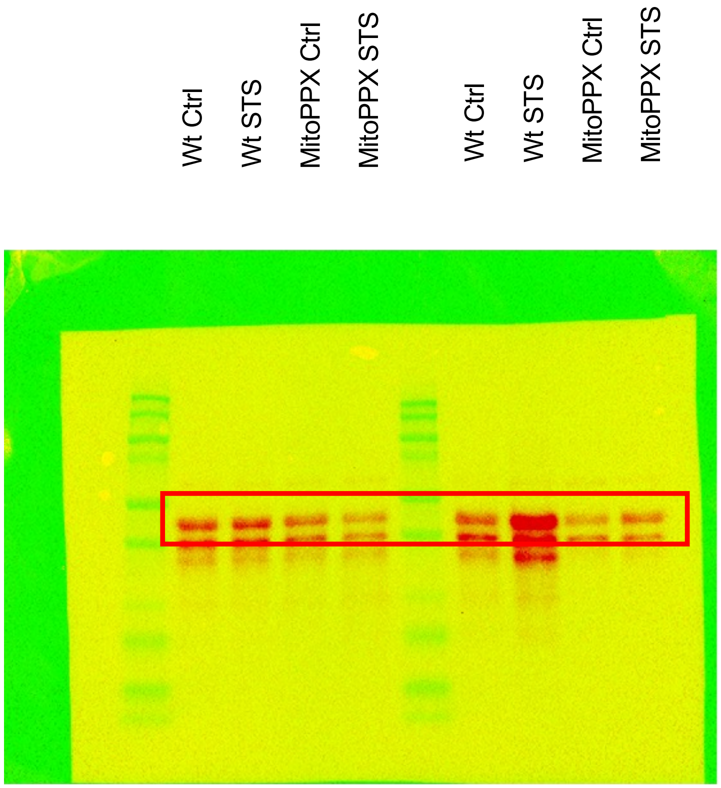

pCREB 2

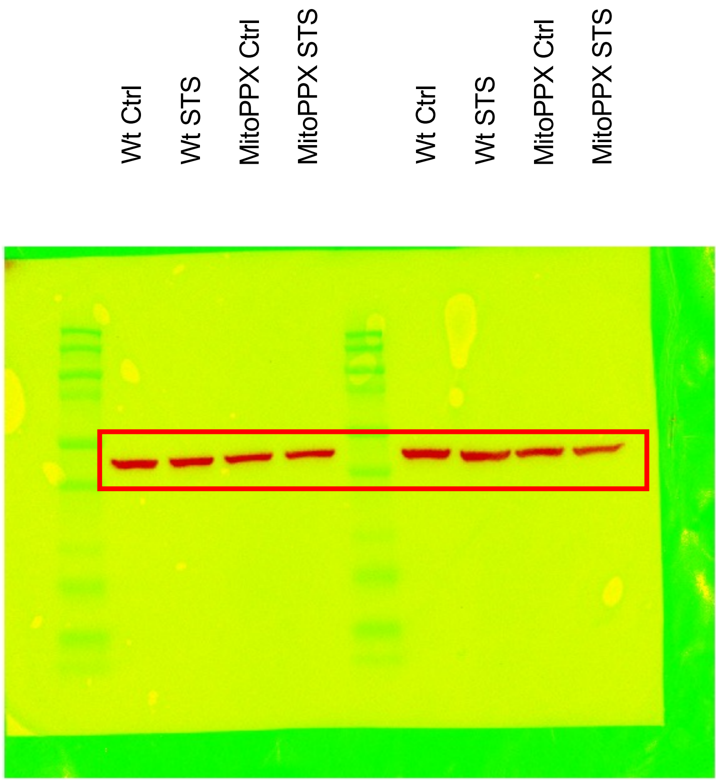

β-actin 2

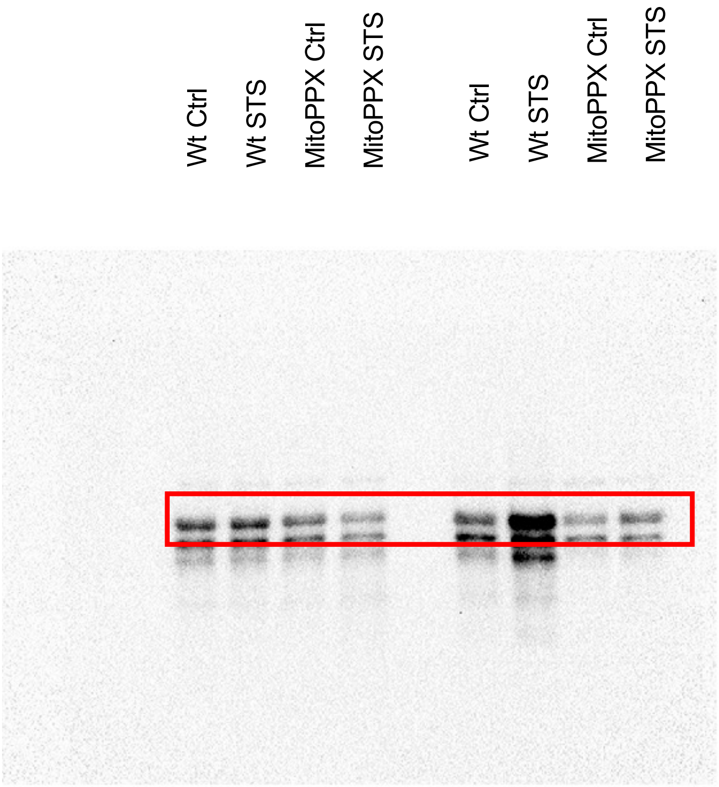

pCREB 2

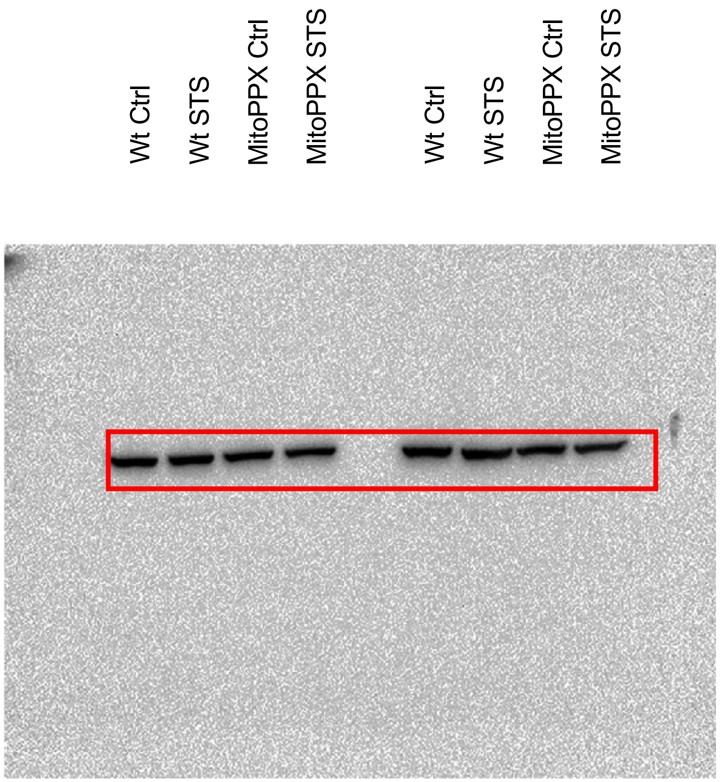

β-actin 2

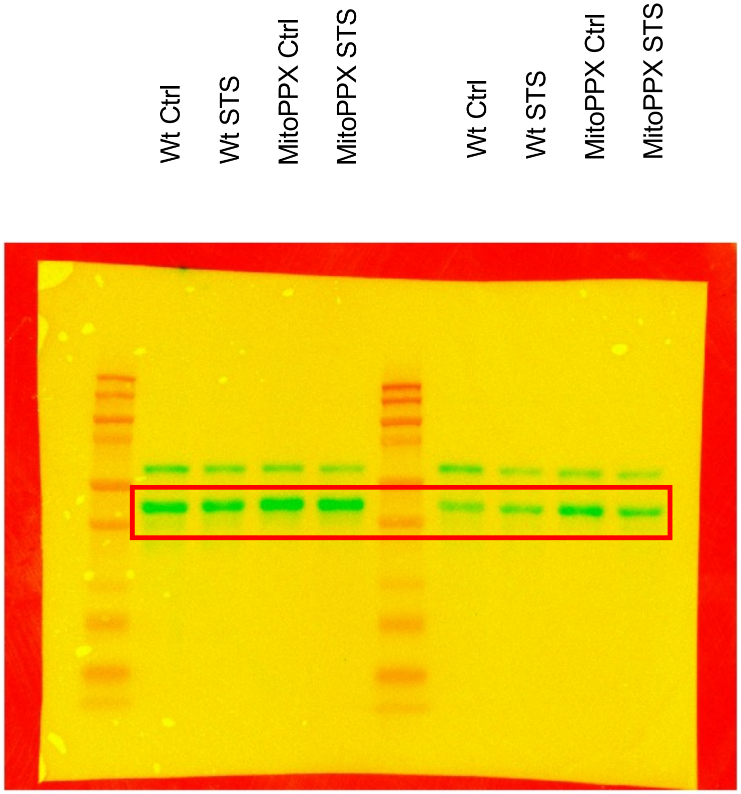

CREB 1

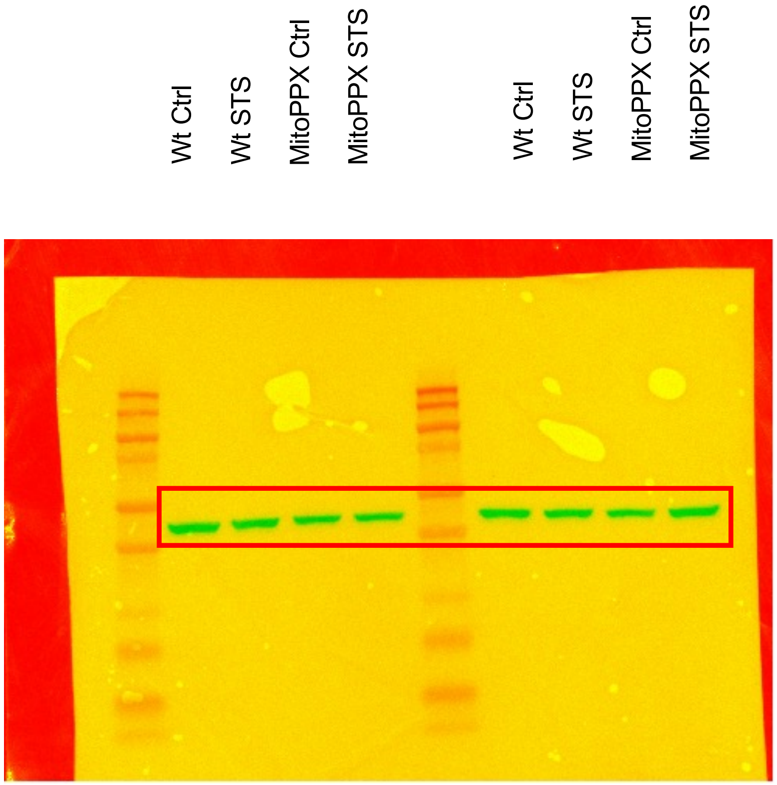

β-actin 1

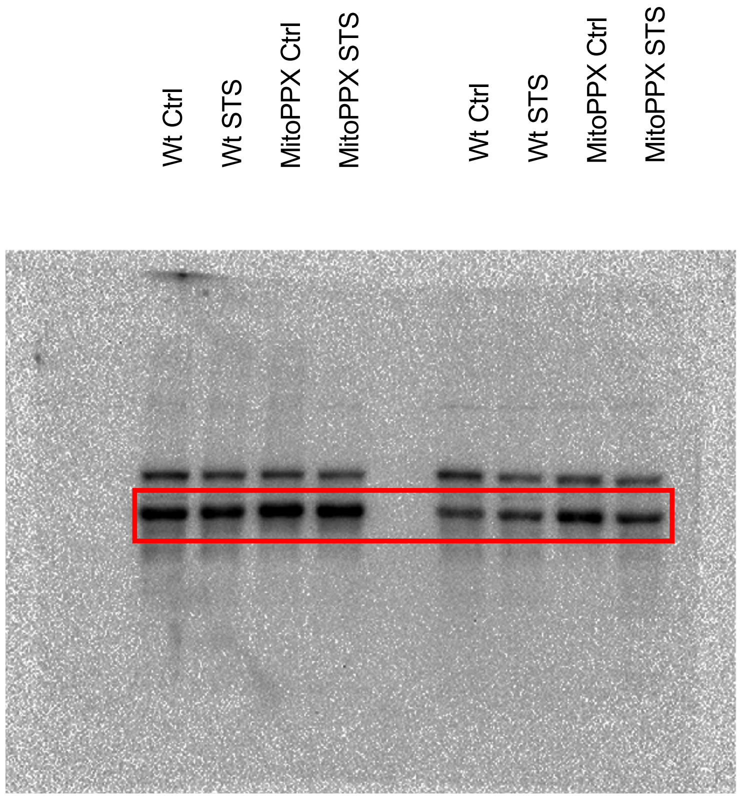

CREB 1

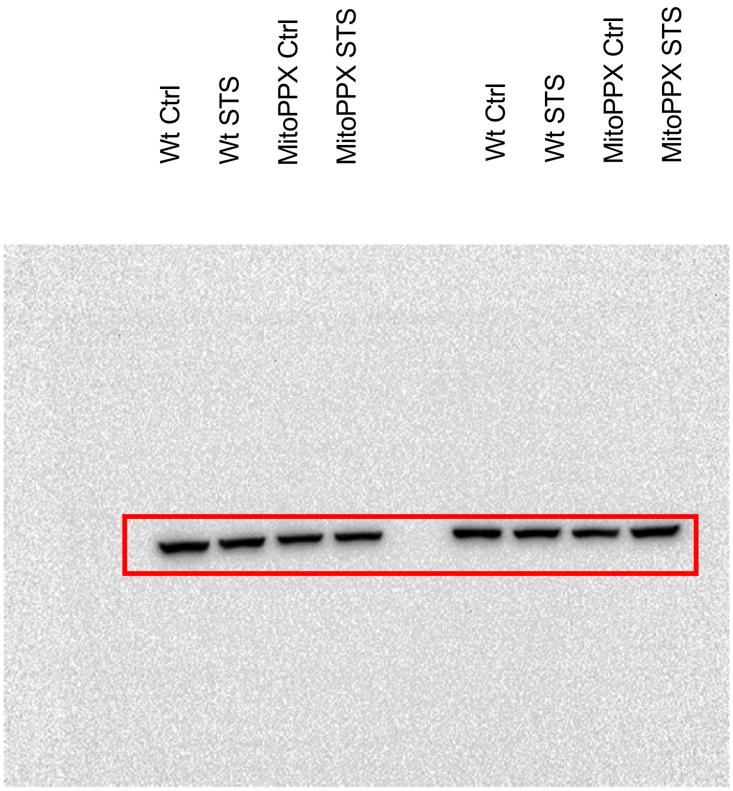

β-actin 1

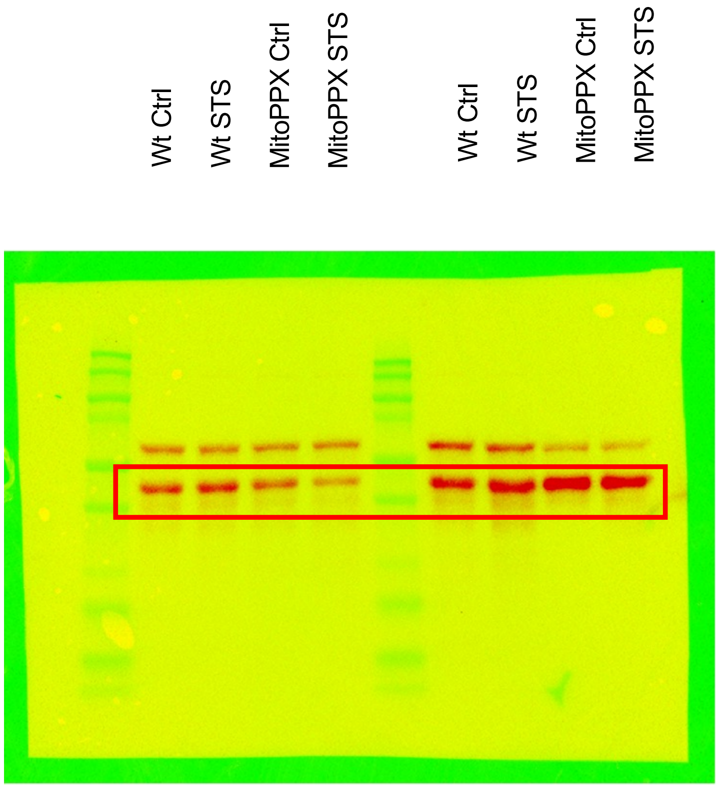

CREB 2

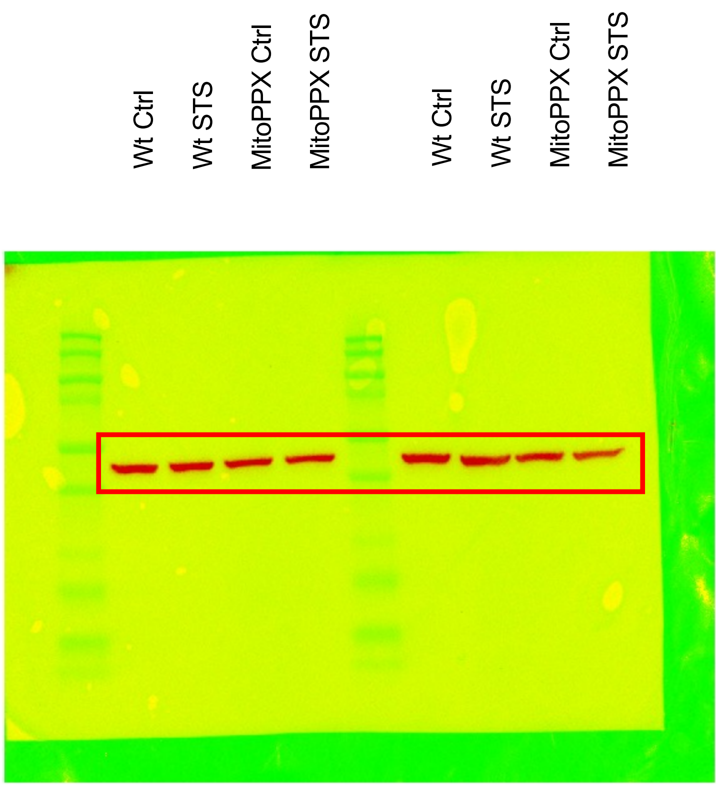

β-actin 2

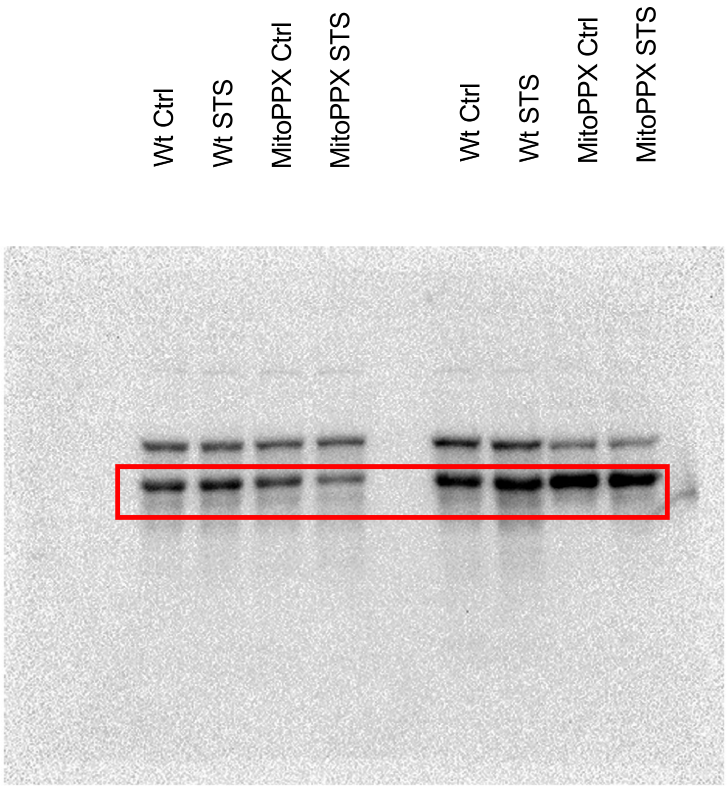

CREB 2

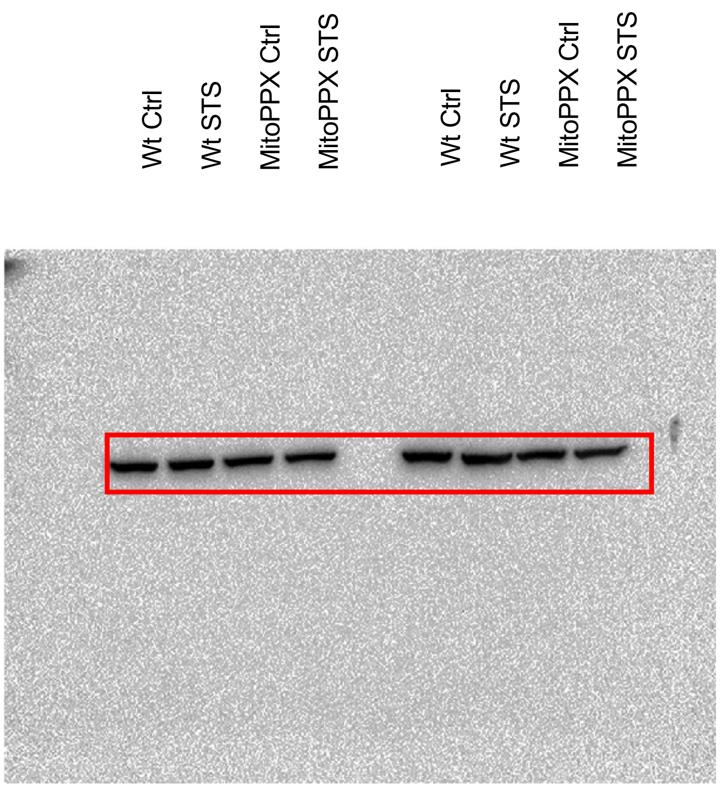

β-actin 2

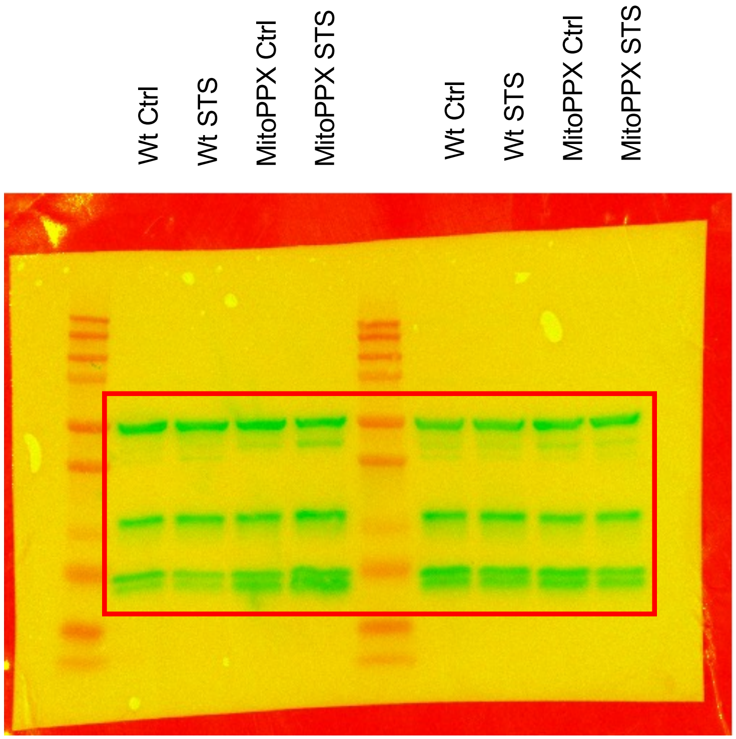

OXPHOS 1

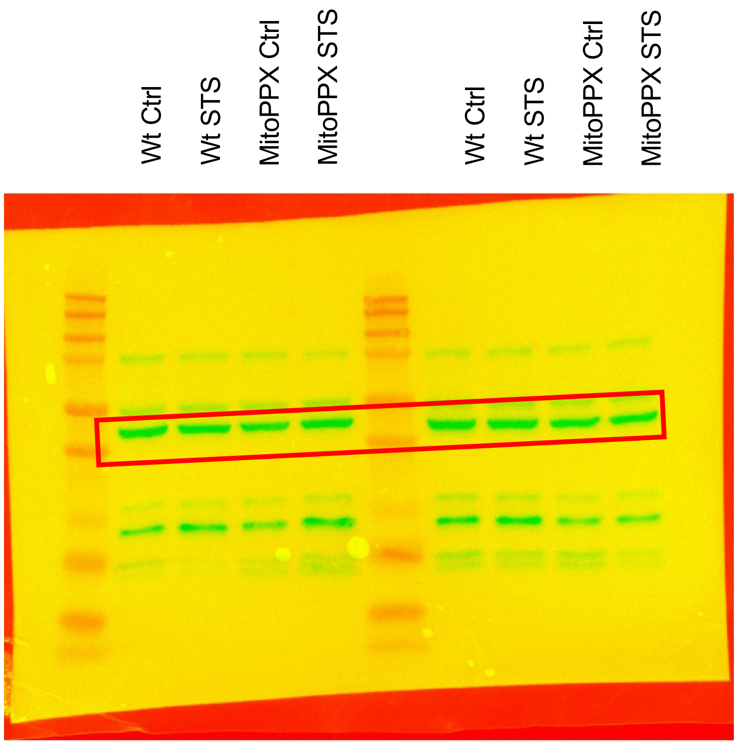

β-actin 1

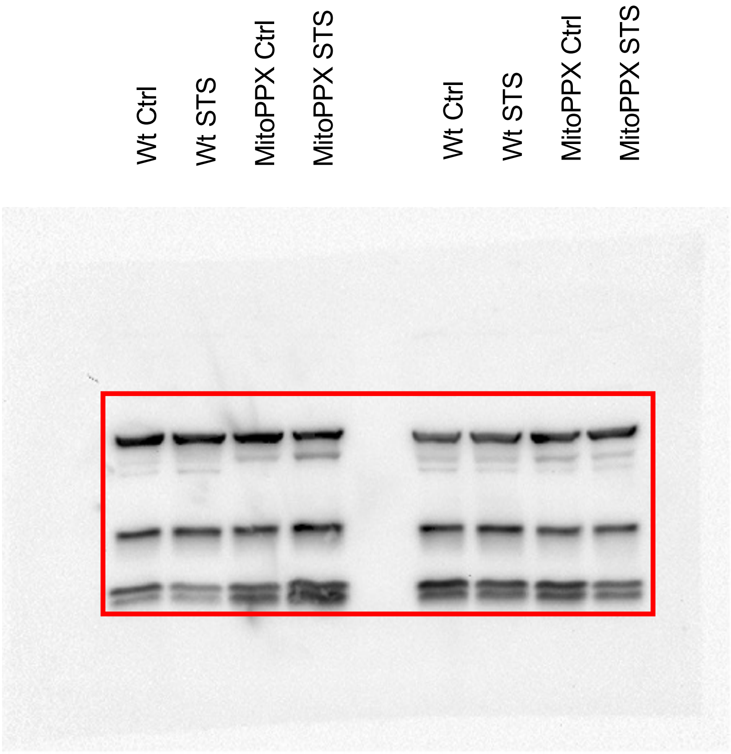

OXPHOS 1

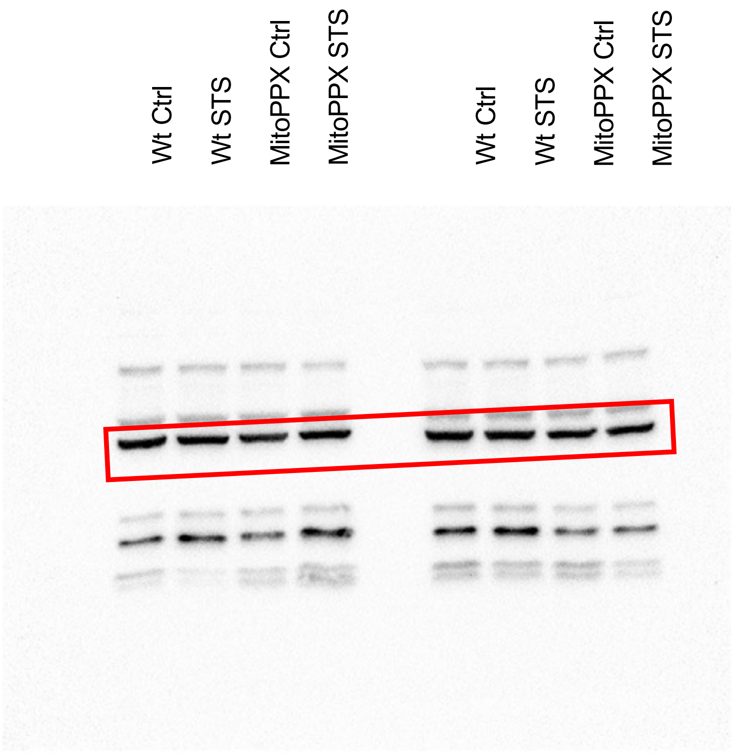

β-actin 1

Wt Ctrl
Wt STS
MitoPPX Ctrl
MitoPPX STS
Wt Ctrl
Wt STS
MitoPPX Ctrl
MitoPPX STS

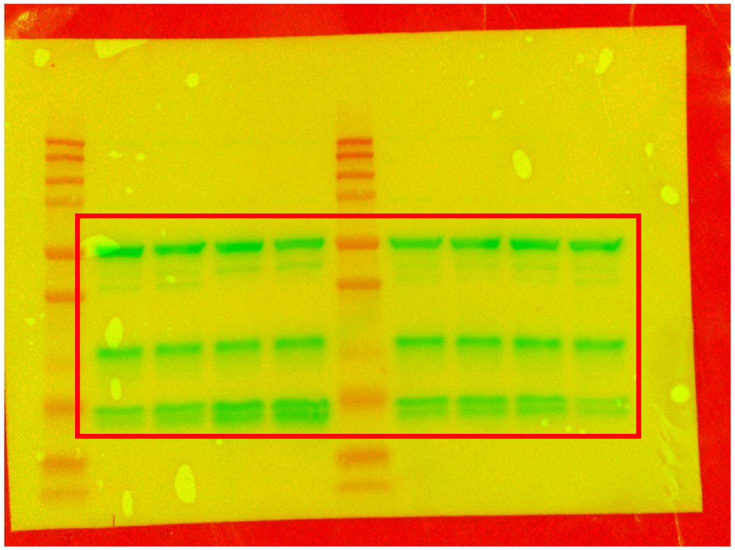

OXPHOS 2

Wt Ctrl
Wt STS
MitoPPX Ctrl
MitoPPX STS
Wt Ctrl
Wt STS
MitoPPX Ctrl
MitoPPX STS

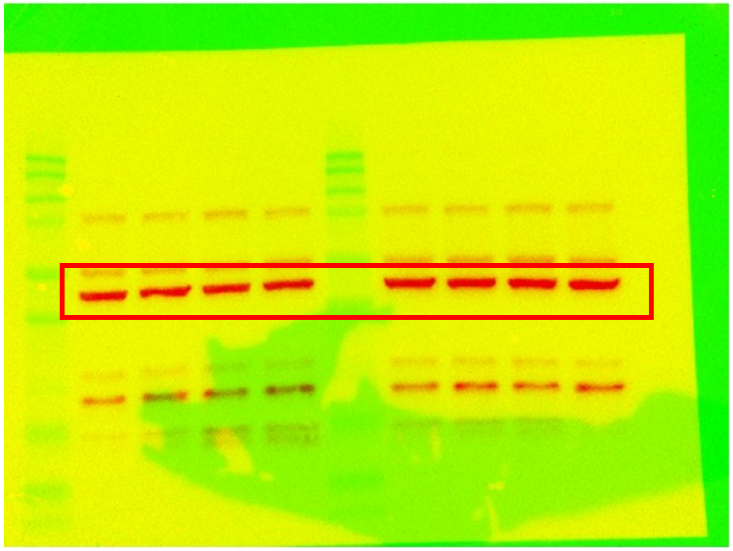

β-actin 2

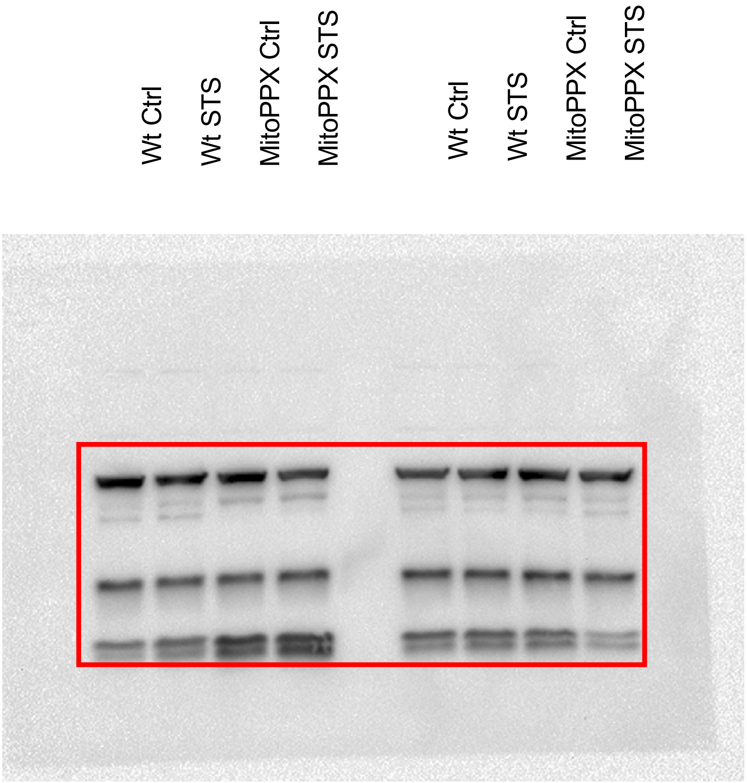

OXPHOS 2

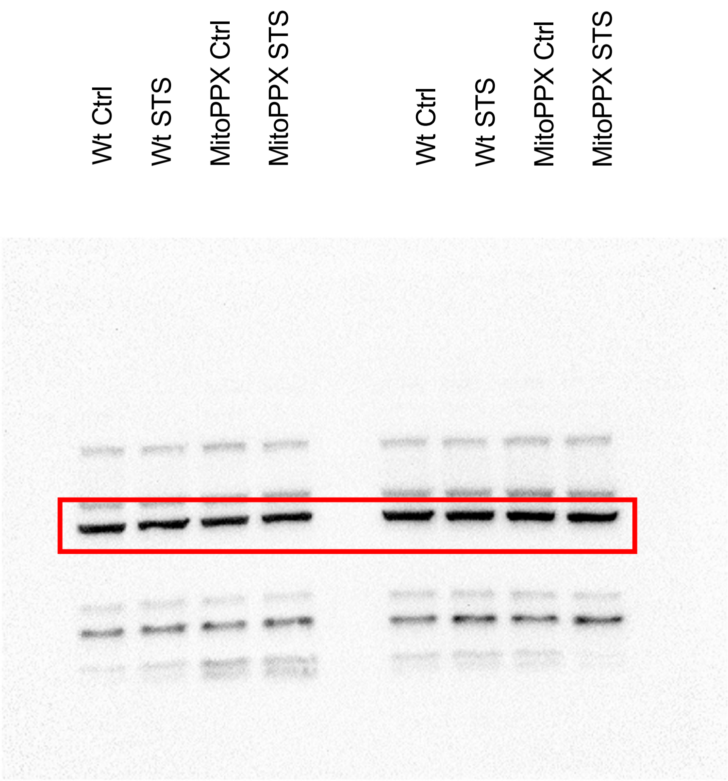

β-actin 2

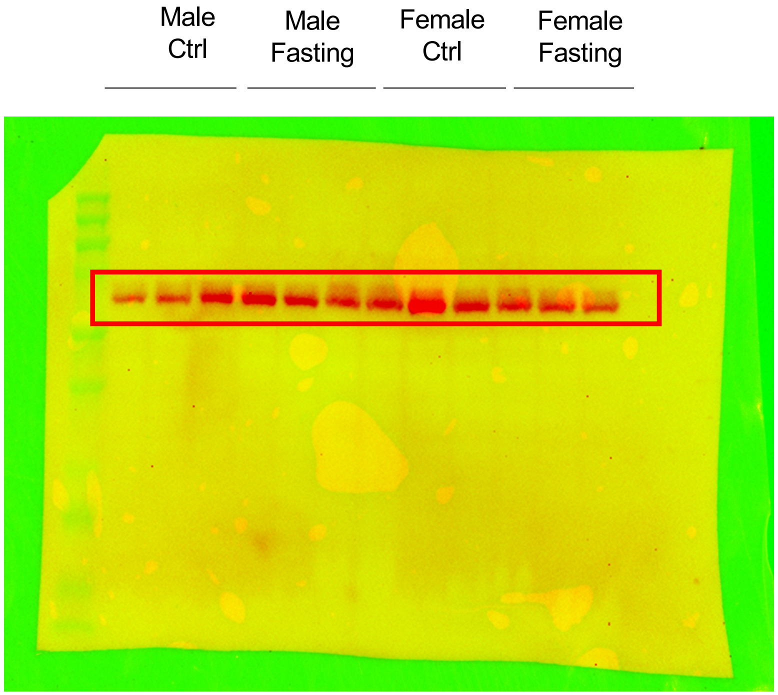

pAMPK (mice) 1

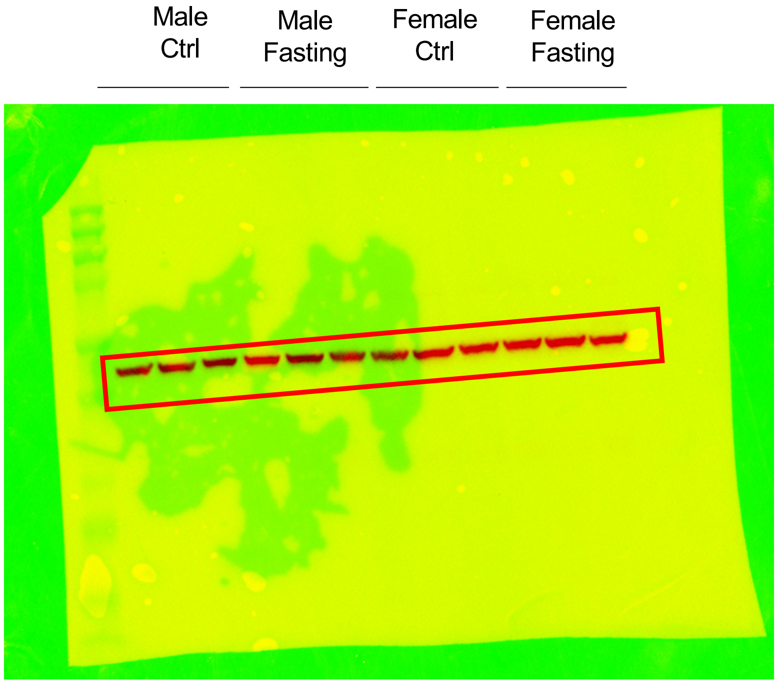

β-actin (mice) 1

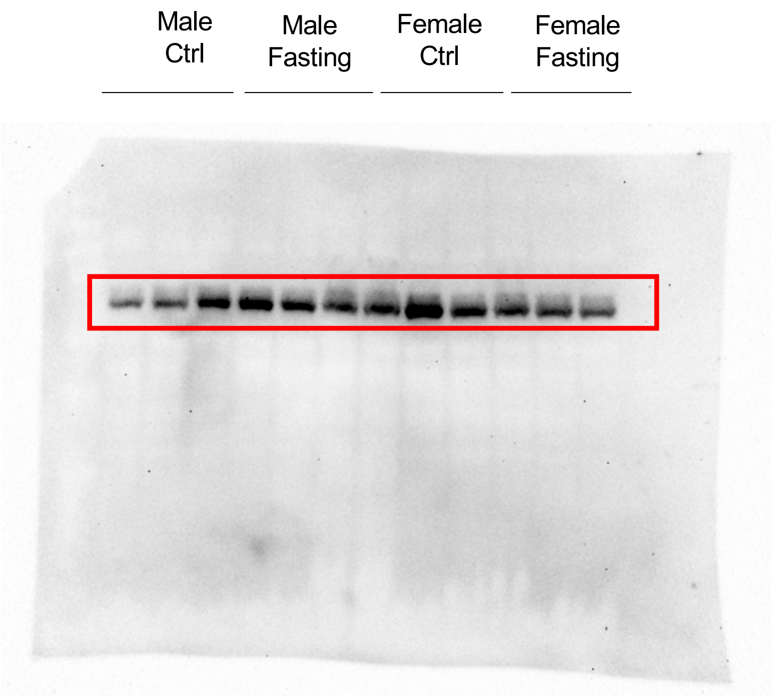

pAMPK (mice) 1

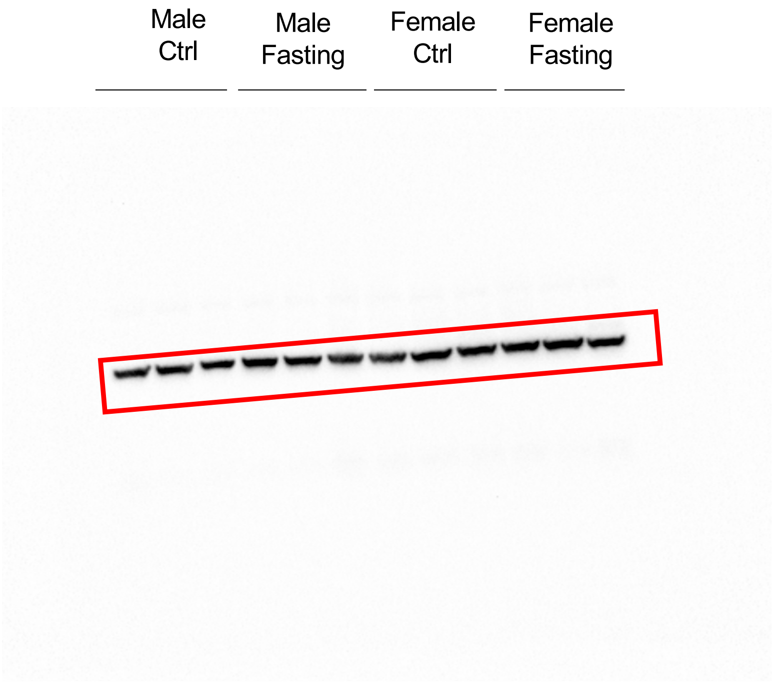

β-actin (mice) 1

Male Ctrl | Male Fasting | Female Ctrl | Female Fasting

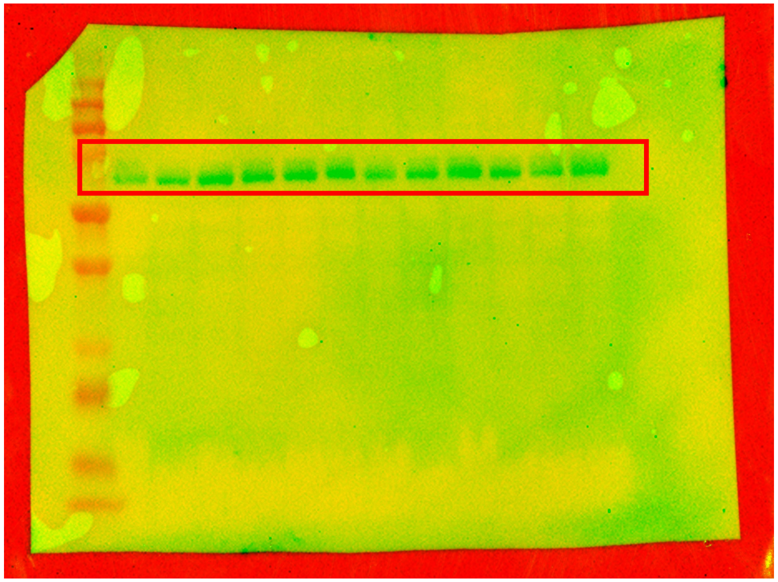

pAMPK (mice) 2

Male Ctrl | Male Fasting | Female Ctrl | Female Fasting

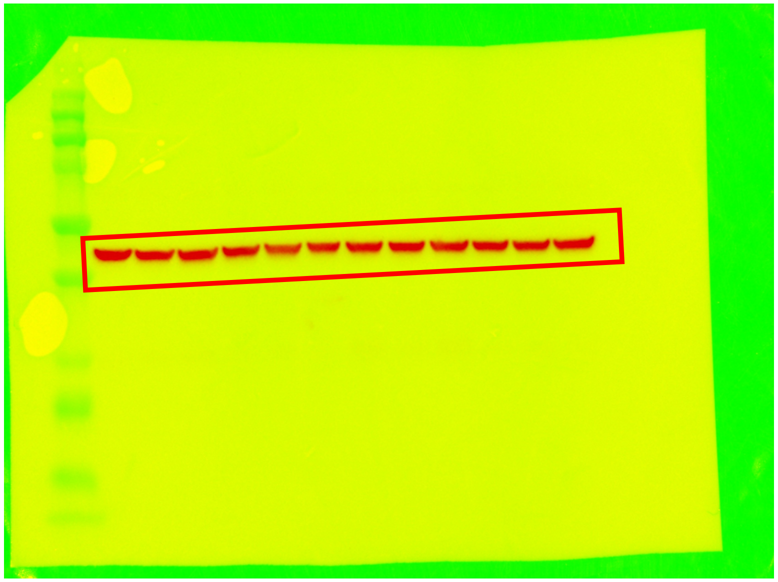

β-actin (mice) 2

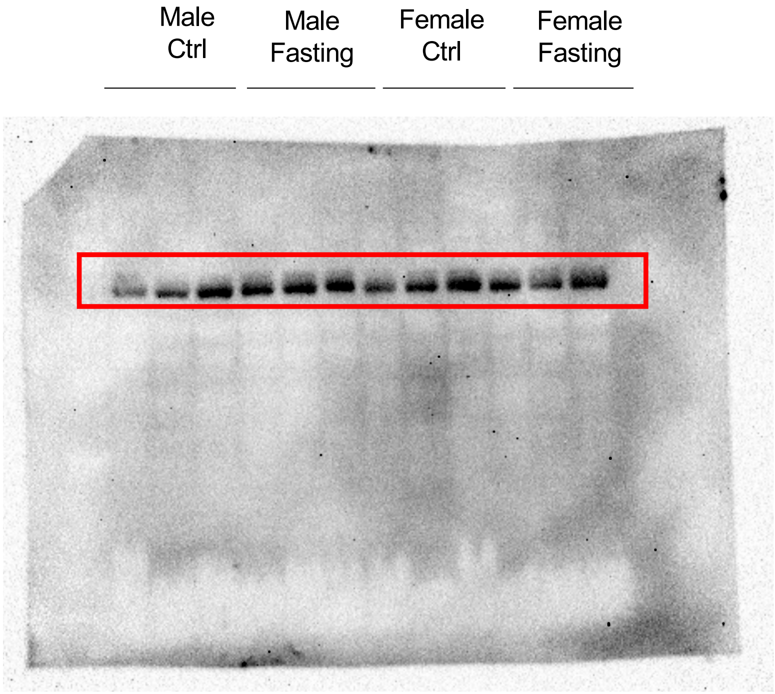

pAMPK (mice) 2

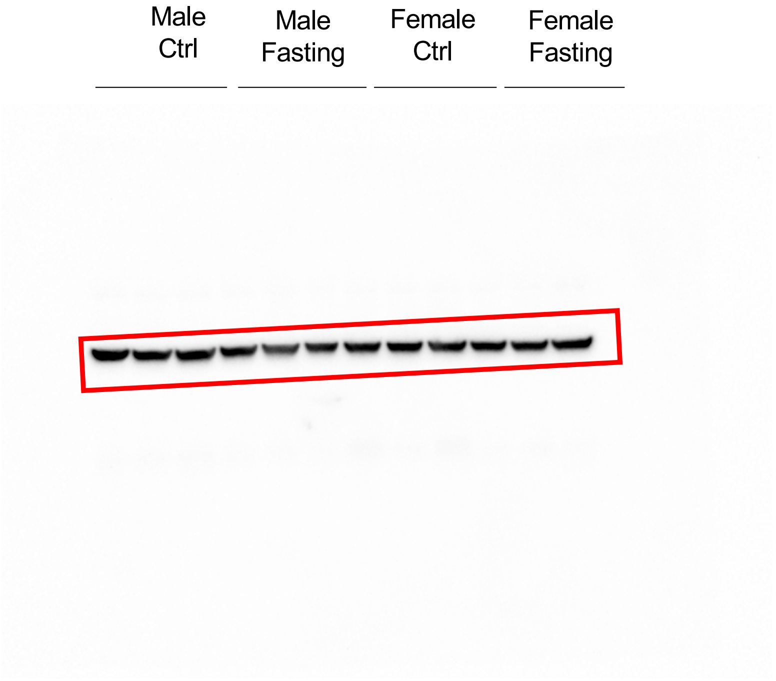

β-actin (mice) 2

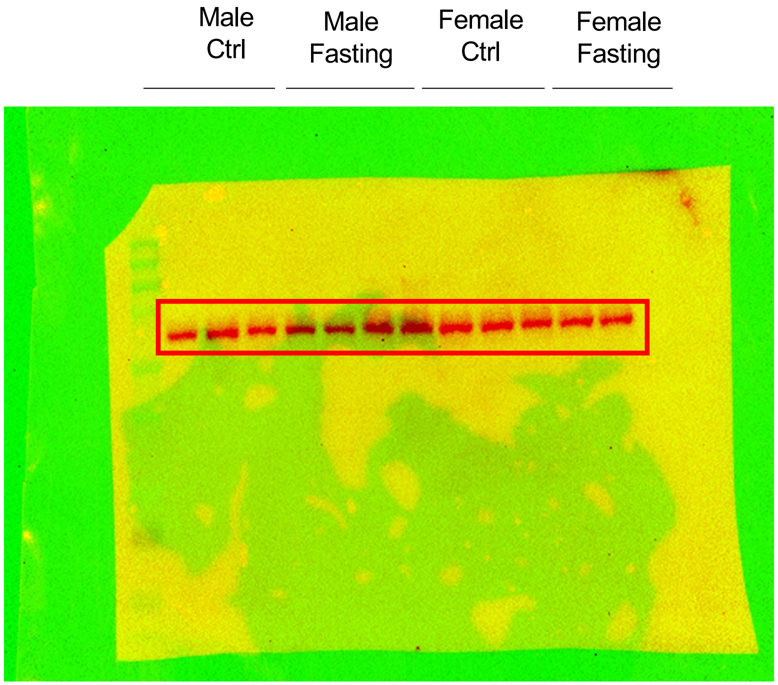

AMPK (mice) 1

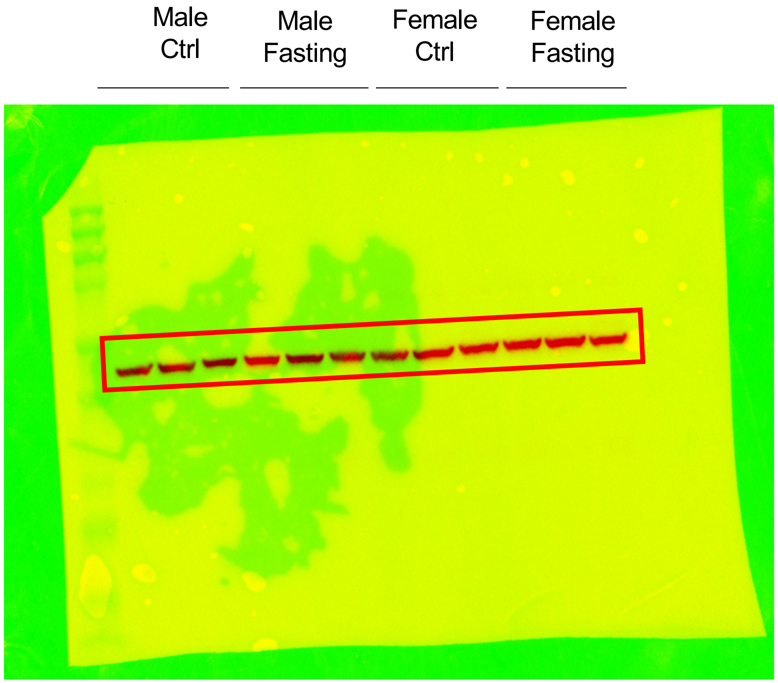

β-actin (mice) 1

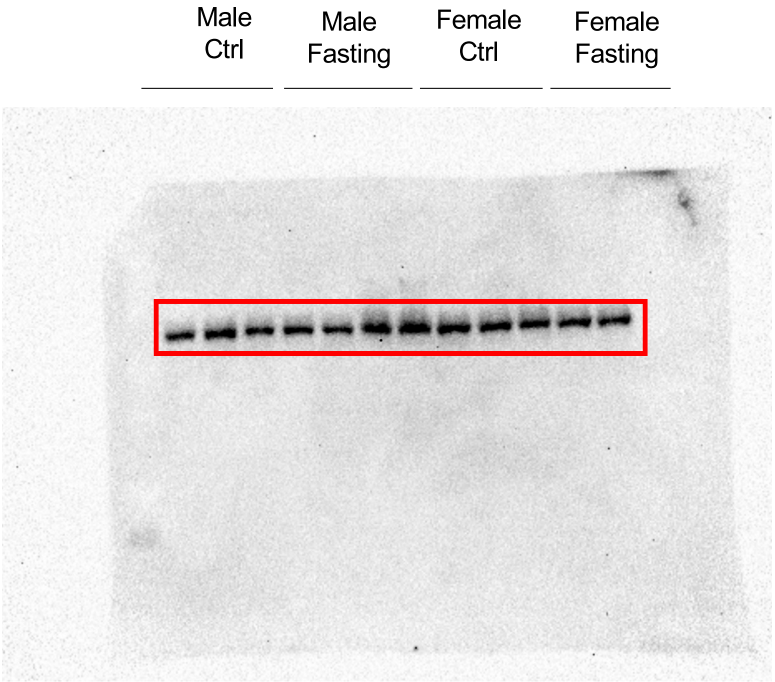

AMPK (mice) 1

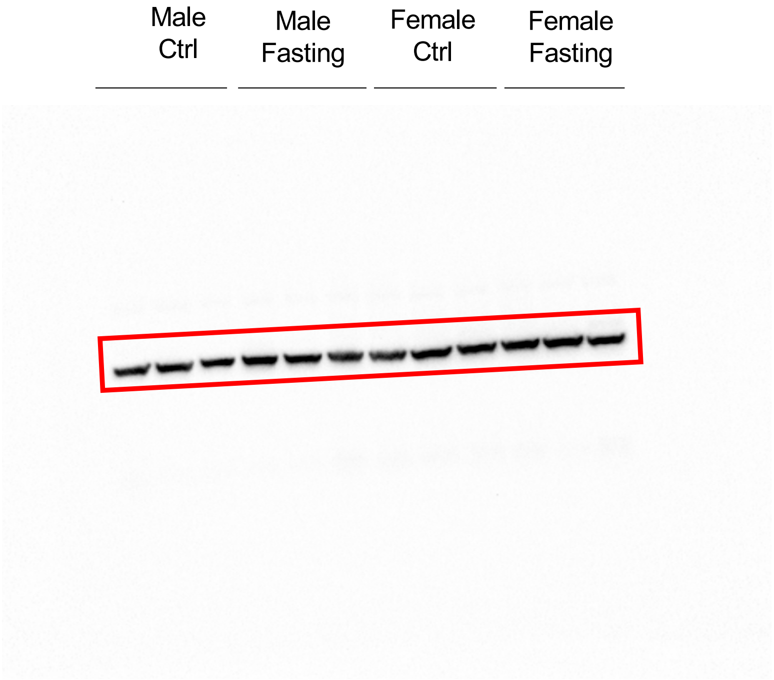

β-actin (mice) 1

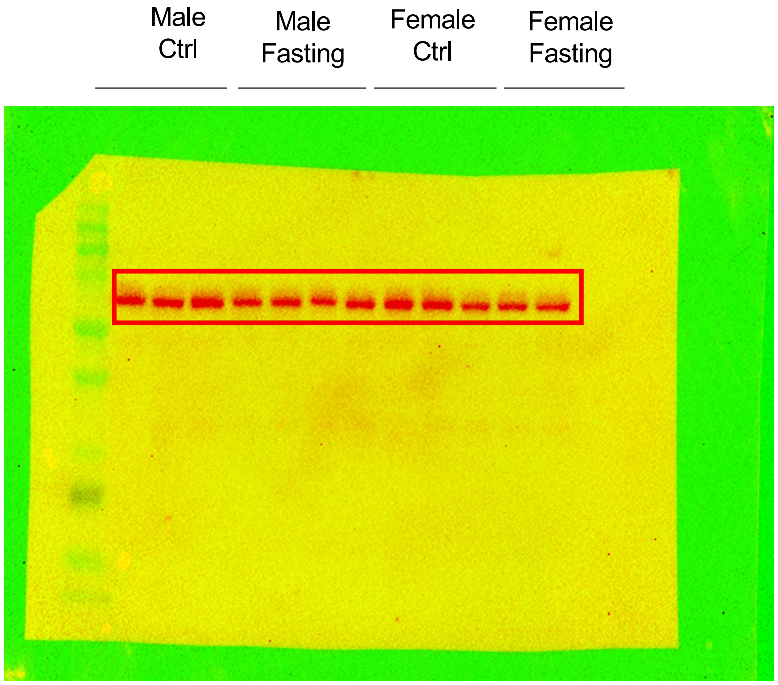

AMPK (mice) 2

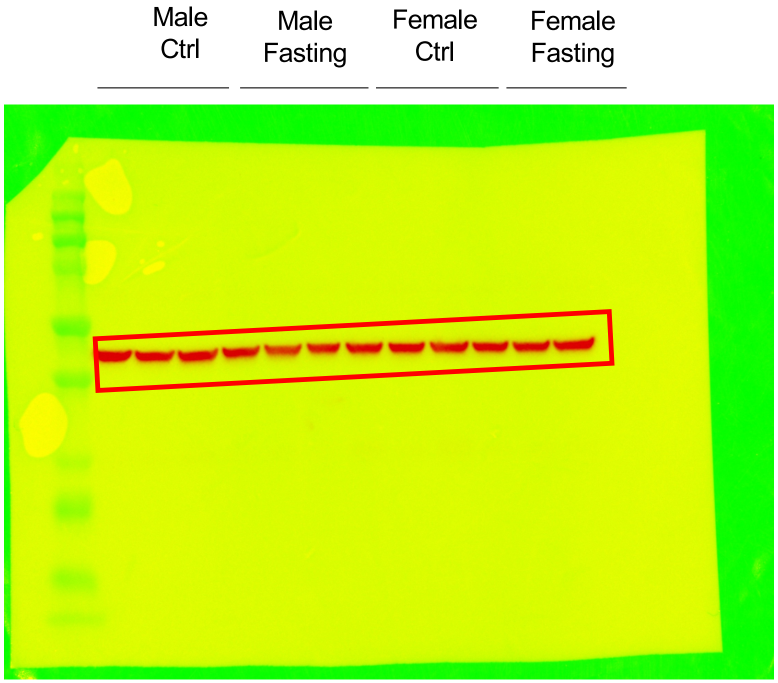

β-actin (mice) 2

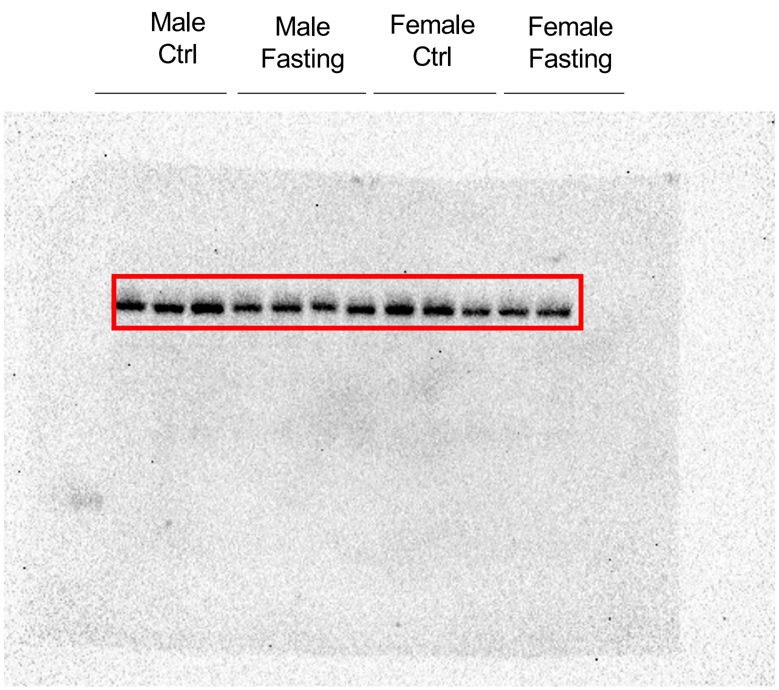

AMPK (mice) 2

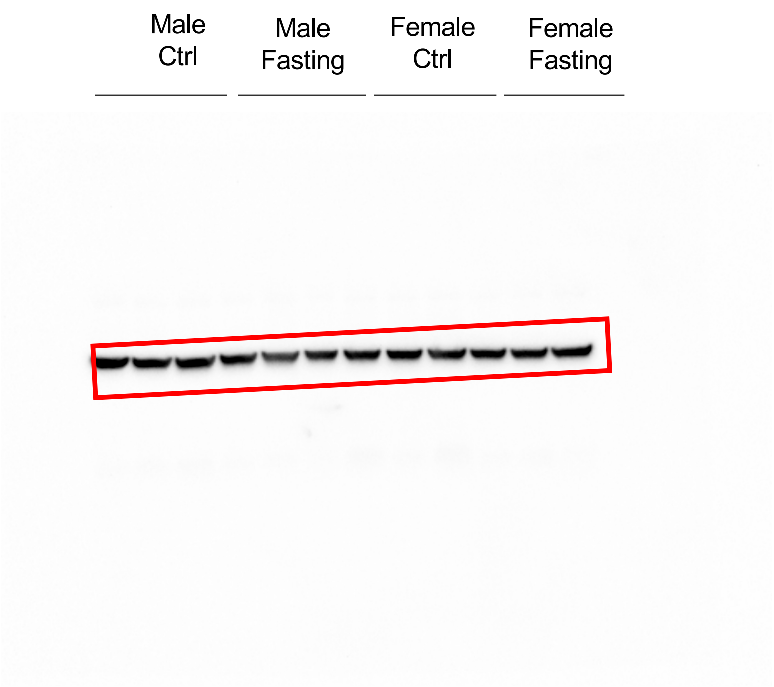

β-actin (mice) 2

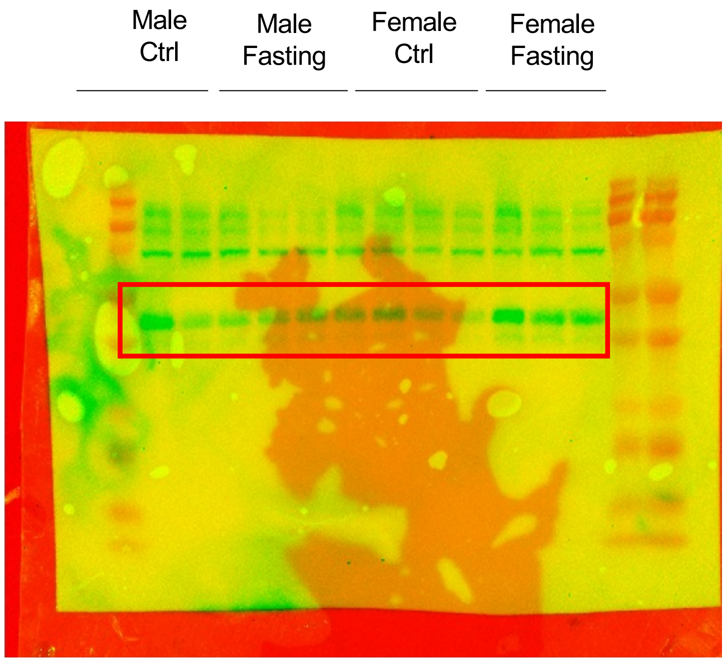

pCREB (mice) 1

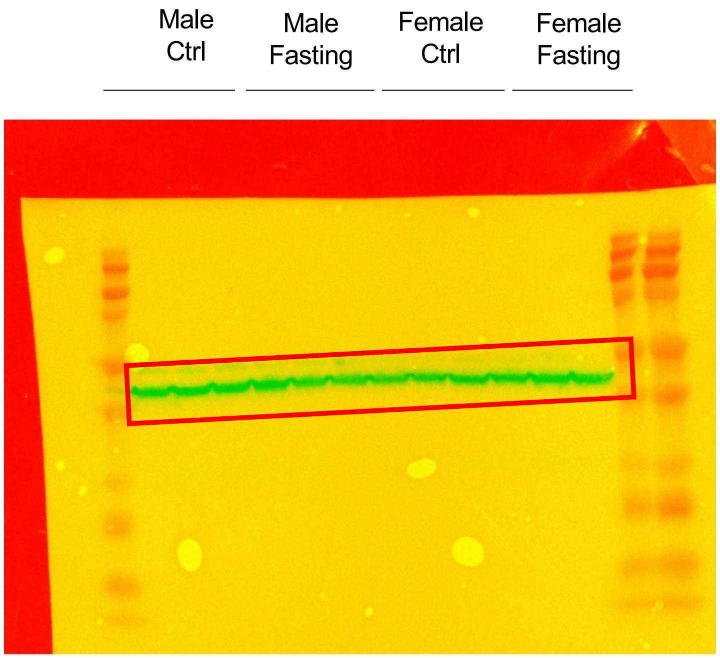

β-actin (mice) 1

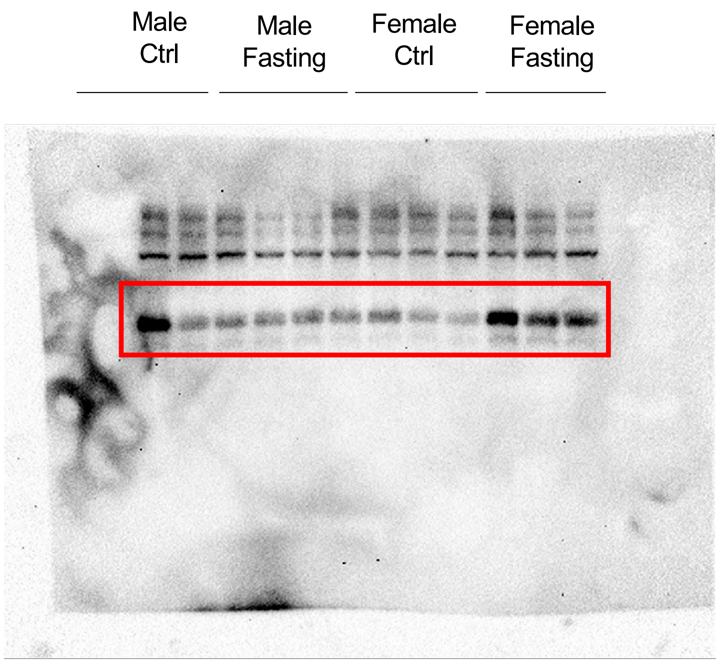

pCREB (mice) 1

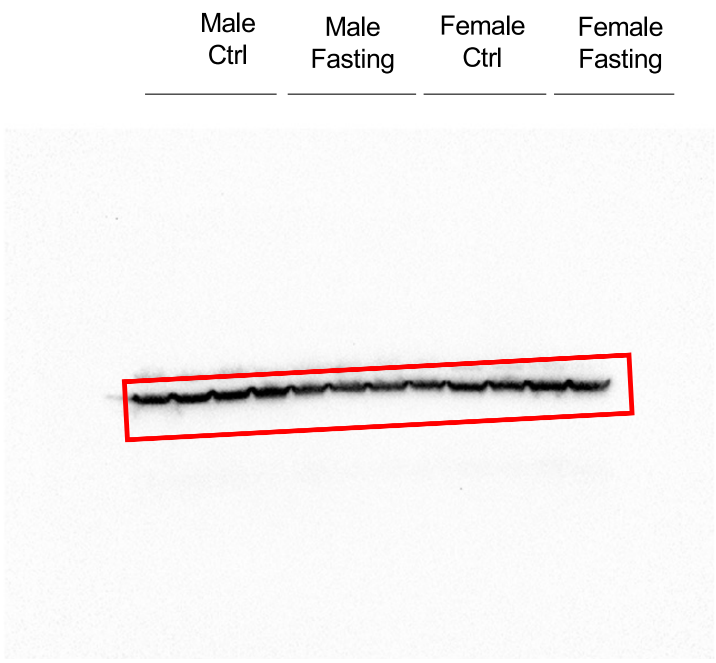

β-actin (mice) 1

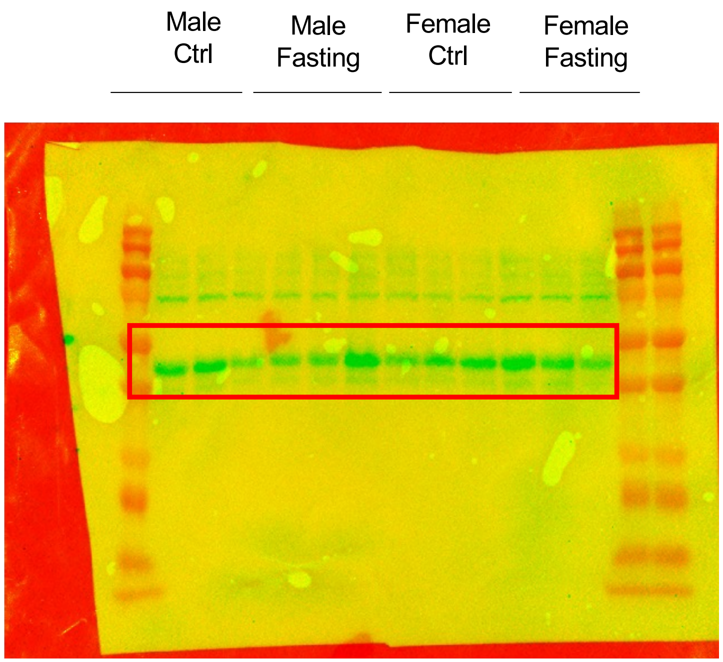

pCREB (mice) 2

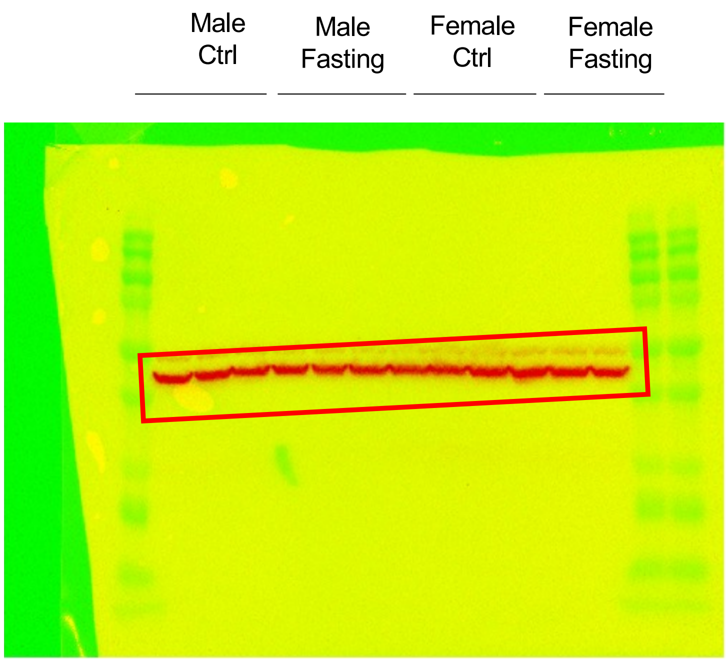

β-actin (mice) 2

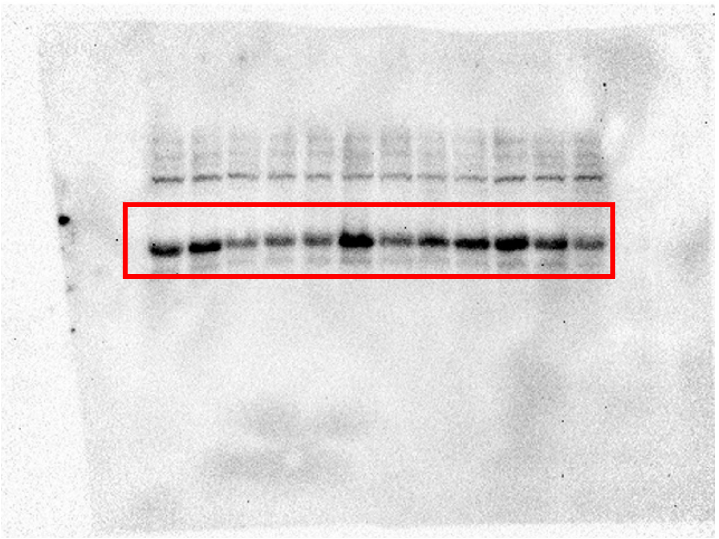

pCREB (mice) 2

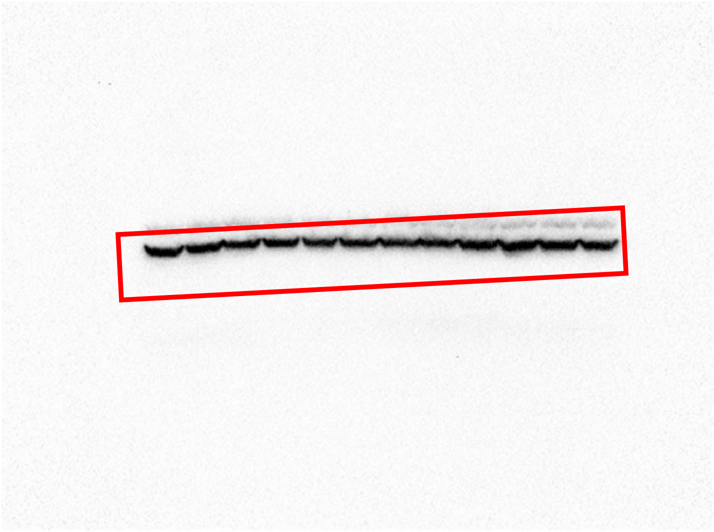

β-actin (mice) 2

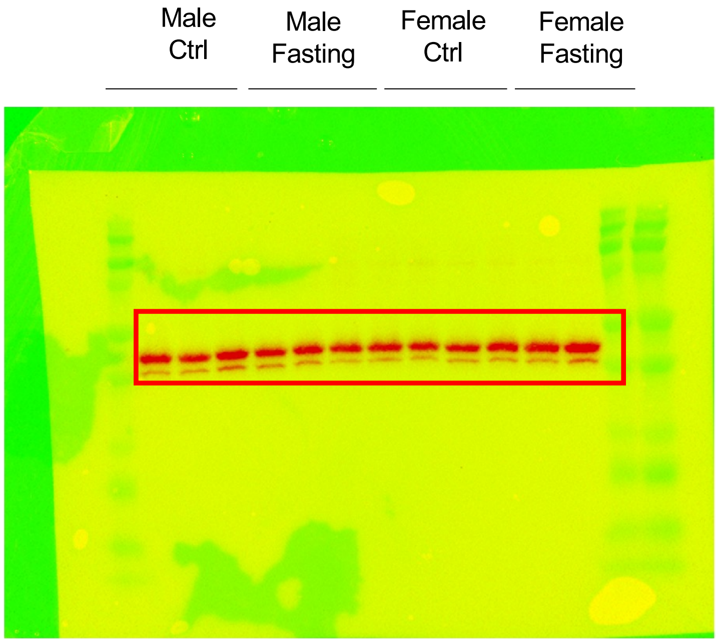

CREB (mice) 1

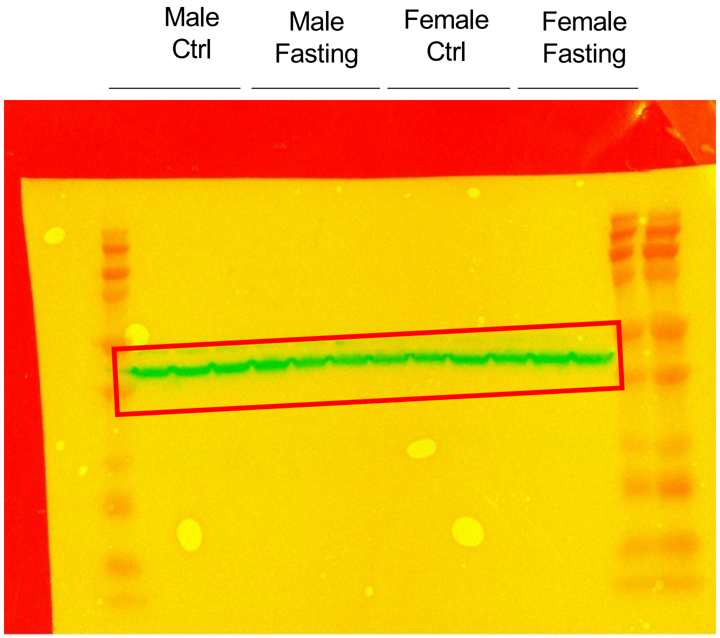

β-actin (mice) 1

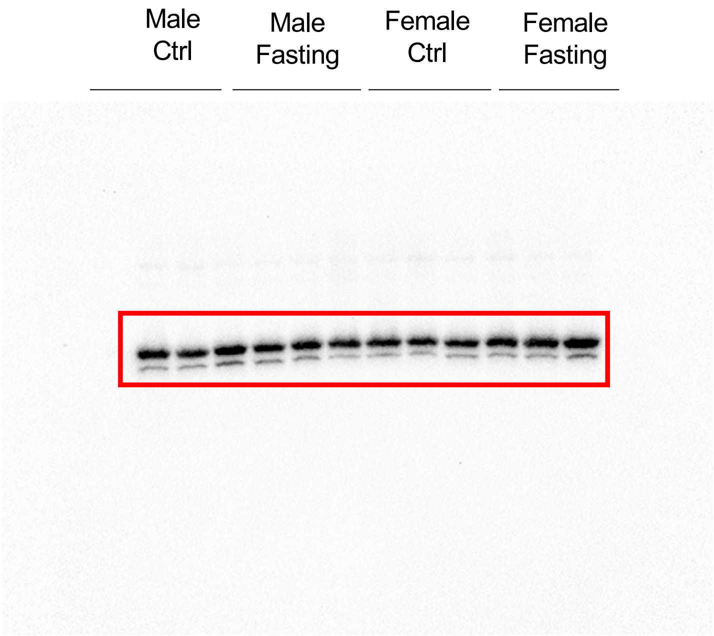

CREB (mice) 1

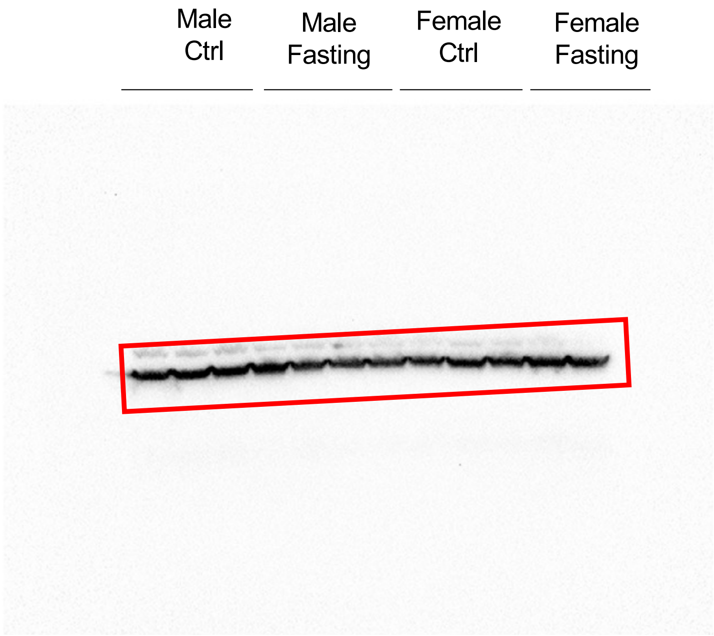

β-actin (mice) 1

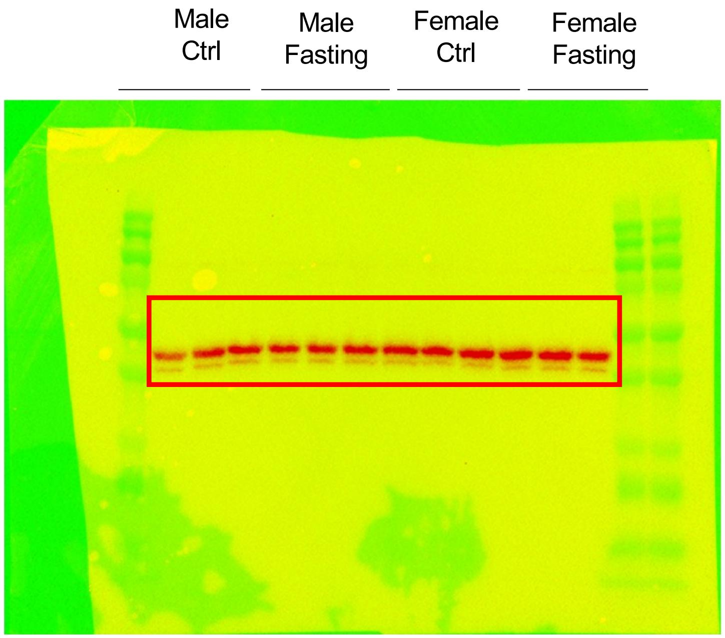

CREB (mice) 2

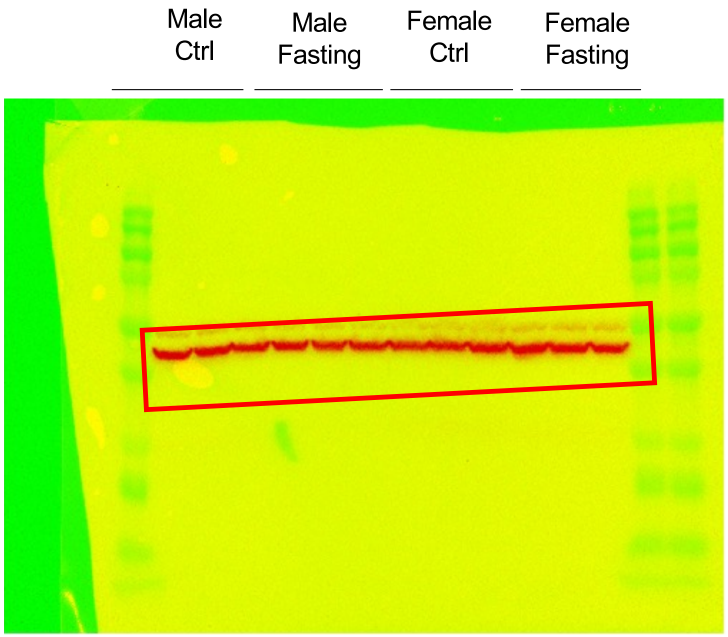

β-actin (mice) 2

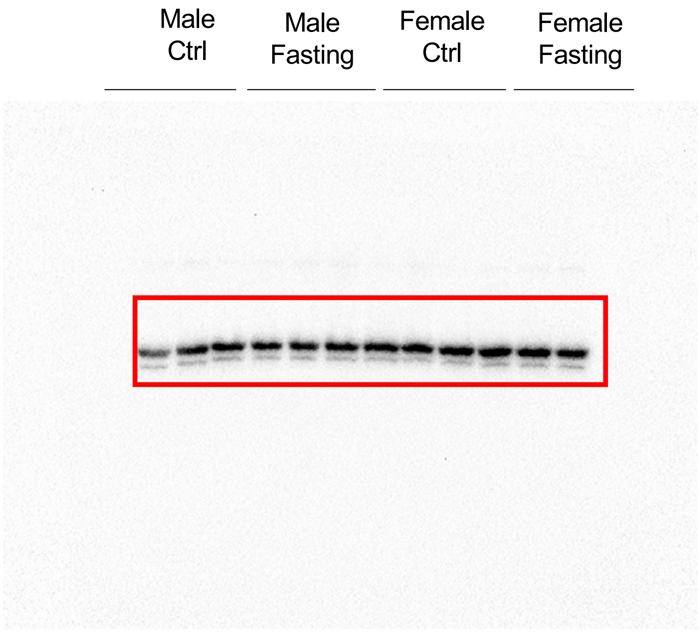

CREB (mice) 2

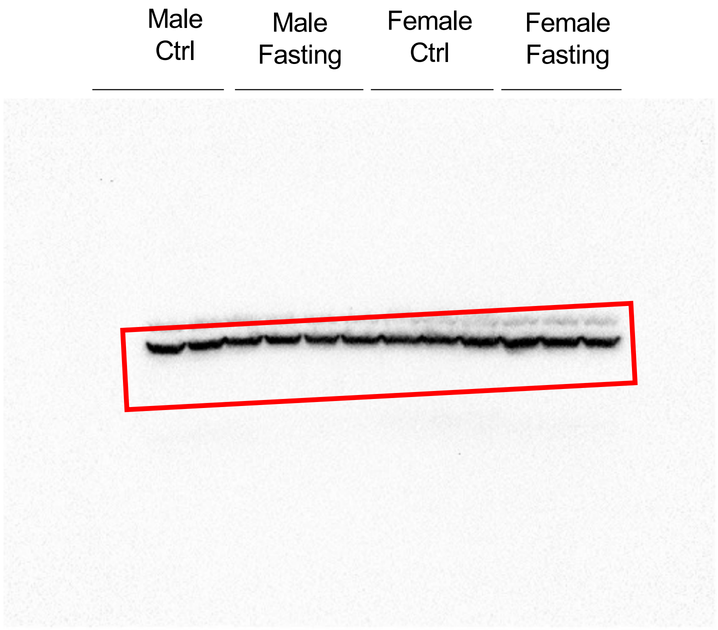

β-actin (mice) 2

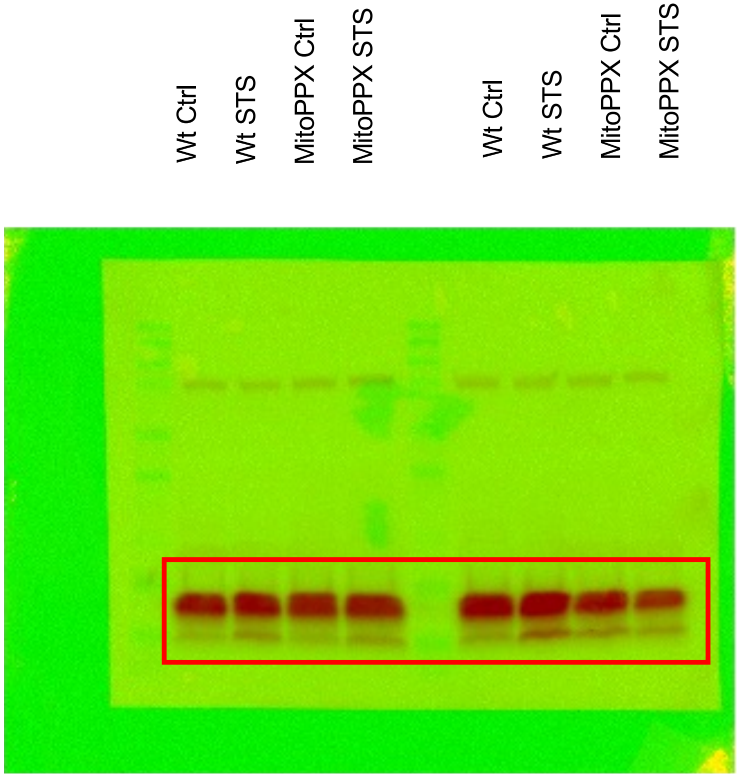

LC3B 1

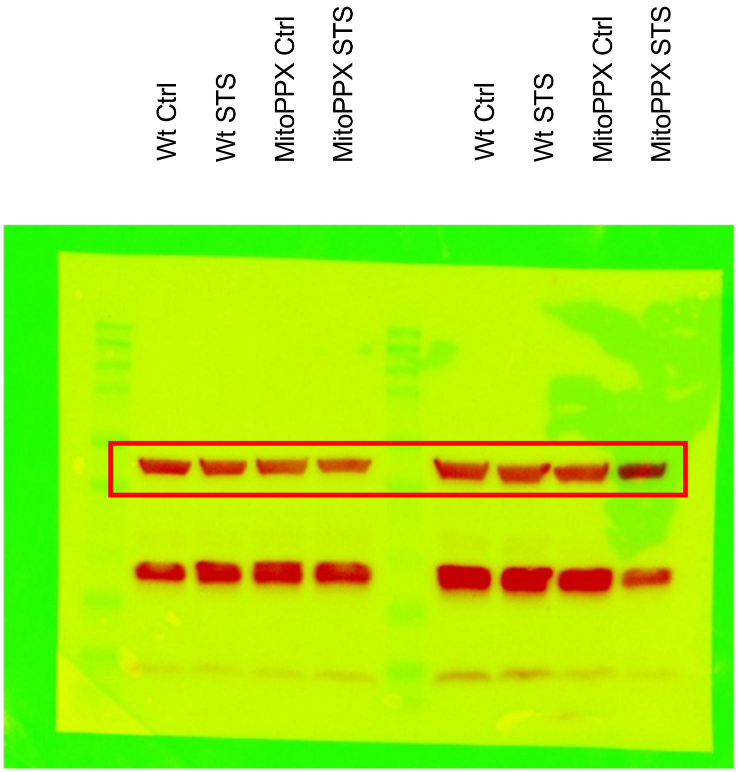

β-actin 1

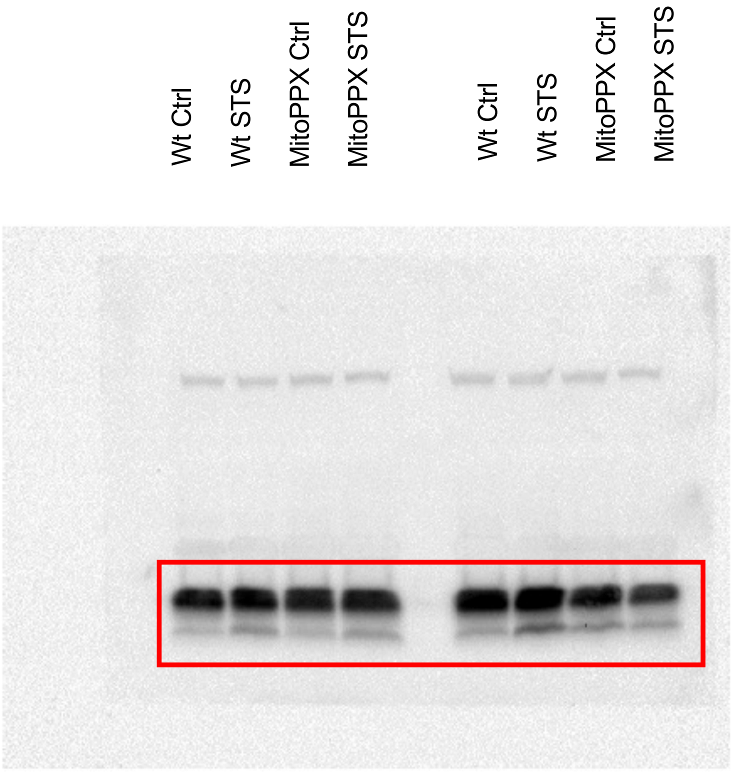

LC3B 1

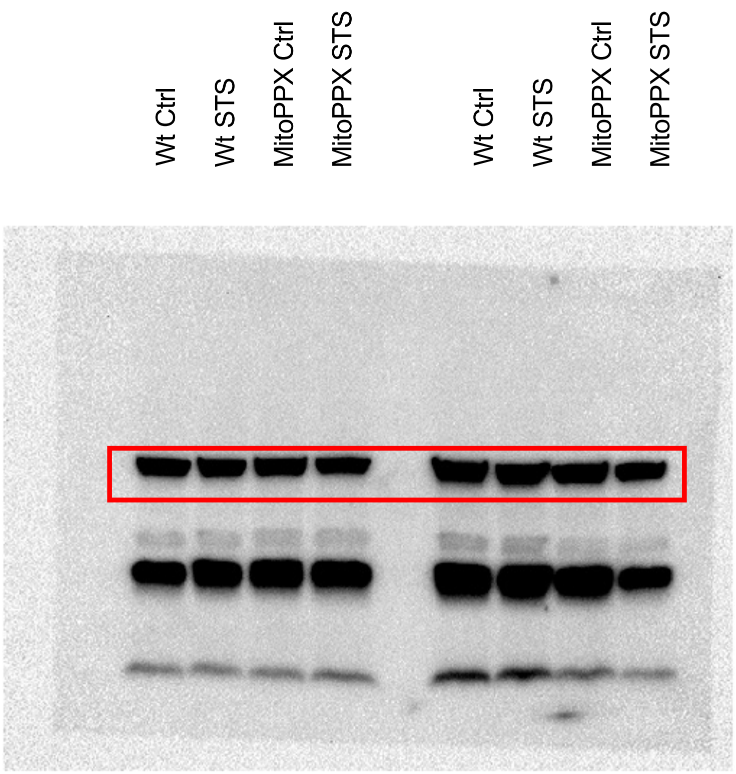

β-actin 1

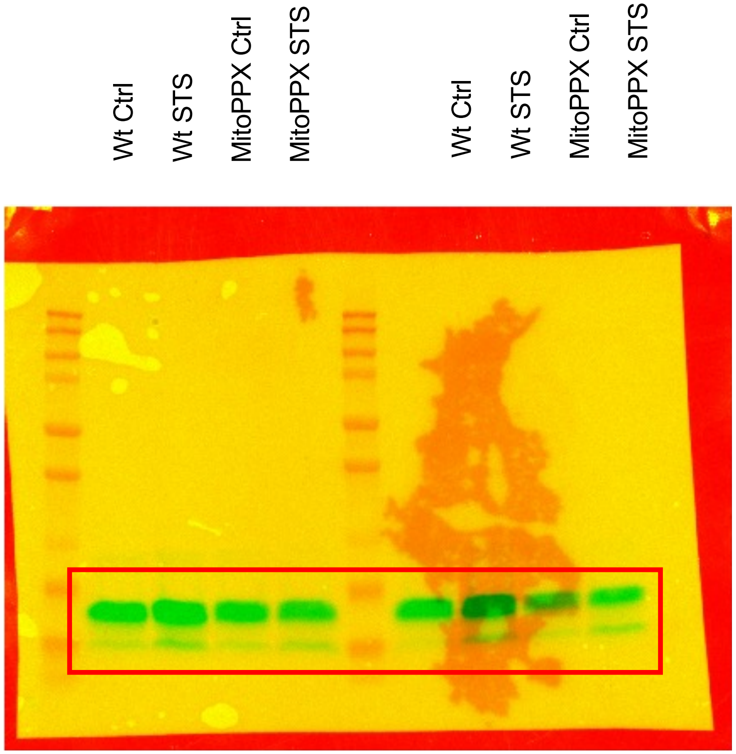

LC3B 2

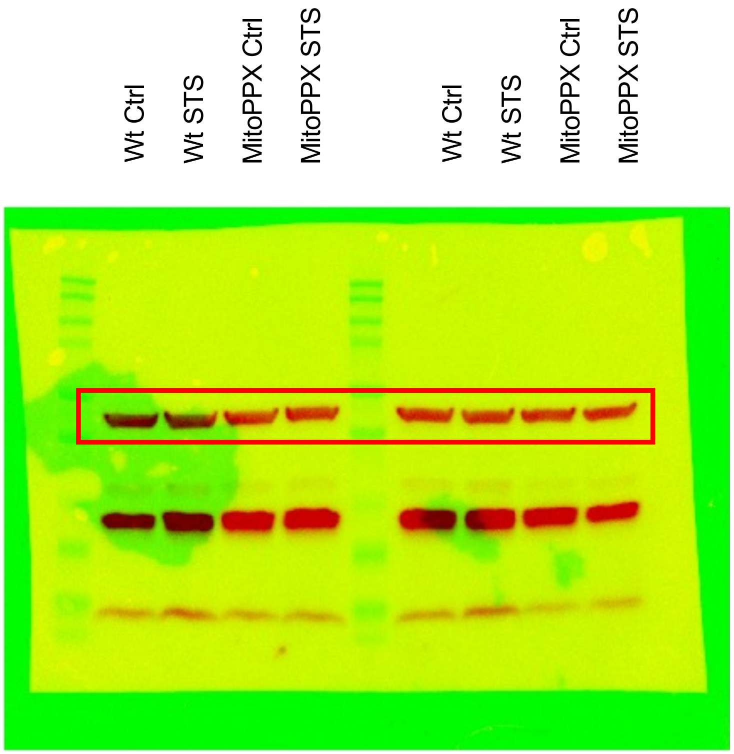

β-actin 2

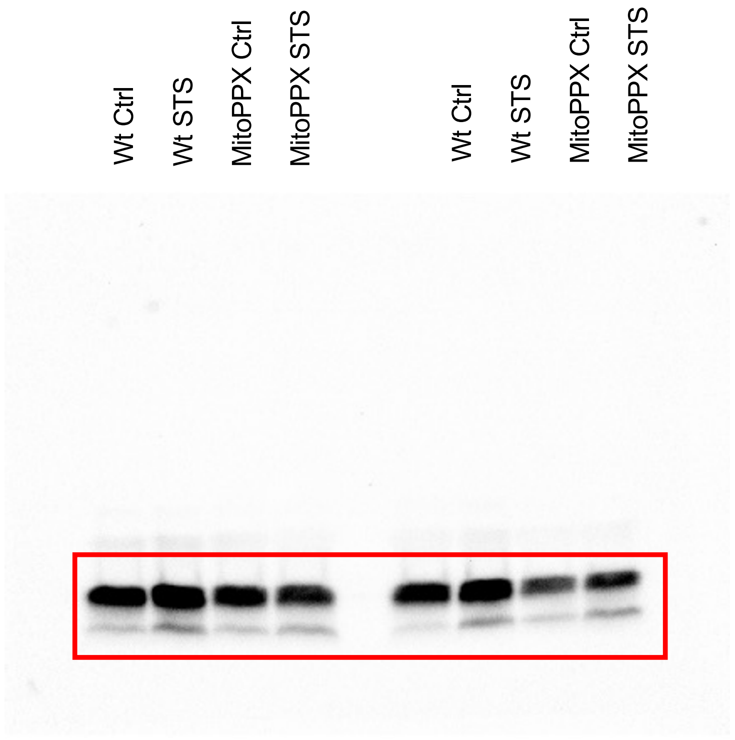

LC3B 2

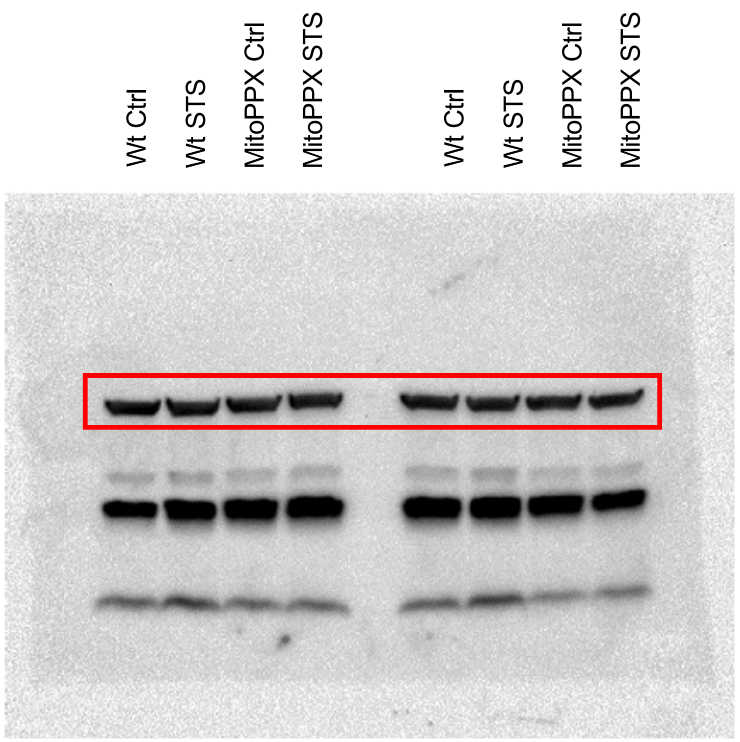

β-actin 2
