# Supplementary material for: Short‐term starvation activates AMPK and restores mitochondrial inorganic polyphosphate, but fails to reverse associated neuronal senescence
Source: Aging Cell. 2024 Aug 5;23(11):e14289. doi: 10.1111/acel.14289 (PMC11561667; doi:10.1111/acel.14289)
Supplement: Supplementary file 4 — Table S1. [file ACEL-23-e14289-s004.docx]

| ***MitoPPX Ctrl vs. Wt Ctrl*** | |
| --- | --- |
| **Reduced** | |
| Q96N67 | Dedicator of cytokinesis protein 7 |
| O43264 | Centromere/kinetochore protein zw10 homolog |
| Q9GZT9 | Egl nine homolog 1 |
| O95185 | Netrin receptor UNC5C |
| O60238-2 | BCL2/adenovirus E1B 19 kDa protein-interacting protein 3-like |
| P40692 | DNA mismatch repair protein Mlh1 |
| Q04656-2 | Copper-transporting ATPase 1 |
| Q92974-2 | Rho guanine nucleotide exchange factor 2 |
| Q9BTE3 | Mini-chromosome maintenance complex-binding protein |
| P63151-2 | Serine/threonine-protein phosphatase 2A 55 kDa regulatory subunit B alpha isoform |
| P29762 | Cellular retinoic acid-binding protein 1 |
| P42574 | Caspase-3 |
| Q9HA64 | Ketosamine-3-kinase |
| P37802-2 | Transgelin-2 |
| **Increased** | |
| Q7Z2D5-3 | Phospholipid phosphatase-related protein type 4 |
| A0A8Q3SJI5 | Tumor necrosis factor receptor superfamily member 6 |
| Q70J99 | Protein unc-13 homolog D |
| Q9H244 | P2Y purinoceptor 12 |
| J3QS47 | Cytochrome b561 |
| P27338 | Amine oxidase [flavin-containing] B |
| Q9BXI3 | Cytosolic 5'-nucleotidase 1A |
| P28161-2 | Glutathione S-transferase Mu 2 |
| P00750-3 | Tissue-type plasminogen activator |
| O94832 | Unconventional myosin-Id |
| Q9Y276 | Mitochondrial chaperone BCS1 |

**Supplementary Table 1.**

| ***MitoPPX STS vs. Wt STS*** | |
| --- | --- |
| **Reduced** | |
| P63151-2 | Serine/threonine-protein phosphatase 2A 55 kDa regulatory subunit B alpha isoform |
| Q9UPM8 | AP-4 complex subunit epsilon-1 |
| Q92547 | DNA topoisomerase 2-binding protein 1 |
| P02461 | Collagen alpha-1(III) chain |
| H0YL70 | TLE family member 3, transcriptional corepressor |
| P52799 | Ephrin-B2 |
| Q99453 | Paired mesoderm homeobox protein 2B |
| **Increased** | |
| Q9H244 | P2Y purinoceptor 12 |
| P27338 | Amine oxidase [flavin-containing] B |
| Q99496 | E3 ubiquitin-protein ligase RING2 |
| Q13232 | Nucleoside diphosphate kinase 3 |
| Q9NVS9 | Pyridoxine-5'-phosphate oxidase |
| Q96CB8 | Integrator complex subunit 12 |
| Q9NP66 | High mobility group protein 20A |
| P19404 | NADH dehydrogenase [ubiquinone] flavoprotein 2, mitochondrial |
| O95169-3 | NADH dehydrogenase [ubiquinone] 1 beta subcomplex subunit 8, mitochondrial |
| P56556 | NADH dehydrogenase [ubiquinone] 1 alpha subcomplex subunit 6 |
| Q9UDW1 | Cytochrome b-c1 complex subunit 9 |
| Q9Y666 | Solute carrier family 12 member 7 |
| Q9BYT3 | Serine/threonine-protein kinase 33 |
| O14949 | Cytochrome b-c1 complex subunit 8 |
| O95168-2 | NADH dehydrogenase [ubiquinone] 1 beta subcomplex subunit 4 |
| P17568 | NADH dehydrogenase [ubiquinone] 1 beta subcomplex subunit 7 |
| Q16795 | NADH dehydrogenase [ubiquinone] 1 alpha subcomplex subunit 9, mitochondrial |
| O75306 | NADH dehydrogenase [ubiquinone] iron-sulfur protein 2, mitochondrial |
